# Diversity of Cell Wall Related Proteins in Human Pathogenic Fungi

**DOI:** 10.3390/jof4010006

**Published:** 2017-12-29

**Authors:** Anna Muszewska, Sebastian Piłsyk, Urszula Perlińska-Lenart, Joanna S. Kruszewska

**Affiliations:** Institute of Biochemistry and Biophysics, Polish Academy of Sciences, 02-792 Warsaw, Poland; seba@ibb.waw.pl (S.P.); lenart@ibb.waw.pl (U.P.-L.); jsk@ibb.waw.pl (J.S.K.)

**Keywords:** cell wall proteins, cross-linking enzymes, fungal pathogen, fungal cell wall, glycohydrolase, glycosyltransferase

## Abstract

The cell wall is one of the major keys to fungal identity. Fungi use their cell wall to sense the environment, and localize nutrients and competing microorganism. Pathogenic species additionally modify their cell walls to hide from a host’s immune system. With the growing number of fungal infections and alarming shortage of available drugs, we are in need of new approaches to fight pathogens. The cell wall seems to be a natural target, since animal host cells are devoid of it. The current knowledge about fungal cell wall components is often limited, and there is huge diversity both in structure and composition between species. In order to compare the distribution of diverse proteins involved in cell wall biosynthesis and maintenance, we performed sequence homology searches against 24 fungal proteomes from distinct taxonomic groups, all reported as human pathogens. This approach led to identification of 4014 cell wall proteins (CWPs), and enabled us to speculate about cell wall composition in recently sequenced pathogenic fungi with limited experimental information. We found large expansions of several CWP families, in particular taxa, and a number of new CWPs possibly involved in evading host immune recognition. Here, we present a comprehensive evolutionary history of fungal CWP families in the context of the fungal tree of life.

## 1. Introduction

Fungal cell wall (CW) is a dynamic and complex structure which undergoes remodeling and modification during the cell life. Being an exoskeleton, it plays vital roles in fungal infection and recognition by the animal host’s immune system [1,2]. Fungi differ in their cell wall composition, depending on either cell development stage or cell type. Studies concerning model organisms, like *Saccharomyces cerevisiae*, *Candida albicans*, *Neurospora crassa*, *Schizosaccharomyces pombe*, and *Aspergillus fumigatus*, revealed that chitin, β-1,3-glucan, and glycoproteins constitute the conserved core of fungal CW, which, depending on taxon, is additionally complemented with other components e.g., β-1,6-glucan, chitosan, d-galactose, d-arabinose, d-galactosamine, mannose, uronic acid, melanin, α-1,3-glucan, and mixed β-1,3/1,4-glucan. During growth, the cell wall structure is extensively reshaped in the course of multiple reactions, catalyzed mainly by glucanases and chitinases. Among enzymes engaged in CW remodeling, chitinases and β-1,3 glucanases have been thoroughly studied. Others remain understudied, e.g., mixed glucan synthase and β-1,4 glucan synthases [3,4].

The fungal cell wall is composed of two layers. The internal one forms a scaffold, and is composed of chitin and β-1,3-glucan. The external one is usually formed by other polysaccharides and glycoproteins. Glycoproteins often possess a remnant of the GPI (glycosylphosphatidylinositol) anchor to attach via β-1,6-glucan to β-1,3-glucan. However, a different attachment mechanism must be employed in taxa lacking β-1,6-glucan. Cell wall proteins (CWPs) are glycosylated with *N*- and *O*-linked oligosaccharides.

Fungal cell wall can be considered as a dynamic organelle that mediates environment sensing, localization of nutrients and competitors, and last, but not least, fungal virulence [5,6]. The growth of fungal cells requires efficient trafficking, dedicated cytoskeletal structures, and organized vesicle systems, to build the new growing tip. Polarized growth is important, not only for nutrition, but also for penetration of the host and for infection development. Cell growth is also one of the means to escape from phagocyte engulfment. Colonization and degradation of new tissue areas is performed by growing fungal mass. This apical growth property is intrinsically connected with the cell wall, and considered one of the traits that historically define fungi [7]. The cell wall is important, not only for defining what is a fungus, but it also plays a pivotal role in mycoses. This brings us to a very practical aspect of researching the fungal cell wall. The treatment of severely ill patients often involves immunosuppression, which impacts phagocytes responsible for natural antifungal resistance. As a consequence, the number of people susceptible to fungal infection increases [8]. Almost half of 3 million invasive fungal infections per year, worldwide, have a fatal outcome, despite medical intervention, with a high death toll even in comparison to malaria or tuberculosis [9]. Also, immunocompetent individuals can develop an invasive fungal infection (IFI) following a physical trauma, where physical barriers to infection are omitted or disrupted.

Fungi, together with animals, form a common taxonomic unit called opisthokonta. Despite sharing many evolutionary traits, fungi, unlike other opisthokonta, are surrounded by a CW. This unique fungal feature can be exploited to target fungal cells with specific drugs, and avoid casting toxic effects on host cells. The majority of antifungal drugs are aimed at disturbing ergosterol, and only echinocandins target β-1,3-glucan synthesis [10]. Chitin synthesis-targeting drugs developed to date show low uptake, and thus, insufficient efficacy [3]. There are only five major classes of antifungal drugs available to treat fungal infections: polyenes, pyrimidine analogs, echinocandins, triazoles, and allylamines. However, many fungi are naturally resistant to certain antifungals, which additionally complicates treatment. For instance, mucoromycetes, causative agents of life-threatening mucormycoses, respond only to posaconazole and amphotericin B, while *Scedosporium* spp. Which infects lungs, sinuses, bones, joints, eyes, and brain [11], is resistant to all contemporary antifungals [12]. Taking into account both high mortality rate in the case of IFIs and ubiquitous fungal resistance, there is an alarming urgency for new antifungal agents [13].

The list of known pathogenic organisms, such as *Candida* spp., *Cryptococcus neoformans*, and *Aspergillus fumigatus*, these days, continuously grows to include newly emerging opportunistic fungal pathogens. Their responses to treatment with available drugs differs dramatically, but remain often understudied. Here, in order to provide a scaffold for systematic study on fungal cell wall, we identify and classify cell wall related proteins in human pathogenic fungi. Our results showing common traits in distinct human pathogens will inspire future searches for new cell wall related targets, beyond the currently used inhibitors of chitin synthase (nikkomycins) and β-1,3-glucan synthase (echinocandins).

## 2. Materials and Methods

Fungal proteomes were downloaded from NCBI (NIH, Bethesda, MD, USA) on 14 July 2017 [14]. Only proteomes of fully sequenced reference fungal genomes of 24 human pathogenic fungi spanning the taxonomic diversity were used. We searched the proteomes against all Carbohydrate-Active Enzyme (CaZy) database profiles (CNRS, Marseilles, France) [15] and additional Pfam profiles (EMBL-EBI, Cambridge, UK) [16], for proteins related to the CW. The latest dbCAN [17] edition (University of Georgia, Athens, GA, USA) released on 13 September 2017 includes Pfam hidden Markov model (HMM) profiles as old as of 2011. We constructed an updated edition of the HMM collection of CaZy enzymes [15] in house, adding (i) all updated 188 Pfam profiles present in the dbCAN, and (ii) new Pfam profiles for chitin synthases, GH16, GH2, GH128, GH63, GH64, GT90, and GH30 families. Sequence profiles of protein domains from Pfam 31 database were mapped using Pfam_scan.pl [16] as a wrapper for hmmscan [18] with a threshold of 0.00001. Alignments were built with Mafft 7 (linsi) [19], trimmed with trimAl [20], and trees were inferred with Phyml 3.0 [21] (LG model, 4 gamma, invariant sites). Protein domain architectures were obtained by scanning the proteins against Pfam 31 with Pfam_scan.pl with a e-value threshold of 0.01. Phylogenetic trees with domain architectures were visualized with Interactive Tree Of Life (iTOL) (EMBL, Heidelberg, Germany) [22]. Each protein family was inspected to differentiate between related enzymes. Laccases were identified using, as reference, Hoegger classification [23].

Biochemical maps were constructed based on literature searches, CaZy resources [15], Kyoto Encyclopedia of Genes and Genomes (KEGG) maps (Kyoto University, Kyoto, Japan) [24], and EC enzyme descriptions.

## 3. Results and Discussion

### 3.1. The Dataset

**Figure 1:**
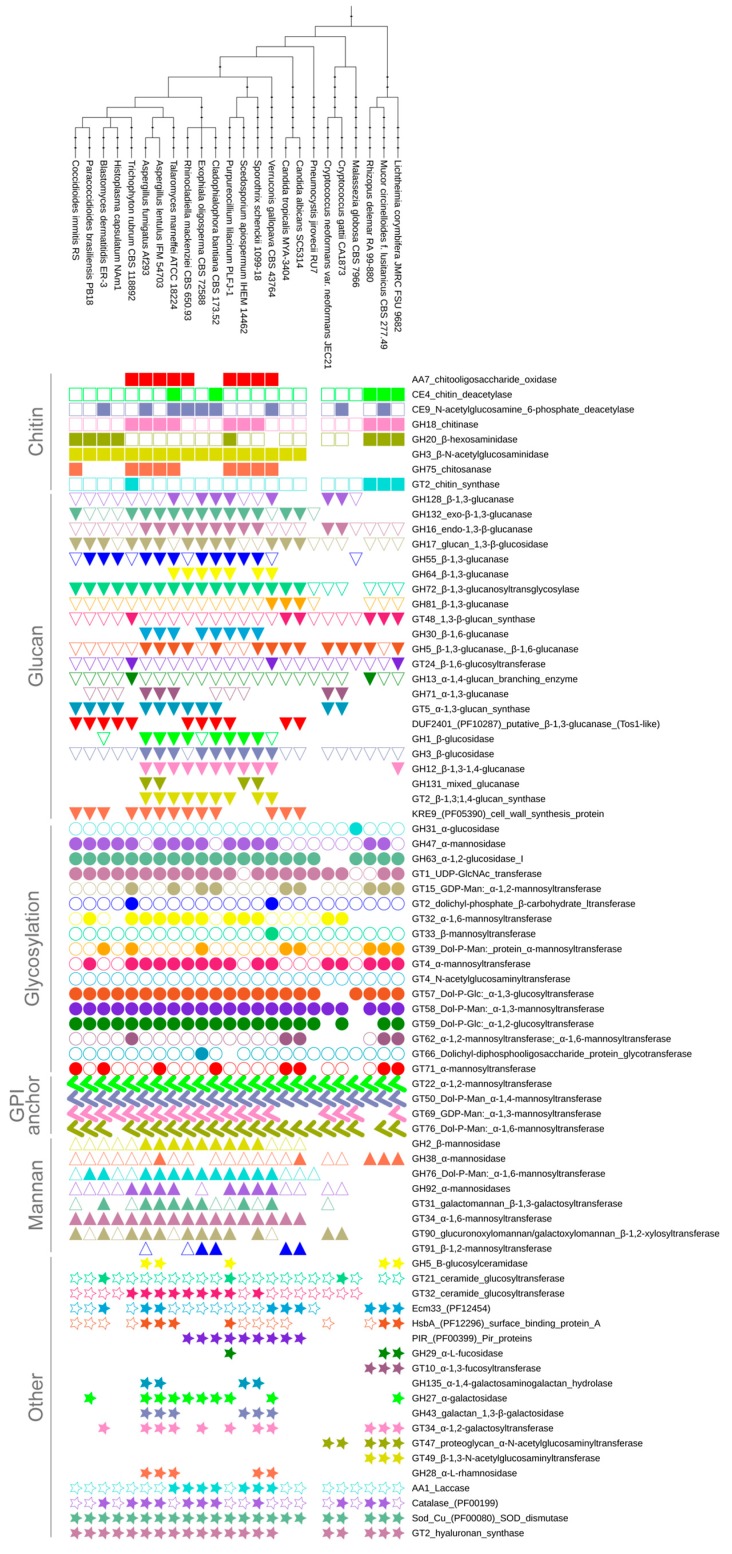
Summary of distribution of 78 CWP families in diverse pathogenic fungi. Filled shape—more homologs than average; empty shape—average number or less homologs; no shape—no homologs.

In order to identify all cell wall related enzymes in human pathogenic fungi, we carried out searches with all CAZy and 197 Pfam 31 profiles representing CWPs against 24 fungal proteomes (the list of proteomes is available in Supplementary Table S1A, and characteristics of selected protein families in Supplementary Table S1B). The identified CW related Pfam and Cazy protein families were split during the analyses into subfamilies, based on their predicted function, e.g., family GT2 groups, not only chitin synthases, but also dolichyl-phosphate carbohydrate transferases. This resulted in a collection of 78 families spanning 4000 proteins potentially related to the cell wall (for dataset summary see Supplementary Table S1C, all sequence identifiers, and phylogenetic trees are provided in Supplementary Datasets S1–S3). Our results demonstrate that there is no fungus that encodes representatives of the whole ensemble of 78 CWP families; on average, fungi have CWPs belonging to 57 families (Figure 1). The common repertoire of 27 CWPs (and only 12 if pneumocystis is included) outline a well-defined core, with a high chance for being similar in most fungi, regardless of their ecology. This core machinery includeswell-studied enzymes, such as glucanases, glucosidases, chitinases, chitin synthases, and glucan synthases necessary to maintain buried cell wall layers, but also mannosyl transferases needed for modifications to be exposed, outer CW parts. Importantly, the total number of CW proteins is strongly correlated with proteome size (*r* = 0.73) and moderately correlated with genome size (*r* = 0.58), which may result from genetic drift and simple genome inflation.

### 3.2. Cell Wall Components

#### 3.2.1. Chitin

**Figure 2:**
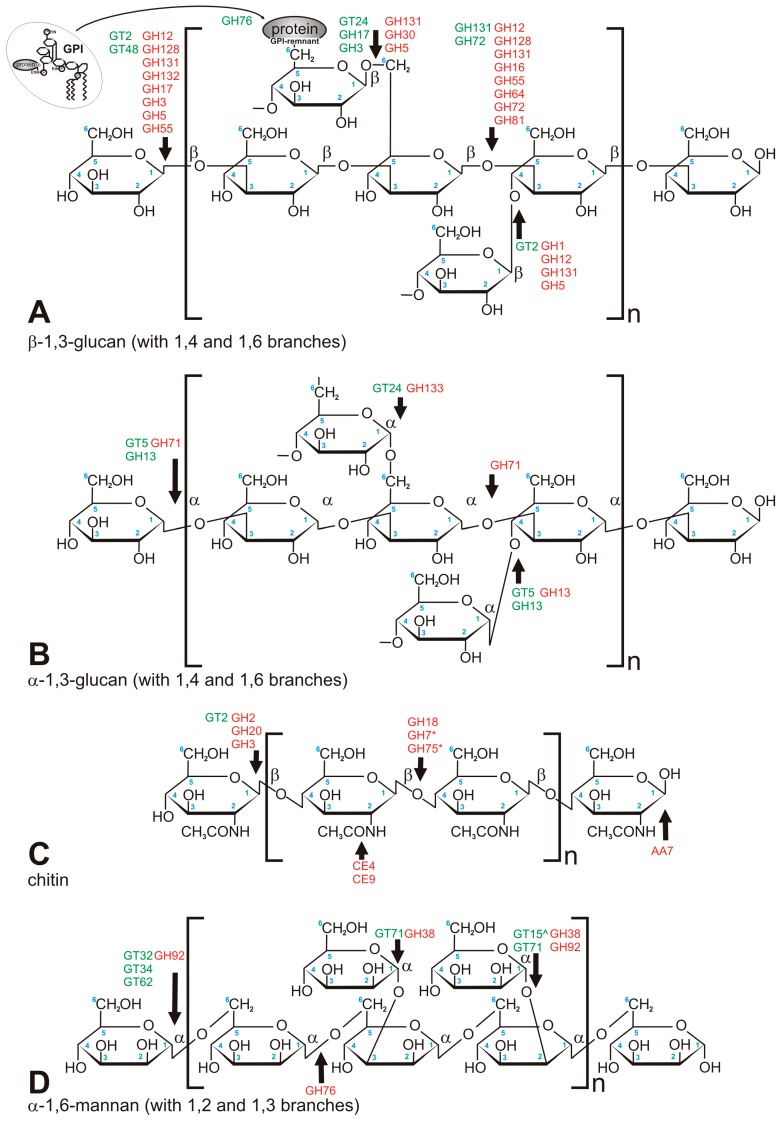
Major carbohydrate polymers which build fungal cell wall. (**A**) β-1,3-glucan (crosslinked via 1,4 and 1,6 chain branches); (**B**) α-1,3-glucan (crosslinked via 1,4 and 1,6 chain branches); (**C**) chitin; (**D**) mannan, mostly found in Saccharomycetales. Green font refers to anabolic enzymes (transferases), red font refers to catabolic enzymes (hydrolases). *—concerns chitosan only, ^—linkage via phosphodiester bond.

**Figure 3:**
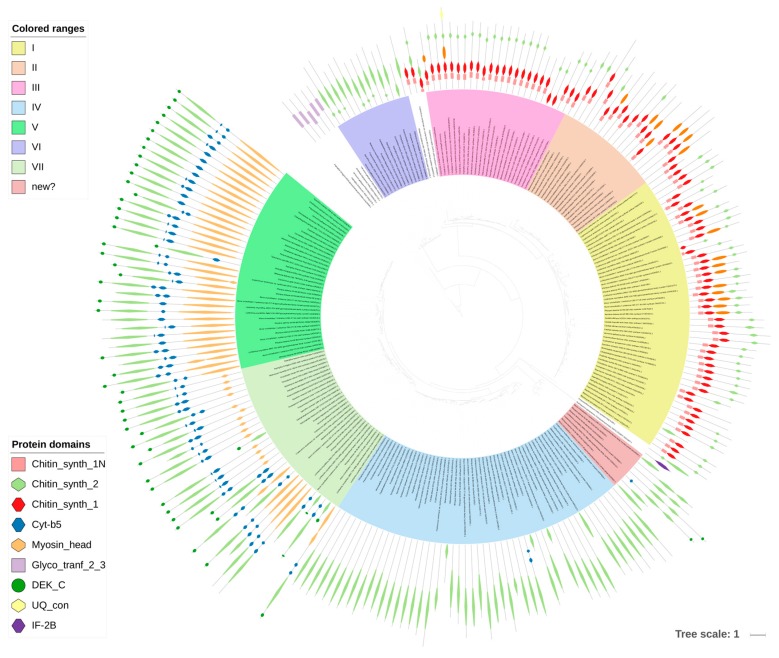
Phylogenetic tree of chitin synthases. The tree was built with PhyML (LG + G model with aLTR branch supports), the image was prepared with iTOL.

Chitin was historically considered to be the key to fungal identity, the fungal chemotaxonomic marker [7]. With the taxonomic turmoil regarding the kingdom Fungi, it is not that certain that chitin is the most obvious discriminant of fungal CWs, but it is still found to set up the innermost layer of the CW of most of the fungi studied to date. This compound is a linear polymer of β-1,4-linked *N*-acetylglucosamine (GlcNAc) folded into antiparallel chains, with hydrogen bonds stabilizing the whole molecule [25] (Figure 2C). It usually constitutes 5–10% of dry weight of the cell wall (all fungal CWs are made from more than 90% of polysaccharides), but it can reach up to 60% of polysaccharides in the cell wall in *Allomyces macrogynus* [26]. Chitin as one of the hardest molecules in nature, strongly determines the physical resistance of the fungal CW [25]. A proportion of chitin can be deacetylated after synthesis to form chitosan, which makes the polymer more elastic and resistant to chitinases [25]. In Mucoromycotina and *Cryptococcus neoformans*, more than two thirds of chitin is deacetylated to chitosan [25,26,27]. Consistently, our searches showed a huge abundance of chitin deacetylases (CE4) in Mucoromycotina representatives, which is consistent with previous findings and the fact that their cell wall is rich in chitosan [26], *Rhizopus oryzae* was previously reported to double the number of chitin- and chitosan-related genes [28]. Despite biochemical simplicity of the chitin molecule itself, its synthesis is a complex process requiring seven distinct classes of chitin synthases (CHS) grouped into three divisions based on their sequence and domain architecture (Figure 3). CHS classes are not uniformly distributed among fungal lineages, e.g., classes III, V, VI, and VII are absent in yeasts [25]. The deletion phenotypes for conserved CHS can vary between taxa. Our results show that two pathogenic taxa have gene duplications within specific CHS families, namely *Blastomyces dermatidis* (CHS III) and *Trichophyton rubrum* (CHS I). The three *Mucoromycotina* genomes have the highest number of CHS copies among all analyzed taxa. They possess multiple copies of CHS IV and V, and no copies of CHS II, III, and VI, confirming previous findings for *Phycomyces blakesleeanus* and *Rhizopus oryzae* [29]. A phylogenetic tree of all CHS identified in this study shows duplication characteristics, together with the domain architecture for particular taxa (Figure 3). Class V and VII proteins contain an additional myosin head motor domain, a fusion present in filamentous and basal fungi, but absent in yeasts. The myosin domain is important for exocytosis and transport of chitin synthase to the growing tip in filamentous fungi. James and Berbee [30] hypothesized that the presence of such fusions in Chytridiomycota and Rozella could be related to the need for polarized growth during host penetration. Interestingly, a case of convergent evolution of a similar fusion was found in animal evolution in the Mollusca and Annelida groups [31].

Obviously, the management of the CW chitin layer involves not only synthesis, but also degradation. The chitin layer structure can be altered by chitinases (GH18) and chitosanases (GH75). Chitinases can be both self and non-self degrading enzymes [32]. Chitosanases have a patchy distribution among the analyzed pathogenic taxa, with multiple copies in Eurotiales (*Aspergilli* and *Talaromyces*) and Sordariales (*Scedosporium* and *Purpureocillium*), and absent in all lineages other than filamentous Ascomycetes. The absence of chitosanases in taxa with high chitosan concentrations and presence thereof in mycoparastitic fungi may suggest they are mostly non-self degrading enzymes. GH18 chitinases are absent solely in the minimalist *Pneumocystis* genome, and are present in multiple copies in Mucoromycotina, Eurotiales, and Sordariales.

#### 3.2.2. Glucan

The key component of all fungal walls is glucan. Usually, it constitutes 50–60% of dry mass of the fungal CW [33]. The immunogenic β-1,3-glucan is often covered by other layers, preventing fungus from being recognized by the host defense. Since animals, in general, do not host fungal symbionts, β-1,3-glucan is one of the most universal fungal pathogen-associated molecular patterns (PAMPs) detected by dectin-1, a C-type lectin receptor of phagocytes [34]. Glucan is responsible for fungal activation of pro-inflammatory response by the innate immune system [1]. In order to evade immune response, fungi developed diverse camouflage methods. Some fungi, e.g., *Aspergillus fumigatus* and *Histoplasma capsulatum* [3,35], possess an additional, external α-1,3-glucan layer, to hide the innermost β-1,3-glucan [1] (Figure 2A). Other fungi, among others, *Cryptococcus neoformans* and *Malassezia restricta*, also have a significant fraction of β-1,6-glucan [36]. The covering layer can take the form of either a mucous structure (*C. neoformans*), or a shield of glycoproteins decorated with mannose, galactose, and other modifications [1,37]. The key enzyme involved in the assembly of the glucan polymer is the β-1,3-glucan synthase (GT48). Initially identified in *S. cerevisiae* as FKS protein, it was found to be universally conserved across fungi. The β-1,6-glucan synthase is yet to be described, but there are known proteins involved in regulation of synthesis and degradation of this compound [3]. The β-1,3-glucan structures need to be remodeled by diverse glucanases (GH131, GH132, GH81, GH12, GH64; Figure 2A) in the course of hyphal growth, budding, and spore forming. We demonstrate that many of the enzymes involved in glucan remodeling are absent in Mucoromycotina (GH132, GH131, GH30, GH13, GH55, GH71, GH1, GH64, and GT2), some are specific to Pezizomycotina (GH64, GH3, GH1), yet others are shared only by Basidiomycota and Pezizomycotina, but are absent in both analyzed *Candida* species (GH128, GH13, GH55, GH71). The putative TOS1-like glycosyl hydrolase (DUF2401) [38] is present only in Ascomycota, and is preceded by a glycine-rich protein domain (DUF2403) of unknown function. Yeast cell wall synthesis protein KRE9/KNH1 (KRE9, PF05390) homologs are present only in the analyzed Ascomycota, with a single expansion in *T. marneffei*. This protein has unknown molecular mechanisms of action, but is involved in cell wall β-1,6-glucan synthesis. Homologs of GPI-anchored were found to be important for CW assembly [39].

Within filamentous Ascomycota, there is a clear distinction between multiple expansions of the glucan machinery in Eurotiales and Sordariales, and low copy distribution of these enzymes in Onygenales.

#### 3.2.3. Glycosylation and Synthesis of Mannan

Yeast cells are often covered by an outermost layer of mannan chains, forming a network (Figure 2D). Their CW dry mass can contain up to 40–50% of mannan [40]. Mannans are mainly associated with proteins such as *O*- and *N*-linked glycans. Yeast wall mannoproteins often contain 50 to 95% carbohydrate by weight [41]. The *N*-linked glycans can be extended with an outer chain of 50 or more mannosyl residues [42]. Apart from mannose, mannoproteins in yeast contain also a minor amount of galactose, glucose, xylose, arabinose, fucose, and rhamnose. On the other hand, in several fungi, mannose is part of a complex of glycans, in addition to galactose, as in *Neurospora crassa* [43], or galactose and arabinose, as in the cell wall of *Botrytis cinerea* [44]. Large mannan chains (with more than 50 mannosyl residues) present in yeast have never been identified in filamentous fungi [45]. The average size of an oligosaccharide bound to glycoproteins in *A. fumigatus* ranges from 5 to 10 mannosyl residues linked to two *N*-acetylglucosamine residues [45].

These oligosaccharides are *N*-linked to the glycosylated proteins. Their synthesis starts in ER membrane, and involves subsequent addition of carbohydrate residues to lipid carrier dolichyl phosphate catalyzed by Asparagine-linked glycosylation (Alg) sugar transferases (GT1, GT4, GT2, GT22, GT33, GT57, GT58, GT59) [46] (Supplementary Figure S1). The resulting polysaccharide is transferred by oligosaccharyl transferase OST (Stt3p bearing catalytic site for OST complex, GT66) to target protein asparagine residues, characterized by the Asn–X–Ser/Thr sequences (Supplementary Figure S2) [47]. The core oligosaccharide is then processed in the endoplasmic reticulum (ER) by glycosidases that remove three glucosyl residues and one α-1,2-mannose unit, forming the core oligosaccharide characteristic for all eukaryotes. Further processing is initiated by Och1 mannosyltransferase (GT32), by attachment of the first α-1,6-mannosyl residue to all *N*-glycans [48]. Subsequent elaboration of the *N*-mannan chain is fungal specific. In *S. cerevisiae*, it is catalyzed by two complexes of mannanpolymerase I and II (GT62, GT71, GT34), and subsequently, by Mnn mannosyltransferases. In *C. albicans*, Mnn9 (GT62) is the major contributor to the extension of the α-1,6-mannosyl chain [46] (Supplementary Figure S3). *N*-linked mannans of *C. albicans* cell wall are potentially involved in virulence [49]. It was reported that *Candida* could have different structures of glycans, not only among species, but also between serotype of the same species [50].

Cell wall proteins are also decorated with mannosyl residues bound to serine or threonine hydroxyl group by *O*-glycosidic linkage, forming a short linear α-1,2/1,3-mannose chain (Supplementary Figure S4). The first mannosyl residue is attached to the protein by Pmt mannosyltransferase [51,52], and then other two mannosyl residues are transferred by Ktr1, Ktr2, and Ktr3 mannosyltransferases to form α-1,2-linkages [53,54]. Subsequently, Mnt1 and Mnt2 transferases extend the mannosyl chain by one or two α-1,3 linked mannoses. The *O*-linked glucan can be branched by addition of Man-P residue to the second α-1,2-mannose, by Mnt3 and Mnt5 mannosyltransferases having a redundant phosphomannosyltransferase activity [55]. It was shown that in *C. albicans*, *O*-glycosylation of the CWPs was important for adherence to host surfaces, and for virulence [56,57]. Mnt5 was suggested to also participate in the addition of a mannosyl residue to the *N*-glycan [55]. Members of the Ktr family contribute to *N*-linked carbohydrate chain synthesis, too [54].

The polysaccharides located in the outer part of the wall determine the serotype classification of *Candida albicans* [49]. Hydrophobic status of *C. albicans*, which is important for virulence, depends on β-1,2-oligomannoside chain lengths. It was shown that Mnt1 and Mnt2 (GH15) catalyze addition of mannosyl residues at the early step of polymannan assembly, and are essential for virulence or recognition of the fungus by macrophage [56]. In *A. fumigatus*, Mnt1 mannosyltransferase controls biosynthesis of mannan bound to β-glucan [6].

Glycoproteins are posttranslationally modified by addition of a glycosylphosphatidylinositol (GPI) anchor. GPI anchor is synthesized and attached to proteins in the ER (Supplementary Figure S1) [42,58]. Carbohydrate transferases, engaged in the synthesis of GPI precursor transfer GlcNAc residue to phosphatidylinositol forming α-1,6 linkage and then mannosyltransferases, add mannosyl residues, forming α-1,2, α-1,4 and α-1,6 linkages. In *A. fumigatus,* also, an α-1,3 linkage between two mannosyl residues was found in GPI membrane anchor-attached mannans [59]. After the protein is transferred to the ethanolamine group of GPI, GPI is remodeled mainly in its lipid part, and additional mannosyl residues are transferred by unknown mannosyltransferases to the four-mannose chain by creating α-1,2 or α-1,3 linkages [42]. Such modified CWP is finally transferred to the cell surface by the GPI anchor, released from the membrane, and displaced to the cell wall matrix [60]. To bind the GPI protein to the cell wall, GPI glucan is cleaved, possibly between glucosaminyl and mannosyl group, and its reducing end is transferred to β-1,6 glucan [42].

On the other hand, *N*-linked glycans of the CWP could be directly engaged in binding of the protein to the CW β-1,6-glucan [61]. A second class of proteins, which include the Pir-CWPs (protein with internal repeats), is directly linked to the CW β-1,3-glucan network through a mild alkali-sensitive linkage [62]. Pir-CWPs possess a number of repeats, allowing them to cross-link multiple β-1,3-glucan chains. This is consistent with their localization in the inner part of the cell wall [63,64].

The evolutionary conservation of the mannosylation pathways for different substrates is reflected in the low variation of taxonomic distribution of glycosylation and mannan related proteins in the analyzed taxa. Most enzymes are present in low copy number in all analyzed taxa with single cases of expansions, which renders mannosylation a core biological process. The experimentally observed differences in mannan presence in the CW are not easy to spot from the proteome perspective, and are likely an outcome of gene expression regulation, rather than of the presence of the enzymes themselves.

#### 3.2.4. Other Cell Wall Components

Lipids (including ceramides) constitute a minor fraction of CW, but perturbations in their synthesis can have a profound effect on CW integrity, e.g., in *Aspergilli*, they impact spore germination, cell cycle, and hyphal growth [65,66]. Glycosphingolipids are a combination of a sugar moiety covalently linked to a ceramide. Fungal glycosphingolipids differ from human ones, and thus, are considered promising targets for antifungal drugs [67,68]. Glycosphingolipids are important for fungal ability to colonize hosts. However, the exact molecular mechanism remains unknown, with one of the possible scenarios being transport across the cell wall of vesicles built from glycosphingolipids [67]. We found that ceramide glucosyl transferases (GT32 and GT21) are ubiquitous in human pathogenic fungi, except for *R. oryzae*. Enzymes involved in ceramide synthesis have expanded in *Purpureocillium lilacinum* that may be related to the elaboration of complex glucoceramides in this fungus, and its ability to colonize both insects and vertebrates.

Galactomannan is a mannose polymer with galactose side groups, characteristic for *Aspergillus*. Galactomannan is covalently bound to β-1,3-glucan-chitin core. *Aspergillus fumigatus* has galactosylated mannan, possibly assembled as a galactomannan-glycosyl phosphatidylinositol (GPI) anchor precursor [6]. Galactofuranose can be attached to *O*-glycosylated proteins via a specific β-1,6/1,5 UDP-galactofuranosyltransferase (GT31) [69,70]. GT31 homologs were identified only in filamentous fungi. The vegetative mycelium also contains galactosaminogalactan (GAG) composed of galactose, galactosamine, and *N*-acetylgalactosamine residues linked via α-1,4 linkage [71]. One of the galactomannoproteins are hydrophobic surface binding proteins A (HsbA, PF12296), which can be found in all analyzed taxonomic groups. The protein family evolution has been shaped by multiple duplications, leading to the formation of groups of species specific paralogs in Eurotiales and Mucoromycotina. Most of the HsbA proteins are single protein domain proteins known as antigenic cell wall galactommannoproteins [72].

Galactan is a polymer of galactose found in few Agaricomycetes (Inonotus, Pleurotus) [73]. Galactan can be reshaped by galactan 1,3-β-galactosidases (GH43), α-galactosidases (GH27), and α-1,4-galactosaminogalactan hydrolases (GH135), which seem to be limited to other than Onygenales filamentous Ascomycota, with the single exception of *Lichtheimia corymbifera* coding two α-galactosidases (GH27) and 3 α-1,2-galactosyltransferases (GT34). The α-1,2-galactosyltransferase (GT34) has a patchy distribution, with an expansion in *Exophiala oligosperma* and *Verruconis gallopava*, and absence in *Candida* and *Cryptococcus* species. Glucuronoxylomannan and galactoxylomannan β-1,2-xylosyltransferase are present in the proteomes of Pezizomycotina and *Cryptococcus*, with variable abundance, from a single copy in *Talaromyces marneffei* up to nine paralogs in *Purpureocillium lilacinum*.

Some carbohydrates are limited to few taxa. Fucose is a hexose found only in the CW of Mucoromycotina and single Basidiomycota representatives. The presence of fucose, and high chitosan and glucosamine levels, are considered the most important distinctive feature of the Mucoromycotina walls [26,29] as compared to Dikarya. α-Fucosyltransferases (GT10) are limited to Mucoromycotina and α-l-fucosidases (GH29) are additionally present in *P. liliacinum*. Scarce in fungi, fucose is a common sugar in animals, and can be used as an additional camouflage component.

Some CW-related transferases are documented only for *Cryptococcus*, but the predicted proteins are found in other taxa. Hyaluronan-like synthase (GT2) presence in all but *Candida*, *Pneumocystis* and *Malassezia* points at an understudied, but potentially significant factor contributing to fungal strategies of evading immune system, present already in Mucoromycotina. Hyaluronan constitutes one of the major components of the extracellular matrix in vertebrates. *N*-acetylglucosaminyl transferases (GT47 and GT49), involved in extracellular matrix homeostasis in animals, are also present in the proteomes of Mucoromycotina and *Cryptococcus* (only GT47). We hypothesize that GT49 transferase, present in 9–11 paralogs in the analyzed Mucoromycotina, might be β-1,3-glucuronosyltransferase (like animal homologs) involved in CW remodeling. Mucoromycotina have very high amounts of *N*-acetylglucosamine in their CW, and enzymes contributing to this excess have not been characterized. We hypothesize that GT47 and GT49 might contribute to the assembly of glycosaminoglycans, based on their function in animals (involvement in heparan sulfate and heparin biosynthesis).

Peptidorhamnomannans (PRMs) and rhamnomannans are found in the CW of *Scedosporium* and *Sporothrix* [74]. The activity of rhamnose synthase is required for virulence of plant pathogens *Verticillium* and *Botrytis* [75,76]. No rhamnosyltransferase has been characterized in fungi to date. Bacterial rhamnosyltransferases are grouped in Pfam RdpF (PF05045, Rhamnan synthesis protein F) which is related to GT2 family (FFAS score 13.100 between RdpF PF13641.4 and Glyco_tranf_2_3 PF05045.10). However, the 24 analyzed proteomes had no RdpF homologs, thus, a different rhamnosyltransferase should be present at least in the genomes of *Scedosporium* and *Sporothrix*.

Proteomic studies identified several proteins present in the carbohydrate fungal CW. Few of them are well characterized. Some are present in many taxa, such as Ecm33 and ROS protective proteins; others, like Pir repeat containing proteins (PIR, PF00399), are limited to Ascomycota. The aforementioned Ecm33 is the best studied family of structural CWPs ubiquitously present in all analyzed taxa. It contains lLeucine-rich repeats (LLR_5, PF13306) and helical motives similar to receptor L domain (Recep_L_domain, PF01030). Pfam Ecm33 (PF12454) profile is limited to *N*-terminal repeats characteristic for Pezizomycotina homologs.

Proteins involved in oxygen free radical detoxification are important for resistance against stress in their environment, which is especially beneficial for pathogens, since reactive oxygen species (ROS) are often a part of the host defense system. Laccases, which oxidize their substrate by accepting electrons at a copper center [77], are particularly abundant in Chaetothyriales, *Verruconis gallopava*, *Sporothrix schenckii*, and *Scedosporium apiospermum*. Superoxide dismutases were expanded in *Aspergillus*, *Exophiala oligosperma*, *Rhizopus oryzae*, and *Candida albicans*. *C. albicans* superoxide dismutase (SOD) copper–zinc dismutases SOD4, SOD5, and SOD6, are GPI anchored CWPs, potentially involved in stress resistance, neutralizing the superoxide (O_2_^−^) radical into either oxygen (O_2_) or hydrogen peroxide (H_2_O_2_) [78]. Catalases, which decompose hydrogen peroxide to water and oxygen, have been duplicated several times during fungal evolution, and subsequently, in the evolutionary lineages of e.g., Mucoromycotina and Onygenales, and remained in single copies only in *Candida*. All three ROS protecting enzymes seem to have been lost in *Pneumocystis jirovecii*.

### 3.3. Diverse Fungal Lineages

Related taxa tend to show similar proteome compositions, and this is also true for CWPs. All three analyzed Basidiomycotina have reduced, compact genomes with very limited abundance of both CWP families and representatives within each family.

Filamentous ascomycetes have a more complex landscape of CWP than yeast-like *Candida* and *Pneumocystis*. Three Mucorales have fewer CWP families than the average number, but more CWPs in total than the average for the analyzed pathogenic fungi. Structural, functional, and chemical differences in CW composition known from the best studied fungal pathogens [1] are also apparent, from the CWP protein perspective.

#### 3.3.1. The Minimalists: *Pneumocystis* and *Malassezia*

*Pneumocystis* and *Malassezia* genera have co-evolved with their mammalian hosts and reduced their genomes [79,80], with *Pneumocystis* being an extreme case of adaptation. Comparative genomics of 3 *Pneumocystis* species, confirmed by several assays, showed the loss of key components of the cell wall, including chitin synthesis pathway and outer chain *N*-mannans [80]. *Malassezia* species, on the other hand, possess a rigid CW with relatively high amounts of chitin and chitosan (up to 25% of the carbohydrate skeleton of the CW [36]), far more than other pathogenic basidiomycetes, e.g., *Cryptococcus neoformans* [81]). They also display significantly skewed proportions of glucans. *M. restricta* has 95% of glucan as β-1,6-glucan, and only 5% in the form of β-1,3-glucan [36].

Malassezia species are known for their reduced content of GPI-anchored proteins, the preference for chitosan over chitin itself, and glycosylation limited to *O*-glycosylation [82]. Moreover, the studies of *M. sympodiales* showed a lack of extensive outer fibrillar mannoprotein layer present in the wall of *S. cerevisiae* and *C. albicans* [82]. Our results confirm previous findings of an extreme simplification of CW-related protein repertoire, with the loss of chitin synthesis in *Pneumocystis* and reduction of CWP families in *Malassezia*. Interestingly, both taxa possess GT21 and GT32 ceramide glucosyltransferases potentially involved in immune mimicry.

#### 3.3.2. The Complex Eurotiales

*Aspergillus fumigatus* is the model filamentous fungus in CW studies. Its cell wall architecture changes during life cycle, and has been studied in detail with numerous knockout experiments validating functional predictions of genes coding CWP [6,83]. The core of its CW is comprised of β-1,3-glucan β-1,6-glucan and chitin, hyphal walls are covered by galactomannan and glycosaminoglycan (GAG), whereas conidia are shielded by a hydrophobin rodlet layer and melanin. A similar change in the pattern of CW-related genes during morphological switch was observed in *Talaromyces marneffei* [84]. The two analyzed *Aspergilli* and *T. marneffei* are the most complex in terms of total number of CWP families and number of CWP proteins. For instance, we have found that HsbA genes have been duplicated multiple times in the evolution of Eurotiales. A particularly numerous group of 13 paralogs is present in *T. marneffei* proteome, all forming a clade together with *Scedosporium apiospermum* protein HsbA representative (Supplementary Datasets S2 and S3). In *Aspergillus oryzae,* HsbA proteins are used to recruit lytic enzymes to the surface of hydrophobic solid materials and to promote their degradation [85]. Apart from being HsbA-rich, *T. marneffei* benefits also from expansion of GH18 chitinases as compared to both *Aspergillus* species. Chitinase expansions are documented in mycopathogenic taxa [32], but may also be involved in competition as well as in fast CW remodeling. Mycoparasites have more β-1,3/β-1,6-glucanases, needed for the degradation of glucan, than e.g., in fungicolous *Trichoderma* spp. [86]. In general, Eurotiales are characterized by a combination of several masking compounds, including α-1,3-glucan (GT5 α-1,3-glucan synthase, GH13 glucan branching enzyme, GH71 α-1,3-glucanase) and galactomannans (GT34 galactosyltransferase, GH43 galactan galactosidase, GH27 galactosidase, GH153 galactosaminogalactan hydrolase).

#### 3.3.3. Chromoblastomycosis Causing Fungi

Little is known about the CW of Chaetothyriales and their relatives, which have only recently been reported as opportunistic pathogens and linked with chromoblastomycosis (the latter is listed since 2017 as a neglected tropical disease by WHO). Comparative genome analyses revealed the decreased number of GH18 chitinases compared to Eurotiales and Onygenales, the lack of CH75 chitinases, and the loss of GT5 and GH13 of α-1,3-glucan synthases in several black yeast genomes [87]. The number of GH18 chitinases reported by Teixeira and colleagues [87], also recovered in our searches (3–6), is significantly lower than in other filamentous Ascomycetes analyzed, which have more than ten chitinases. Chitosanases (CH75) have been lost in several Sordariomycetes and *Candida*. Our results confirmed the loss of chitosanases (CH75) and chitosan exo-1,4-β-d-glucosaminidases (GH2) in Chaetothyriales. We were, however, able to detect Ags1-like α-1,3-glucan synthases (GT5) in *Cladophialophora bantiana*, *Exophiala oligosperma*, and *Rhinocladiella mackenziei* proteomes. We found that Chaetothyriales are particularly well equipped to deal with ROS stress with an elevated number of laccases, catalases, and dismutases. Interestingly, *Exophiala oligosperma* may contain complex α-galactose-containing glycans in the CW, due to expansion of α-1,2-galactosyltransferases from GT34 family. Such α-galactose-containing glycans are present on the cell surface of *Schizosaccharomyces pombe* [88]. Chaetothyriales possess a unique domain architecture of β-glucosidases (GH3) with terminal Pir repeats possibly linking the protein to the CW. Additionally, their cell wall might be dynamically reshaped by numerous GH16 β-1,3-glucan glucanases.

#### 3.3.4. Animal-Related Onygenales

The dimorphic and dermatophytic fungi grouped in Onygenales are descendants of plant-associated ancestors [89]. The composition of CW of *Blastomyces dermatidis* [90], *Paracoccidioides brasiliensis* [91], *Histoplasma capsulatum* (*Ajellomyces capsulatus*) [92], *Coccidioides immitis* [93] was established in the 1970s. Their cell wall composition depends on both morphological form and the cultivation media. Our results show a reduction of CWPs in animal-related Onygenales, as compared to their sister group Eurotiales, ancestrally related to plant hosts. Histoplasma and Blastomyces are known to have an outer α-1,3-glucan layer to evade the immune system recognizing the β-1,3-glucan [1]. Our searches confirmed the presence of Ags-like α-1,3-glucan synthase in all Onygenales, excluding *Trichophyton rubrum*. Dermatophytic *T. rubrum* significantly differs in CWP composition from the dimorphic relatives. It has specific expansions of chitin synthases (GT2), chitinases (GH18), β-1,3 glucan synthases (GT48), β-1,6 glucosyltransferases (GT24). And what is extraordinary for animal-related onygenales, retains chitooligosaccharide oxygenase (AA7), chitosanases (GH75), and chitosan exo-1,4-β-d-glucosaminidase (GH2). Therefore, *T. rubrum* has a more dynamic CW, possibly devoid of β-1,4-branches and α-1,3-glucan, due to lack of mixed β-1,3/1,4 (GT2) and α-1,3/α-1,4-glucan synthases (GH13), and ability to degrade competitors’ cell wall.

#### 3.3.5. Opportunistic Sordariales

*Purpureocillium lilacinum* and *Scedosporium apiospermum* cause rare opportunistic infections, and have been just recently described as emerging pathogens resistant to many antifungal drugs [94]. The former one is an entomopathogen, while the latter is commonly found in environmental samples [94]. As a consequence, previous research was focused on aspects of their biology different than in human infections. Specifically, little is known on the CW composition of the analyzed Sordariales. *Sporothrix schenckii*, a causal agent of feline and human skin infections [95], is slightly better investigated. It is a melanized fungus with β-1,3-glucan and β-1,6-glucan components, however, genes responsible for synthesis of either β-1,6- or β-1,4 bonds were unknown [95]. We were able to identify a mixed glucan synthase homologous to CelA (GT2), and six GT90 glucuronoxylomannan/galactoxylomannan β-1,2-xylosyltransferases. *P. lilacinum* has impressive expansions of several CW-associated proteins, among others, ten ceramide glucosyltransferases (GT32), ten GXM/GalXM transferases (GT90), and 34 chitinases (GH18). The abundance of chitinases might be related to the primary entomopathogenic lifestyle of *P. liliacinum* and chitin cuticle degradation. *S. apiospermum*, on the other hand, has an average CWP composition, lacks specific expansions, except for GH3 β-glucosidases, and has no GXM/GalXM transferases, which corroborates the hypothesis that decorative CW elements have a patchy taxonomic distribution.

#### 3.3.6. Commensal and Pathogenic—*Candida*

*Candida* species are covered by a mannan layer that hides the immunogenic glucan. CWPs in *Candida* include enzymes known to be involved in cell wall remodeling, but also a growing number of moonlighting proteins with their primary function localized to cytoplasm [96]. Cell wall enzymes play a role in degradation of large impermeable molecules, making the products accessible for cell nutrition; others are involved in degradation of CW polymers or in their synthesis, being necessary for wall, and therefore, for cell growth [49]. The overall composition of CWPs in the two analyzed *Candida* species is highly similar, *Candida tropicalis* having only a lower number of both SOD copper zinc dismutases [78]) (two, as compared to four in *C. albicans* SOD1, SOD4, SOD5, and SOD6) and mannosyltransferases from families GT62, GT15, GT71, GT91.

#### 3.3.7. *Cryptococcus* and the Capsule

*Cryptococcus* cell wall is unique in terms of its outer mucous layer (capsule) decorated with special compounds masking the immunogenic glucan inner layer [37]. The capsule is composed in 90% of glucuronoxylomannan (GXM) and galactoxylomannan (GalXM), with a minor fraction of mannoproteins [97]. Its CW, like one of the *Aspergillus* and dimorphic fungi, has a α-1,3-glucan fraction involved in association of capsule material with the cell surface [98]. Interestingly, *Cryptococcus* CW has more β-1,6-glucan than β-1,3-glucan. The overall CWP repertoire of *Cryptococcus* is relatively small and reduced as compared to filamentous ascomycetes, but it shows signs of high specialization, with additional enzymes apart from the classical core machinery (involved in chitin and glucan metabolism) for synthesis of hyaluronic acid (GT2), glucuronoxylomannan (GXM), and galactoxylomannan (GalXM) (GT90). Two analyzed *Cryptococcus* species lack several glycosylation enzymes, e.g., α-mannosyltransferases (GH62), Dol-P-Glc: α-1,3-glucosyltransferases (GT57), α-1,2-glucosidase I (GH63), and glucan-related mixed glucanase (GH131, GH12), mixed glucan synthase CelA (GT2), β-glucosidase (GH1), β-1,3-glucanases (GH55, GH64), chitooligossacharide oxygenases (AA7), and chitinases (GH75). *C. gattii* has five SOD dismutases, as compared to only two in *C. neoformans*, which might contribute to *C. gattii* enhanced ROS resistance.

#### 3.3.8. Mucoromycotina

The cell wall in Mucorales is rich in chitosan and *N*-acetylglucosamine, decorated with fucose, a carbohydrate present only in few fungal taxa [26,29]. They have a relatively high amount of CWPs belonging to few CWP families, with *Lichtheimia corymbifera* possessing the most complex CWP landscape and *Rhizopus oryzae* having representatives of only 43 out of 78 CWP families. *L. corymbifera* has fewer chitin deacetylases, chitinases, and chitin synthases, but more β-1,6-glucosyltransferases (GT24) and β-1,3-*N*-acetylglucosaminyltransferases (GT49) than *M. circinelloides* and *R. oryzae*. Additionally, *L. corymbifera* retains GPI anchor synthesis (GT69, GT76) and ceramide biosynthesis (GH5, GT21) enzymes absent in *R. oryzae*. Analyzed Mucoromycotina are distinguished from Dikarya by the presence of GT10 α-fucosyltransferases, and GT47 and GT49 *N*-acetylglucosaminyl transferases. They possess an expansion of GDP-Man: α-1,2-mannosyltransferases (GT15), chitin synthases (GT2), chitinases (GH75), and chitin deacetylases (CE4). The observed chitin-related enzyme expansion was previously reported for *R. oryzae* and *Phycomyces blakeleenus* [29]. Mucoromycotina CW seems to be significantly different from Dikarya, which can explain unsuccessful treatment.

## 4. Conclusions

The aim of this project was to explore the composition of cell wall related proteins within diverse specialized and opportunistic fungal pathogens. We identified and analyzed the distribution of the key components of the cell wall, and found common themes in fungal pathogens, as well as peculiarities characteristic for each fungus. Our results significantly expand the ensemble of CW-related proteins in emerging human pathogens, and demonstrate parallel adaptations in distinct pathogenic taxa. By systematic identification of multiple CW protein modeling, we showed that many experimentally determined CW characteristics are reflected in the encoded gene repertoire. Based on that, the obtained results might allow for prediction of CW characteristics in understudied species, and eventually guide further rational design of experimental research, aiming to explore interesting CW aspects in a more detailed manner.

## Supplementary Materials

The following are available online at www.mdpi.com/2309-608X/4/1/6/s1. Table S1. (A) The list of genomes downloaded from NCBI genomes in July 2017 with corresponding literature references; (B) Cell wall related proteins with CaZy and Pfam identifiers; (C) Distribution and abundance of each CWP family in the 24 proteomes analyzed. Figure S1. Synthesis of GPI anchor in the ER; Figure S2. *N*-glycosylation schema; Figure S3. Further steps of *N*-glycosylation and mannan formation; Figure S4. *O*-mannosylation schema. Dataset S1. All sequence identifiers; Dataset S2. All phylogenetic trees in newick; Dataset S3. All phylogenetic trees in pdf formats.
