# Supplementary material for: Diversity of Cell Wall Related Proteins in Human Pathogenic Fungi
Source: J Fungi (Basel). 2017 Dec 29;4(1):6. doi: 10.3390/jof4010006 (PMC5872309; doi:10.3390/jof4010006)
Supplement: Supplementary file 1 [file jof-04-00006-s001.zip › jof-252817-final-supplementary materials/Dataset S3_trees_pdf/ALG14.pdf]

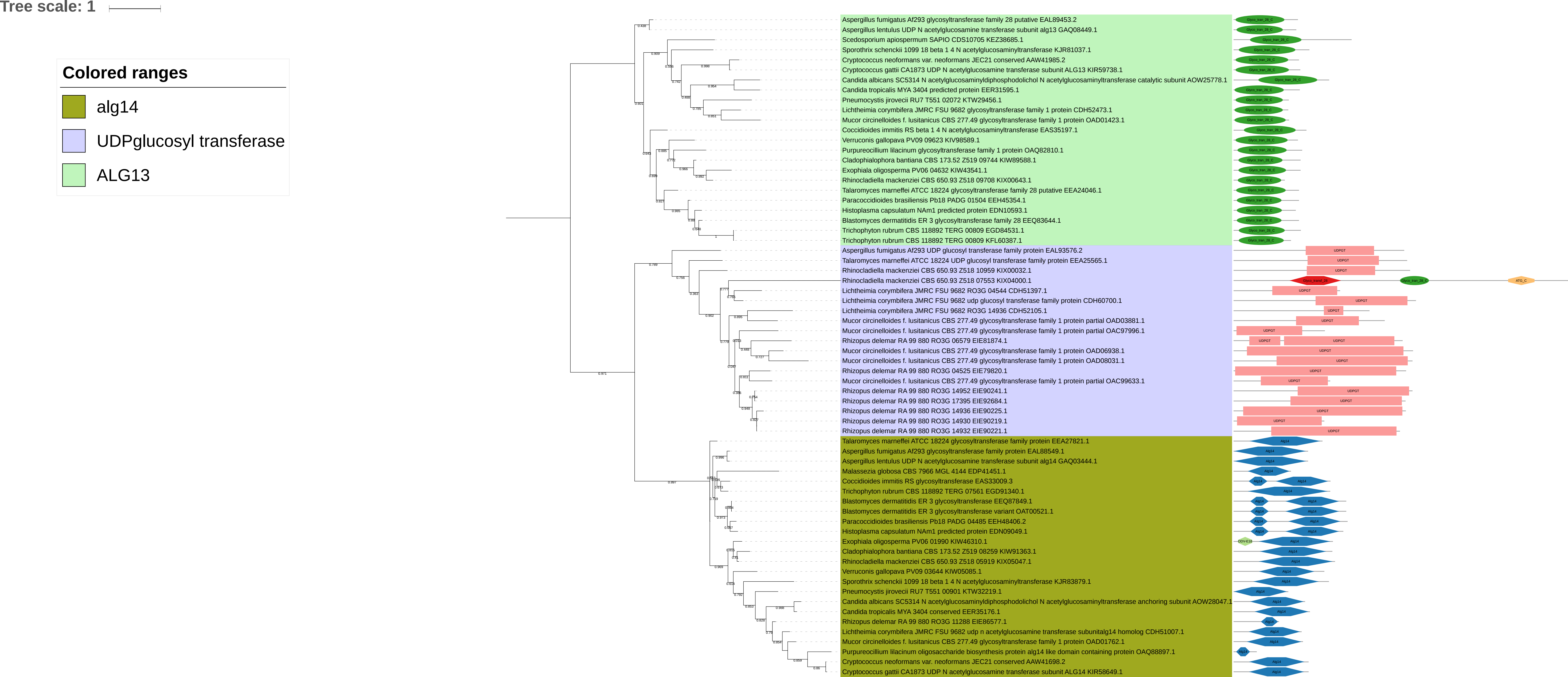
Tree scale: 1
Colored ranges
alg14
UDPglucosyl transferase
ALG13
Aspergillus fumigatus Af293 glycosyltransferase family 28 putative EAL89453.2
Aspergillus lentulus UDP N acetylglucosamine transferase subunit alg13 GAQ08449.1
Scedosporium apiospermum SAPIO CDS10705 KEZ38685.1
Sporothrix schenckii 1099 18 beta 1 4 N acetylglucosaminyltransferase KJR81037.1
Cryptococcus neoformans var. neoformans JEC21 conserved AAW41985.2
Cryptococcus gattii CA1873 UDP N acetylglucosamine transferase subunit ALG13 KIR59738.1
Candida albicans SC5314 N acetylglucosaminyldiphosphodolichol N acetylglucosaminyltransferase catalytic subunit AOW25778.1
Candida tropicalis MYA 3404 predicted protein EER31595.1
Pneumocystis jirovecii RU7 T551 02072 KTW29456.1
Lichtheimia corymbifera JMRC FSU 9682 glycosyltransferase family 1 protein CDH52473.1
Mucor circinelloides f. lusitanicus CBS 277.49 glycosyltransferase family 1 protein OAD01423.1
Coccidioides immitis RS beta 1 4 N acetylglucosaminyltransferase EAS35197.1
Verruconis gallopava PV09 09623 KIV98589.1
Purpureocillium lilacinum glycosyltransferase family 1 protein OAQ82810.1
Cladophialophora bantiana CBS 173.52 Z519 09744 KIW89588.1
Exophiala oligosperma PV06 04632 KIW43541.1
Rhinocladiella mackenziei CBS 650.93 Z518 09708 KIX00643.1
Talaromyces marneffei ATCC 18224 glycosyltransferase family 28 putative EEA24046.1
Paracoccidioides brasiliensis Pb18 PADG 01504 EEH45354.1
Histoplasma capsulatum NAm1 predicted protein EDN10593.1
Blastomyces dermatitidis ER 3 glycosyltransferase family 28 EEQ83644.1
Trichophyton rubrum CBS 118892 TERG 00809 EGD84531.1
Trichophyton rubrum CBS 118892 TERG 00809 KFL60387.1
Aspergillus fumigatus Af293 UDP glucosyl transferase family protein EAL93576.2
Talaromyces marneffei ATCC 18224 UDP glucosyl transferase family protein EEA25565.1
Rhinocladiella mackenziei CBS 650.93 Z518 10959 KIX00032.1
Rhinocladiella mackenziei CBS 650.93 Z518 07553 KIX04000.1
Lichtheimia corymbifera JMRC FSU 9682 RO3G 04544 CDH51397.1
Lichtheimia corymbifera JMRC FSU 9682 udp glucosyl transferase family protein CDH60700.1
Lichtheimia corymbifera JMRC FSU 9682 RO3G 14936 CDH52105.1
Mucor circinelloides f. lusitanicus CBS 277.49 glycosyltransferase family 1 protein partial OAD03881.1
Mucor circinelloides f. lusitanicus CBS 277.49 glycosyltransferase family 1 protein partial OAC97996.1
Rhizopus delemar RA 99 880 RO3G 06579 EIE81874.1
Mucor circinelloides f. lusitanicus CBS 277.49 glycosyltransferase family 1 protein OAD06938.1
Mucor circinelloides f. lusitanicus CBS 277.49 glycosyltransferase family 1 protein OAD08031.1
Rhizopus delemar RA 99 880 RO3G 04525 EIE79820.1
Mucor circinelloides f. lusitanicus CBS 277.49 glycosyltransferase family 1 protein partial OAC99633.1
Rhizopus delemar RA 99 880 RO3G 14952 EIE90241.1
Rhizopus delemar RA 99 880 RO3G 17395 EIE92684.1
Rhizopus delemar RA 99 880 RO3G 14936 EIE90225.1
Rhizopus delemar RA 99 880 RO3G 14930 EIE90219.1
Rhizopus delemar RA 99 880 RO3G 14932 EIE90221.1
Talaromyces marneffei ATCC 18224 glycosyltransferase family protein EEA27821.1
Aspergillus fumigatus Af293 glycosyltransferase family protein EAL88549.1
Aspergillus lentulus UDP N acetylglucosamine transferase subunit alg14 GAQ03444.1
Malassezia globosa CBS 7966 MGL 4144 EDP41451.1
Coccidioides immitis RS glycosyltransferase EAS33009.3
Trichophyton rubrum CBS 118892 TERG 07561 EGD91340.1
Blastomyces dermatitidis ER 3 glycosyltransferase EEQ87849.1
Blastomyces dermatitidis ER 3 glycosyltransferase variant OAT00521.1
Paracoccidioides brasiliensis Pb18 PADG 04485 EEH48406.2
Histoplasma capsulatum NAm1 predicted protein EDN09049.1
Exophiala oligosperma PV06 01990 KIW46310.1
Cladophialophora bantiana CBS 173.52 Z519 08259 KIW91363.1
Rhinocladiella mackenziei CBS 650.93 Z518 05919 KIX05047.1
Verruconis gallopava PV09 03644 KIW05085.1
Sporothrix schenckii 1099 18 beta 1 4 N acetylglucosaminyltransferase KJR83879.1
Pneumocystis jirovecii RU7 T551 00901 KTW32219.1
Candida albicans SC5314 N acetylglucosaminyldiphosphodolichol N acetylglucosaminyltransferase anchoring subunit AOW28047.1
Candida tropicalis MYA 3404 conserved EER35176.1
Rhizopus delemar RA 99 880 RO3G 11288 EIE86577.1
Lichtheimia corymbifera JMRC FSU 9682 udp n acetylglucosamine transferase subunitalg14 homolog CDH51007.1
Mucor circinelloides f. lusitanicus CBS 277.49 glycosyltransferase family 1 protein OAD01762.1
Purpureocillium lilacinum oligosaccharide biosynthesis protein alg14 like domain containing protein OAQ88897.1
Cryptococcus neoformans var. neoformans JEC21 conserved AAW41698.2
Cryptococcus gattii CA1873 UDP N acetylglucosamine transferase subunit ALG14 KIR58649.1
