# Supplementary material for: Diversity of Cell Wall Related Proteins in Human Pathogenic Fungi
Source: J Fungi (Basel). 2017 Dec 29;4(1):6. doi: 10.3390/jof4010006 (PMC5872309; doi:10.3390/jof4010006)
Supplement: Supplementary file 1 [file jof-04-00006-s001.zip › jof-252817-final-supplementary materials/Dataset S3_trees_pdf/Catalase.pdf]

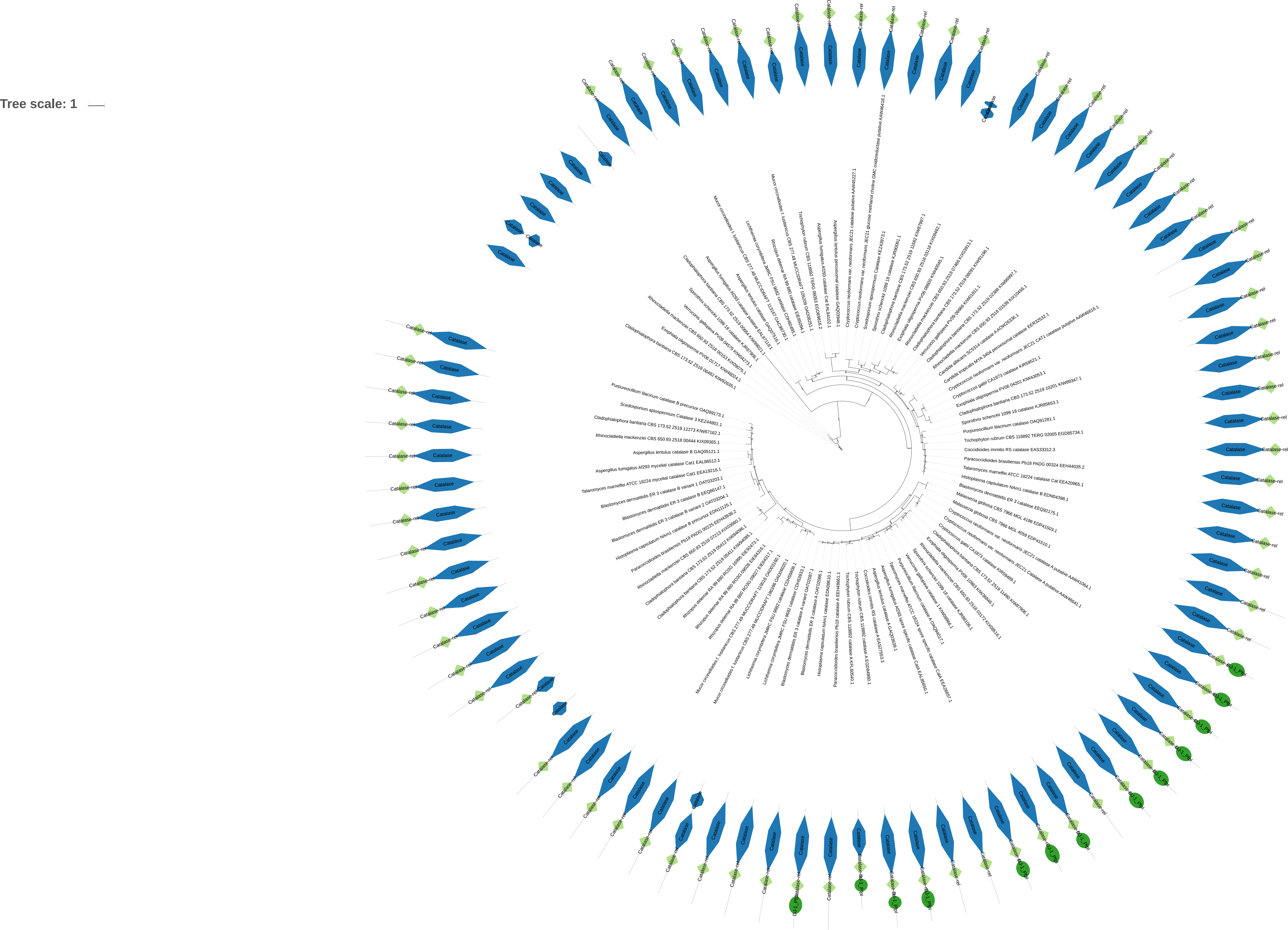

Tree scale: 1
Catalase
Catalase-rel
