# Supplementary material for: Diversity of Cell Wall Related Proteins in Human Pathogenic Fungi
Source: J Fungi (Basel). 2017 Dec 29;4(1):6. doi: 10.3390/jof4010006 (PMC5872309; doi:10.3390/jof4010006)
Supplement: Supplementary file 1 [file jof-04-00006-s001.zip › jof-252817-final-supplementary materials/Dataset S3_trees_pdf/CE9.pdf]

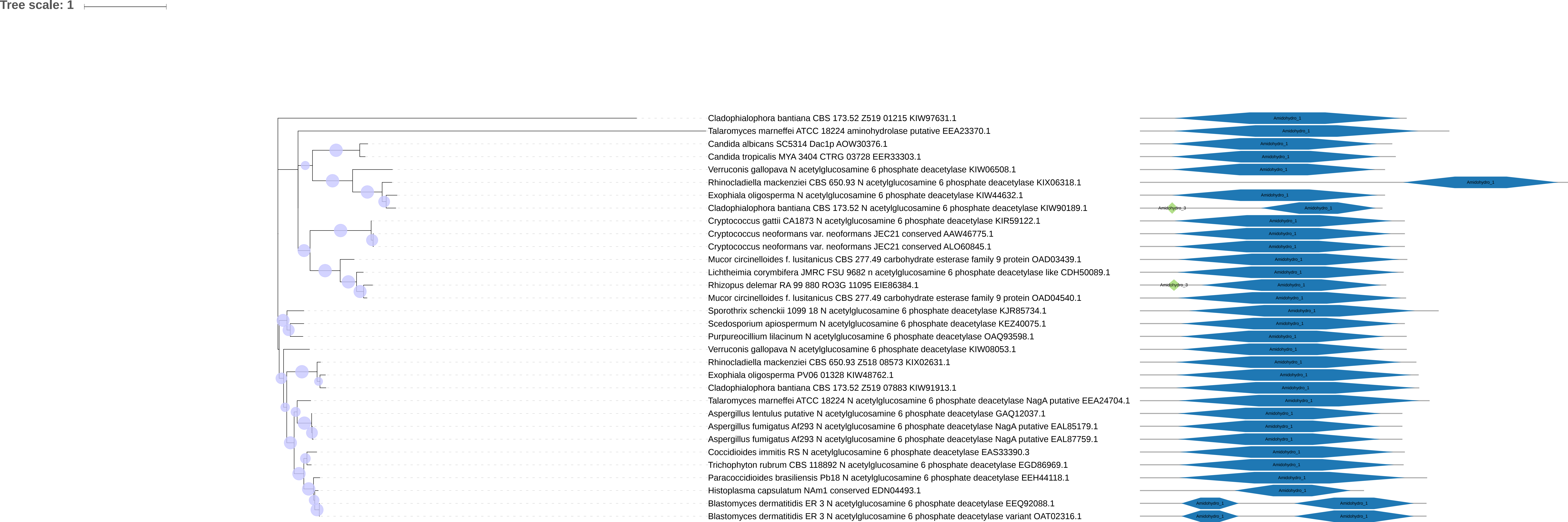

Tree scale: 1
Cladophialophora bantiana CBS 173.52 Z519 01215 KIW97631.1
Talaromyces marneffei ATCC 18224 aminohydrolase putative EEA23370.1
Candida albicans SC5314 Dac1p AOW30376.1
Candida tropicalis MYA 3404 CTRG 03728 EER33303.1
Verruconis gallopava N acetylglucosamine 6 phosphate deacetylase KIW06508.1
Rhinocladiella mackenziei CBS 650.93 N acetylglucosamine 6 phosphate deacetylase KIX06318.1
Exophiala oligosperma N acetylglucosamine 6 phosphate deacetylase KIW44632.1
Cladophialophora bantiana CBS 173.52 N acetylglucosamine 6 phosphate deacetylase KIW90189.1
Cryptococcus gattii CA1873 N acetylglucosamine 6 phosphate deacetylase KIR59122.1
Cryptococcus neoformans var. neoformans JEC21 conserved AAW46775.1
Cryptococcus neoformans var. neoformans JEC21 conserved ALO60845.1
Mucor circinelloides f. lusitanicus CBS 277.49 carbohydrate esterase family 9 protein OAD03439.1
Lichtheimia corymbifera JMRC FSU 9682 n acetylglucosamine 6 phosphate deacetylase like CDH50089.1
Rhizopus delemar RA 99 880 RO3G 11095 EIE86384.1
Mucor circinelloides f. lusitanicus CBS 277.49 carbohydrate esterase family 9 protein OAD04540.1
Sporothrix schenckii 1099 18 N acetylglucosamine 6 phosphate deacetylase KJR85734.1
Scedosporium apiospermum N acetylglucosamine 6 phosphate deacetylase KEZ40075.1
Purpureocillium lilacinum N acetylglucosamine 6 phosphate deacetylase OAQ93598.1
Verruconis gallopava N acetylglucosamine 6 phosphate deacetylase KIW08053.1
Rhinocladiella mackenziei CBS 650.93 Z518 08573 KIX02631.1
Exophiala oligosperma PV06 01328 KIW48762.1
Cladophialophora bantiana CBS 173.52 Z519 07883 KIW91913.1
Talaromyces marneffei ATCC 18224 N acetylglucosamine 6 phosphate deacetylase NagA putative EEA24704.1
Aspergillus lentulus putative N acetylglucosamine 6 phosphate deacetylase GAQ12037.1
Aspergillus fumigatus Af293 N acetylglucosamine 6 phosphate deacetylase NagA putative EAL85179.1
Aspergillus fumigatus Af293 N acetylglucosamine 6 phosphate deacetylase NagA putative EAL87759.1
Coccidioides immitis RS N acetylglucosamine 6 phosphate deacetylase EAS33390.3
Trichophyton rubrum CBS 118892 N acetylglucosamine 6 phosphate deacetylase EGD86969.1
Paracoccidioides brasiliensis Pb18 N acetylglucosamine 6 phosphate deacetylase EEH44118.1
Histoplasma capsulatum NAm1 conserved EDN04493.1
Blastomyces dermatitidis ER 3 N acetylglucosamine 6 phosphate deacetylase EEQ92088.1
Blastomyces dermatitidis ER 3 N acetylglucosamine 6 phosphate deacetylase variant OAT02316.1
