# Supplementary material for: Diversity of Cell Wall Related Proteins in Human Pathogenic Fungi
Source: J Fungi (Basel). 2017 Dec 29;4(1):6. doi: 10.3390/jof4010006 (PMC5872309; doi:10.3390/jof4010006)
Supplement: Supplementary file 1 [file jof-04-00006-s001.zip › jof-252817-final-supplementary materials/Dataset S3_trees_pdf/Ecm33.pdf]

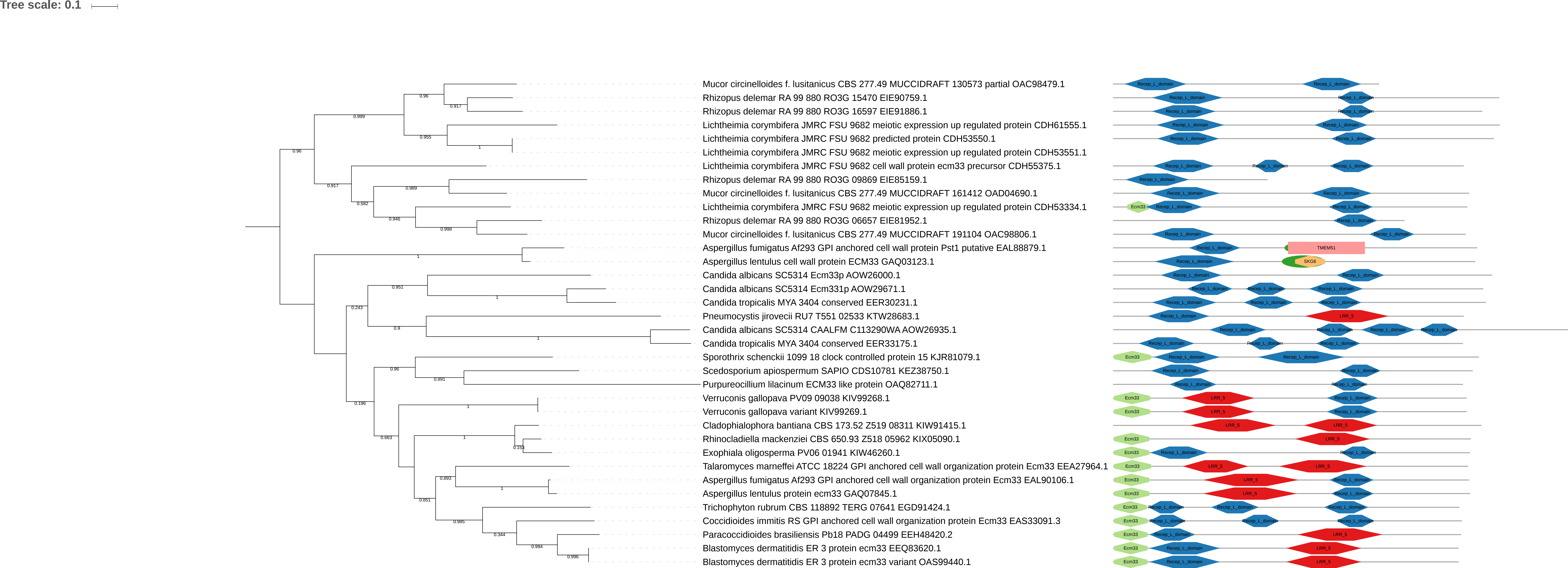
Tree scale: 0.1
Mucor circinelloides f. lusitanicus CBS 277.49 MUCCIDRAFT 130573 partial OAC98479.1
Rhizopus delemar RA 99 880 RO3G 15470 EIE90759.1
Rhizopus delemar RA 99 880 RO3G 16597 EIE91886.1
Lichtheimia corymbifera JMRC FSU 9682 meiotic expression up regulated protein CDH61555.1
Lichtheimia corymbifera JMRC FSU 9682 predicted protein CDH53550.1
Lichtheimia corymbifera JMRC FSU 9682 meiotic expression up regulated protein CDH53551.1
Lichtheimia corymbifera JMRC FSU 9682 cell wall protein ecm33 precursor CDH55375.1
Rhizopus delemar RA 99 880 RO3G 09869 EIE85159.1
Mucor circinelloides f. lusitanicus CBS 277.49 MUCCIDRAFT 161412 OAD04690.1
Lichtheimia corymbifera JMRC FSU 9682 meiotic expression up regulated protein CDH53334.1
Rhizopus delemar RA 99 880 RO3G 06657 EIE81952.1
Mucor circinelloides f. lusitanicus CBS 277.49 MUCCIDRAFT 191104 OAC98806.1
Aspergillus fumigatus Af293 GPI anchored cell wall protein Pst1 putative EAL88879.1
Aspergillus lentulus cell wall protein ECM33 GAQ03123.1
Candida albicans SC5314 Ecm33p AOW26000.1
Candida albicans SC5314 Ecm331p AOW29671.1
Candida tropicalis MYA 3404 conserved EER30231.1
Pneumocystis jirovecii RU7 T551 02533 KTW28683.1
Candida albicans SC5314 CAALFM C113290WA AOW26935.1
Candida tropicalis MYA 3404 conserved EER33175.1
Sporothrix schenckii 1099 18 clock controlled protein 15 KJR81079.1
Scedosporium apiospermum SAPIO CDS10781 KEZ38750.1
Purpureocillium lilacinum ECM33 like protein OAQ82711.1
Verruconis gallopava PV09 09038 KIV99268.1
Verruconis gallopava variant KIV99269.1
Cladophialophora bantiana CBS 173.52 Z519 08311 KIW91415.1
Rhinocladiella mackenziei CBS 650.93 Z518 05962 KIX05090.1
Exophiala oligosperma PV06 01941 KIW46260.1
Talaromyces marneffei ATCC 18224 GPI anchored cell wall organization protein Ecm33 EEA27964.1
Aspergillus fumigatus Af293 GPI anchored cell wall organization protein Ecm33 EAL90106.1
Aspergillus lentulus protein ecm33 GAQ07845.1
Trichophyton rubrum CBS 118892 TERG 07641 EGD91424.1
Coccidioides immitis RS GPI anchored cell wall organization protein Ecm33 EAS33091.3
Paracoccidioides brasiliensis Pb18 PADG 04499 EEH48420.2
Blastomyces dermatitidis ER 3 protein ecm33 EEQ83620.1
Blastomyces dermatitidis ER 3 protein ecm33 variant OAS99440.1
Recep_L_domain
Ecm33
LRR_5
TMEM51
SKG6
