# Supplementary material for: Diversity of Cell Wall Related Proteins in Human Pathogenic Fungi
Source: J Fungi (Basel). 2017 Dec 29;4(1):6. doi: 10.3390/jof4010006 (PMC5872309; doi:10.3390/jof4010006)
Supplement: Supplementary file 1 [file jof-04-00006-s001.zip › jof-252817-final-supplementary materials/Dataset S3_trees_pdf/GH1.pdf]

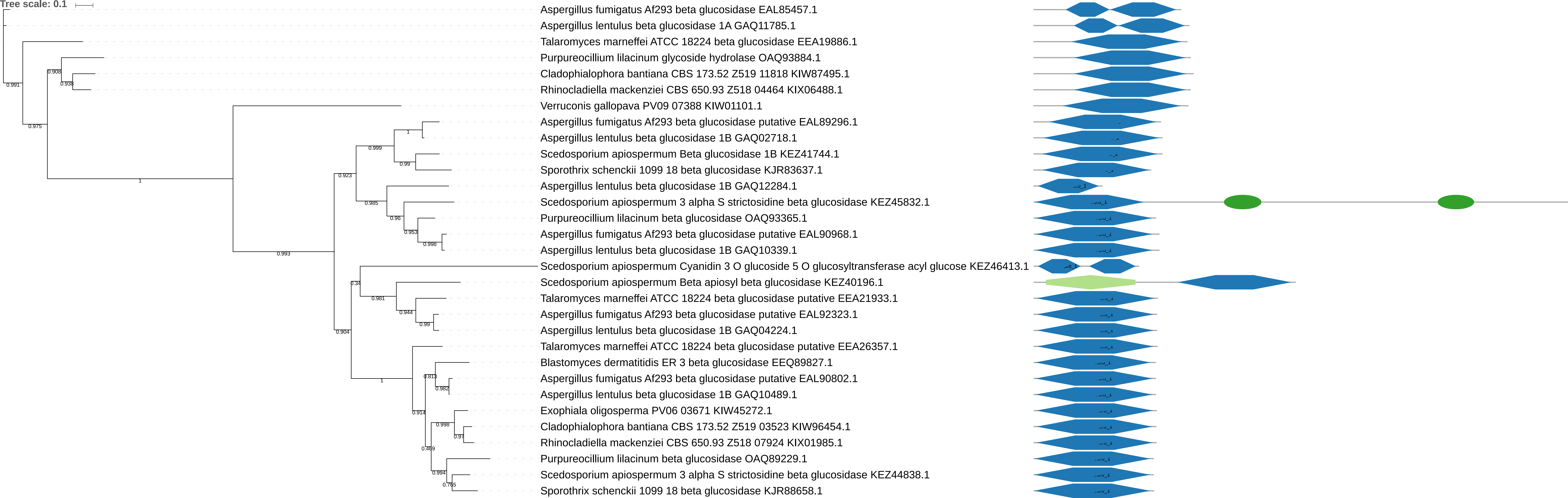

Tree scale: 0.1
Aspergillus fumigatus Af293 beta glucosidase EAL85457.1
Aspergillus lentulus beta glucosidase 1A GAQ11785.1
Talaromyces marneffei ATCC 18224 beta glucosidase EEA19886.1
Purpureocillium lilacinum glycoside hydrolase OAQ93884.1
Cladophialophora bantiana CBS 173.52 Z519 11818 KIW87495.1
Rhinocladiella mackenziei CBS 650.93 Z518 04464 KIX06488.1
Verruconis gallopava PV09 07388 KIW01101.1
Aspergillus fumigatus Af293 beta glucosidase putative EAL89296.1
Aspergillus lentulus beta glucosidase 1B GAQ02718.1
Scedosporium apiospermum Beta glucosidase 1B KEZ41744.1
Sporothrix schenckii 1099 18 beta glucosidase KJR83637.1
Aspergillus lentulus beta glucosidase 1B GAQ12284.1
Scedosporium apiospermum 3 alpha S strictosidine beta glucosidase KEZ45832.1
Purpureocillium lilacinum beta glucosidase OAQ93365.1
Aspergillus fumigatus Af293 beta glucosidase putative EAL90968.1
Aspergillus lentulus beta glucosidase 1B GAQ10339.1
Scedosporium apiospermum Cyanidin 3 O glucoside 5 O glucosyltransferase acyl glucose KEZ46413.1
Scedosporium apiospermum Beta apiosyl beta glucosidase KEZ40196.1
Talaromyces marneffei ATCC 18224 beta glucosidase putative EEA21933.1
Aspergillus fumigatus Af293 beta glucosidase putative EAL92323.1
Aspergillus lentulus beta glucosidase 1B GAQ04224.1
Talaromyces marneffei ATCC 18224 beta glucosidase putative EEA26357.1
Blastomyces dermatitidis ER 3 beta glucosidase EEQ89827.1
Aspergillus fumigatus Af293 beta glucosidase putative EAL90802.1
Aspergillus lentulus beta glucosidase 1B GAQ10489.1
Exophiala oligosperma PV06 03671 KIW45272.1
Cladophialophora bantiana CBS 173.52 Z519 03523 KIW96454.1
Rhinocladiella mackenziei CBS 650.93 Z518 07924 KIX01985.1
Purpureocillium lilacinum beta glucosidase OAQ89229.1
Scedosporium apiospermum 3 alpha S strictosidine beta glucosidase KEZ44838.1
Sporothrix schenckii 1099 18 beta glucosidase KJR88658.1
