# Supplementary material for: Diversity of Cell Wall Related Proteins in Human Pathogenic Fungi
Source: J Fungi (Basel). 2017 Dec 29;4(1):6. doi: 10.3390/jof4010006 (PMC5872309; doi:10.3390/jof4010006)
Supplement: Supplementary file 1 [file jof-04-00006-s001.zip › jof-252817-final-supplementary materials/Dataset S3_trees_pdf/GH12.pdf]

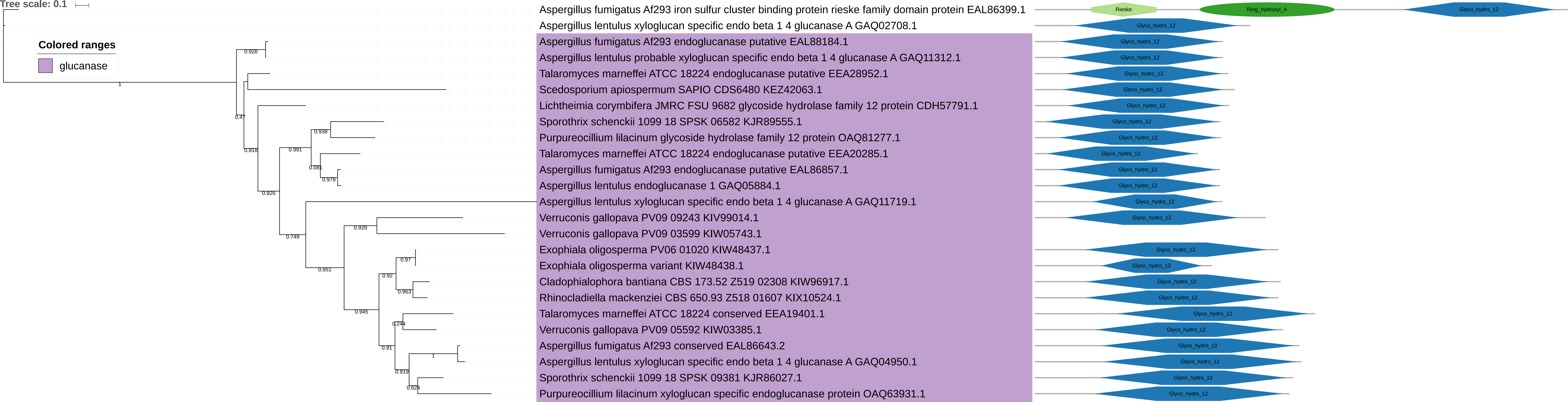
Tree scale: 0.1
Colored ranges
glucanase
Aspergillus fumigatus Af293 iron sulfur cluster binding protein rieske family domain protein EAL86399.1
Aspergillus lentulus xyloglucan specific endo beta 1 4 glucanase A GAQ02708.1
Aspergillus fumigatus Af293 endoglucanase putative EAL88184.1
Aspergillus lentulus probable xyloglucan specific endo beta 1 4 glucanase A GAQ11312.1
Talaromyces marneffei ATCC 18224 endoglucanase putative EEA28952.1
Scedosporium apiospermum SAPIO CDS6480 KEZ42063.1
Lichtheimia corymbifera JMRC FSU 9682 glycoside hydrolase family 12 protein CDH57791.1
Sporothrix schenckii 1099 18 SPSK 06582 KJR89555.1
Purpureocillium lilacinum glycoside hydrolase family 12 protein OAQ81277.1
Talaromyces marneffei ATCC 18224 endoglucanase putative EEA20285.1
Aspergillus fumigatus Af293 endoglucanase putative EAL86857.1
Aspergillus lentulus endoglucanase 1 GAQ05884.1
Aspergillus lentulus xyloglucan specific endo beta 1 4 glucanase A GAQ11719.1
Verruconis gallopava PV09 09243 KIV99014.1
Verruconis gallopava PV09 03599 KIW05743.1
Exophiala oligosperma PV06 01020 KIW48437.1
Exophiala oligosperma variant KIW48438.1
Cladophialophora bantiana CBS 173.52 Z519 02308 KIW96917.1
Rhinocladiella mackenziei CBS 650.93 Z518 01607 KIX10524.1
Talaromyces marneffei ATCC 18224 conserved EEA19401.1
Verruconis gallopava PV09 05592 KIW03385.1
Aspergillus fumigatus Af293 conserved EAL86643.2
Aspergillus lentulus xyloglucan specific endo beta 1 4 glucanase A GAQ04950.1
Sporothrix schenckii 1099 18 SPSK 09381 KJR86027.1
Purpureocillium lilacinum xyloglucan specific endoglucanase protein OAQ63931.1
Rieske
Ring_hydroxyl_A
Glyco_hydro_12
0.928
1
0.47
0.918
0.938
0.991
0.081
0.979
0.926
0.749
0.928
0.851
0.97
0.92
0.963
0.945
0.244
0.91
1
0.919
0.624
