# Supplementary material for: Diversity of Cell Wall Related Proteins in Human Pathogenic Fungi
Source: J Fungi (Basel). 2017 Dec 29;4(1):6. doi: 10.3390/jof4010006 (PMC5872309; doi:10.3390/jof4010006)
Supplement: Supplementary file 1 [file jof-04-00006-s001.zip › jof-252817-final-supplementary materials/Dataset S3_trees_pdf/GH128.pdf]

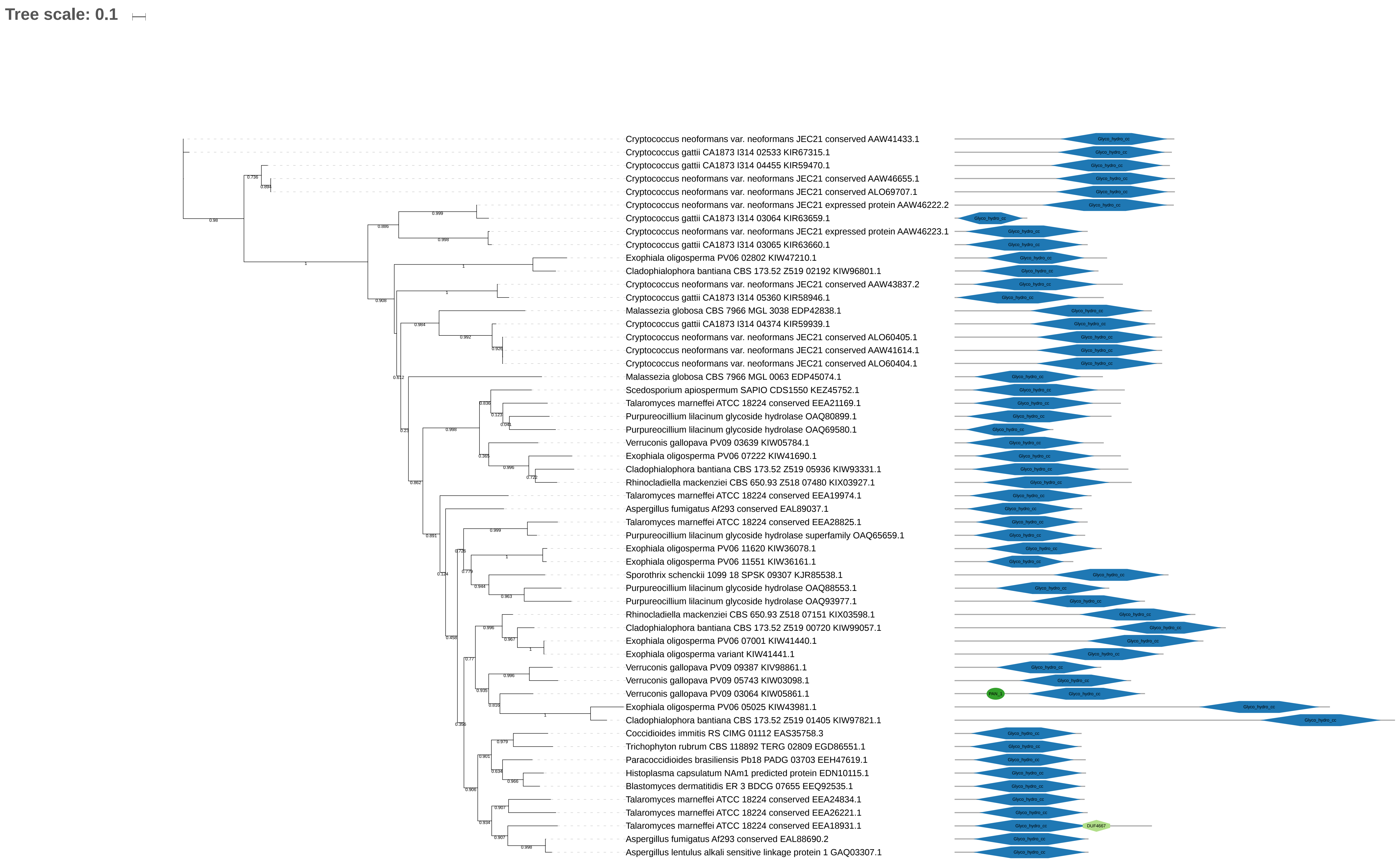
Tree scale: 0.1
Cryptococcus neoformans var. neoformans JEC21 conserved AAW41433.1
Cryptococcus gattii CA1873 I314 02533 KIR67315.1
Cryptococcus gattii CA1873 I314 04455 KIR59470.1
Cryptococcus neoformans var. neoformans JEC21 conserved AAW46655.1
Cryptococcus neoformans var. neoformans JEC21 conserved ALO69707.1
Cryptococcus neoformans var. neoformans JEC21 expressed protein AAW46222.2
Cryptococcus gattii CA1873 I314 03064 KIR63659.1
Cryptococcus neoformans var. neoformans JEC21 expressed protein AAW46223.1
Cryptococcus gattii CA1873 I314 03065 KIR63660.1
Exophiala oligosperma PV06 02802 KIW47210.1
Cladophialophora bantiana CBS 173.52 Z519 02192 KIW96801.1
Cryptococcus neoformans var. neoformans JEC21 conserved AAW43837.2
Cryptococcus gattii CA1873 I314 05360 KIR58946.1
Malassezia globosa CBS 7966 MGL 3038 EDP42838.1
Cryptococcus gattii CA1873 I314 04374 KIR59939.1
Cryptococcus neoformans var. neoformans JEC21 conserved ALO60405.1
Cryptococcus neoformans var. neoformans JEC21 conserved AAW41614.1
Cryptococcus neoformans var. neoformans JEC21 conserved ALO60404.1
Malassezia globosa CBS 7966 MGL 0063 EDP45074.1
Scedosporium apiospermum SAPIO CDS1550 KEZ45752.1
Talaromyces marneffei ATCC 18224 conserved EEA21169.1
Purpureocillium lilacinum glycoside hydrolase OAQ80899.1
Purpureocillium lilacinum glycoside hydrolase OAQ69580.1
Verruconis gallopava PV09 03639 KIW05784.1
Exophiala oligosperma PV06 07222 KIW41690.1
Cladophialophora bantiana CBS 173.52 Z519 05936 KIW93331.1
Rhinocladiella mackenziei CBS 650.93 Z518 07480 KIX03927.1
Talaromyces marneffei ATCC 18224 conserved EEA19974.1
Aspergillus fumigatus Af293 conserved EAL89037.1
Talaromyces marneffei ATCC 18224 conserved EEA28825.1
Purpureocillium lilacinum glycoside hydrolase superfamily OAQ65659.1
Exophiala oligosperma PV06 11620 KIW36078.1
Exophiala oligosperma PV06 11551 KIW36161.1
Sporothrix schenckii 1099 18 SPSK 09307 KJR85538.1
Purpureocillium lilacinum glycoside hydrolase OAQ88553.1
Purpureocillium lilacinum glycoside hydrolase OAQ93977.1
Rhinocladiella mackenziei CBS 650.93 Z518 07151 KIX03598.1
Cladophialophora bantiana CBS 173.52 Z519 00720 KIW99057.1
Exophiala oligosperma PV06 07001 KIW41440.1
Exophiala oligosperma variant KIW41441.1
Verruconis gallopava PV09 09387 KIV98861.1
Verruconis gallopava PV09 05743 KIW03098.1
Verruconis gallopava PV09 03064 KIW05861.1
Exophiala oligosperma PV06 05025 KIW43981.1
Cladophialophora bantiana CBS 173.52 Z519 01405 KIW97821.1
Coccidioides immitis RS CIMG 01112 EAS35758.3
Trichophyton rubrum CBS 118892 TERG 02809 EGD86551.1
Paracoccidioides brasiliensis Pb18 PADG 03703 EEH47619.1
Histoplasma capsulatum NAm1 predicted protein EDN10115.1
Blastomyces dermatitidis ER 3 BDCG 07655 EEQ92535.1
Talaromyces marneffei ATCC 18224 conserved EEA24834.1
Talaromyces marneffei ATCC 18224 conserved EEA26221.1
Talaromyces marneffei ATCC 18224 conserved EEA18931.1
Aspergillus fumigatus Af293 conserved EAL88690.2
Aspergillus lentulus alkali sensitive linkage protein 1 GAQ03307.1
