# Supplementary material for: Diversity of Cell Wall Related Proteins in Human Pathogenic Fungi
Source: J Fungi (Basel). 2017 Dec 29;4(1):6. doi: 10.3390/jof4010006 (PMC5872309; doi:10.3390/jof4010006)
Supplement: Supplementary file 1 [file jof-04-00006-s001.zip › jof-252817-final-supplementary materials/Dataset S3_trees_pdf/GH131.pdf]

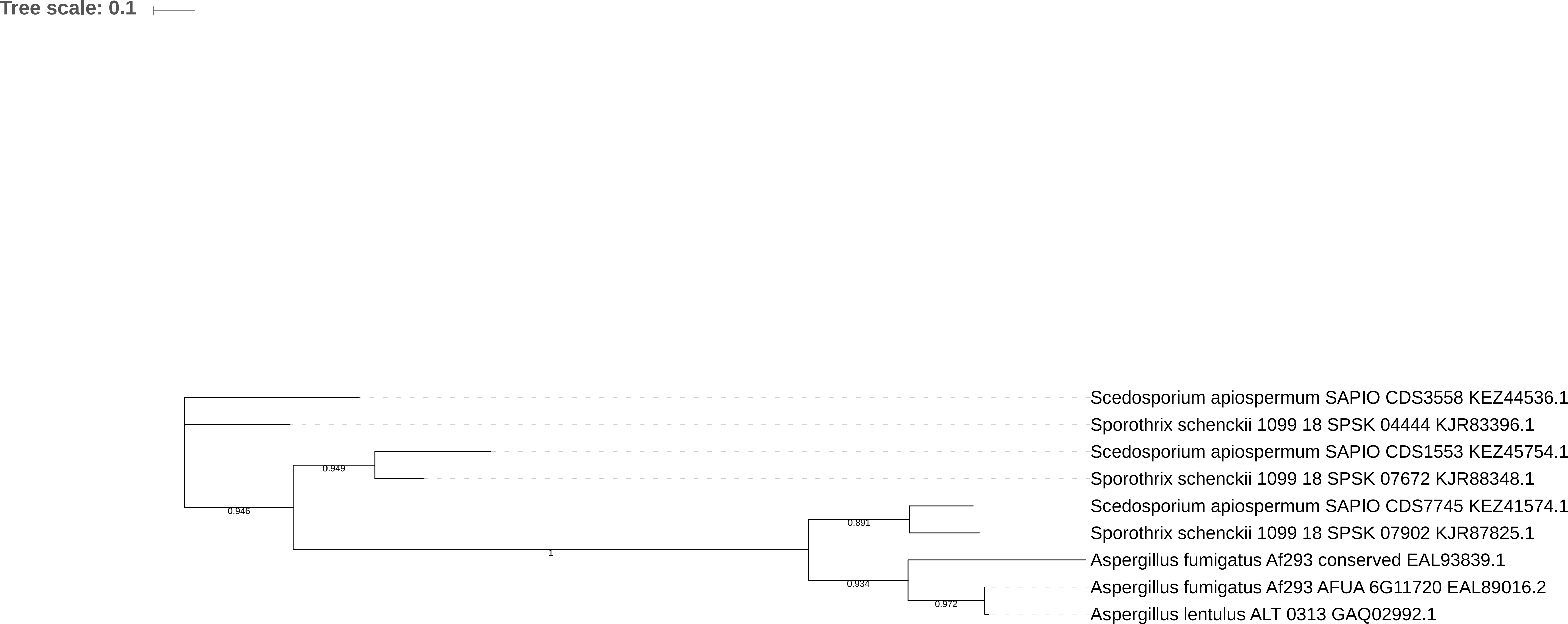
Tree scale: 0.1
Scedosporium apiospermum SAPIO CDS3558 KEZ44536.1
Sporothrix schenckii 1099 18 SPSK 04444 KJR83396.1
Scedosporium apiospermum SAPIO CDS1553 KEZ45754.1
Sporothrix schenckii 1099 18 SPSK 07672 KJR88348.1
Scedosporium apiospermum SAPIO CDS7745 KEZ41574.1
Sporothrix schenckii 1099 18 SPSK 07902 KJR87825.1
Aspergillus fumigatus Af293 conserved EAL93839.1
Aspergillus fumigatus Af293 AFUA 6G11720 EAL89016.2
Aspergillus lentulus ALT 0313 GAQ02992.1
0.949
0.946
1
0.891
0.934
0.972
