# Supplementary material for: Diversity of Cell Wall Related Proteins in Human Pathogenic Fungi
Source: J Fungi (Basel). 2017 Dec 29;4(1):6. doi: 10.3390/jof4010006 (PMC5872309; doi:10.3390/jof4010006)
Supplement: Supplementary file 1 [file jof-04-00006-s001.zip › jof-252817-final-supplementary materials/Dataset S3_trees_pdf/GH132.pdf]

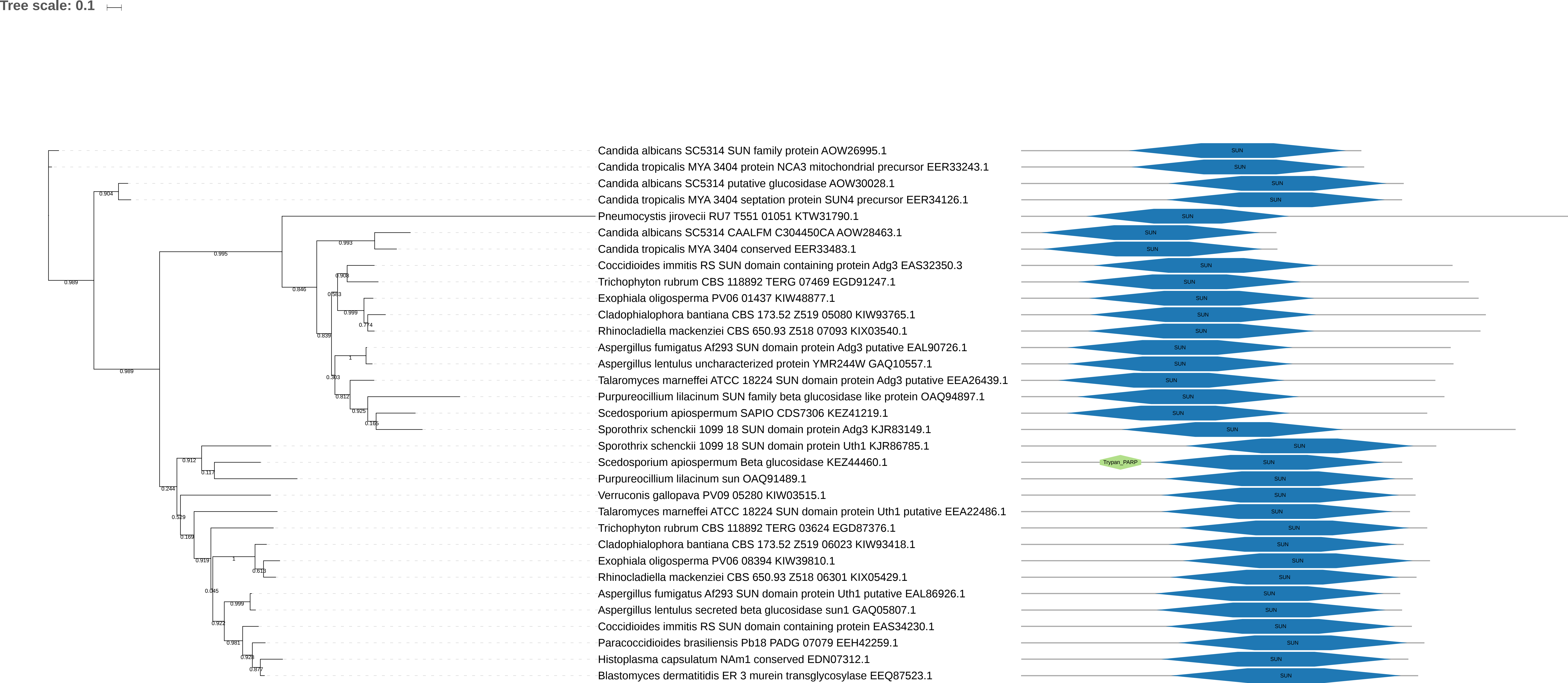
Tree scale: 0.1
Candida albicans SC5314 SUN family protein AOW26995.1
Candida tropicalis MYA 3404 protein NCA3 mitochondrial precursor EER33243.1
Candida albicans SC5314 putative glucosidase AOW30028.1
Candida tropicalis MYA 3404 septation protein SUN4 precursor EER34126.1
Pneumocystis jirovecii RU7 T551 01051 KTW31790.1
Candida albicans SC5314 CAALFM C304450CA AOW28463.1
Candida tropicalis MYA 3404 conserved EER33483.1
Coccidioides immitis RS SUN domain containing protein Adg3 EAS32350.3
Trichophyton rubrum CBS 118892 TERG 07469 EGD91247.1
Exophiala oligosperma PV06 01437 KIW48877.1
Cladophialophora bantiana CBS 173.52 Z519 05080 KIW93765.1
Rhinocladiella mackenziei CBS 650.93 Z518 07093 KIX03540.1
Aspergillus fumigatus Af293 SUN domain protein Adg3 putative EAL90726.1
Aspergillus lentulus uncharacterized protein YMR244W GAQ10557.1
Talaromyces marneffei ATCC 18224 SUN domain protein Adg3 putative EEA26439.1
Purpureocillium lilacinum SUN family beta glucosidase like protein OAQ94897.1
Scedosporium apiospermum SAPIO CDS7306 KEZ41219.1
Sporothrix schenckii 1099 18 SUN domain protein Adg3 KJR83149.1
Sporothrix schenckii 1099 18 SUN domain protein Uth1 KJR86785.1
Scedosporium apiospermum Beta glucosidase KEZ44460.1
Purpureocillium lilacinum sun OAQ91489.1
Verruconis gallopava PV09 05280 KIW03515.1
Talaromyces marneffei ATCC 18224 SUN domain protein Uth1 putative EEA22486.1
Trichophyton rubrum CBS 118892 TERG 03624 EGD87376.1
Cladophialophora bantiana CBS 173.52 Z519 06023 KIW93418.1
Exophiala oligosperma PV06 08394 KIW39810.1
Rhinocladiella mackenziei CBS 650.93 Z518 06301 KIX05429.1
Aspergillus fumigatus Af293 SUN domain protein Uth1 putative EAL86926.1
Aspergillus lentulus secreted beta glucosidase sun1 GAQ05807.1
Coccidioides immitis RS SUN domain containing protein EAS34230.1
Paracoccidioides brasiliensis Pb18 PADG 07079 EEH42259.1
Histoplasma capsulatum NAm1 conserved EDN07312.1
Blastomyces dermatitidis ER 3 murein transglycosylase EEQ87523.1
SUN
Trypan_PARP
