# Supplementary material for: Diversity of Cell Wall Related Proteins in Human Pathogenic Fungi
Source: J Fungi (Basel). 2017 Dec 29;4(1):6. doi: 10.3390/jof4010006 (PMC5872309; doi:10.3390/jof4010006)
Supplement: Supplementary file 1 [file jof-04-00006-s001.zip › jof-252817-final-supplementary materials/Dataset S3_trees_pdf/GH135.pdf]

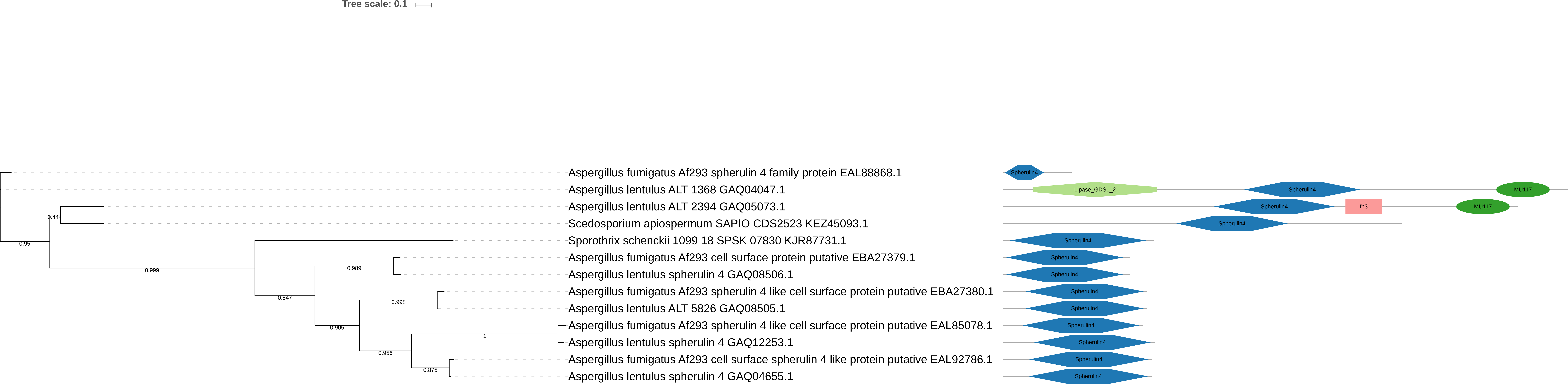
Tree scale: 0.1
Aspergillus fumigatus Af293 spherulin 4 family protein EAL88868.1
Aspergillus lentulus ALT 1368 GAQ04047.1
Aspergillus lentulus ALT 2394 GAQ05073.1
Scedosporium apiospermum SAPIO CDS2523 KEZ45093.1
Sporothrix schenckii 1099 18 SPSK 07830 KJR87731.1
Aspergillus fumigatus Af293 cell surface protein putative EBA27379.1
Aspergillus lentulus spherulin 4 GAQ08506.1
Aspergillus fumigatus Af293 spherulin 4 like cell surface protein putative EBA27380.1
Aspergillus lentulus ALT 5826 GAQ08505.1
Aspergillus fumigatus Af293 spherulin 4 like cell surface protein putative EAL85078.1
Aspergillus lentulus spherulin 4 GAQ12253.1
Aspergillus fumigatus Af293 cell surface spherulin 4 like protein putative EAL92786.1
Aspergillus lentulus spherulin 4 GAQ04655.1
0.444
0.95
0.999
0.847
0.989
0.998
0.905
1
0.956
0.875
Spherulin4
Lipase_GDSL_2
Spherulin4
MU117
Spherulin4
fn3
MU117
Spherulin4
