# Supplementary material for: Diversity of Cell Wall Related Proteins in Human Pathogenic Fungi
Source: J Fungi (Basel). 2017 Dec 29;4(1):6. doi: 10.3390/jof4010006 (PMC5872309; doi:10.3390/jof4010006)
Supplement: Supplementary file 1 [file jof-04-00006-s001.zip › jof-252817-final-supplementary materials/Dataset S3_trees_pdf/Gh17.pdf]

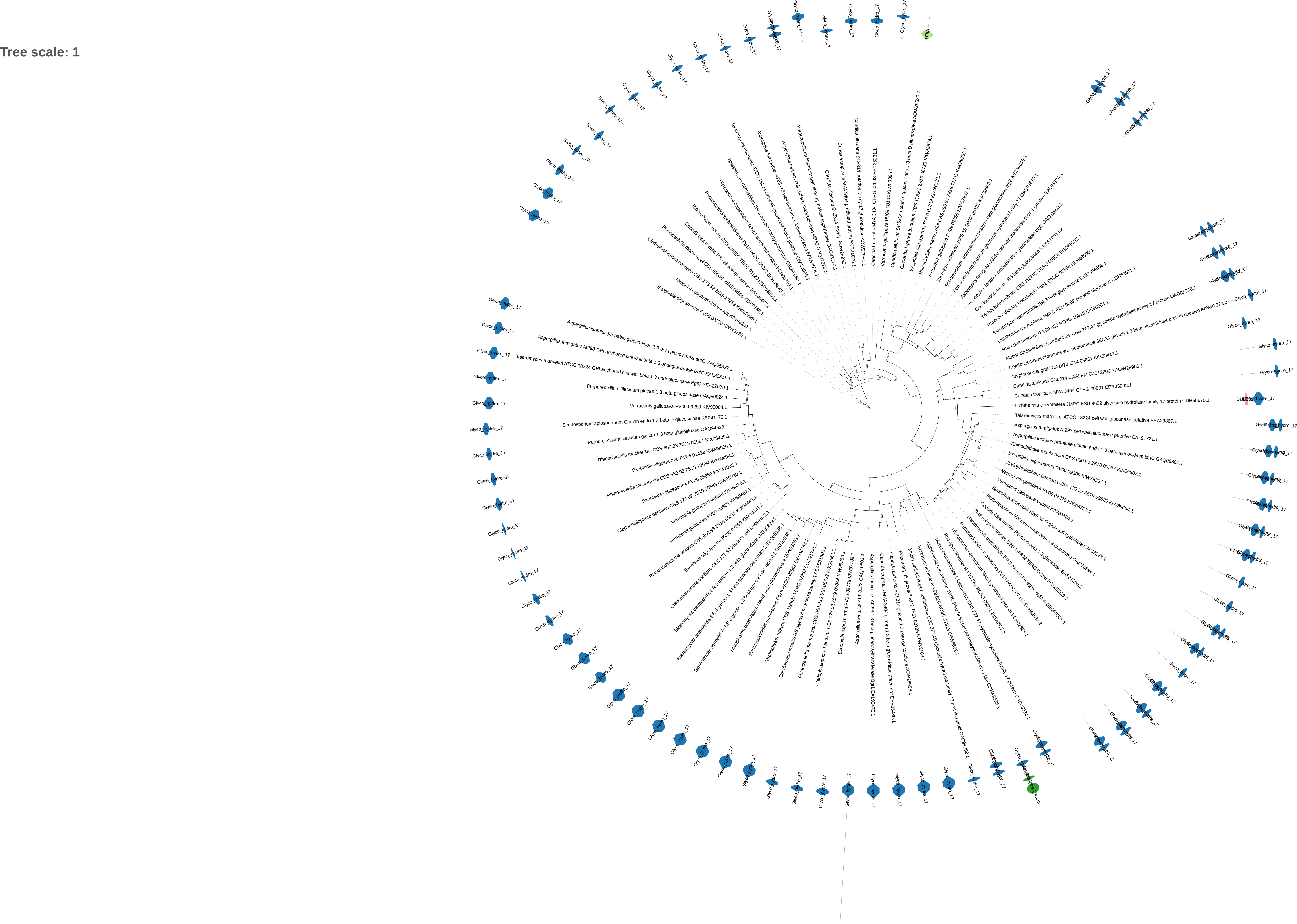

Tree scale: 1
Aspergillus lentulus probable glucan endo 1 3 beta glucosidase eglC GAQ05337.1
Aspergillus fumigatus Af293 GPI anchored cell wall beta 1 3 endoglucanase EglC EAL86311.1
Talaromyces marneffei ATCC 18224 GPI anchored cell wall beta 1 3 endoglucanase EglC EEA22070.1
Purpureocillium lilacinum glucan 1 3 beta glucosidase OAQ80824.1
Verruconis gallopava PV09 09283 KIV99004.1
Scedosporium apiospermum Glucan endo 1 3 beta D glucosidase KEZ41172.1
Purpureocillium lilacinum glucan 1 3 beta glucosidase OAQ94628.1
Lichtheimia corymbifera JMRC FSU 9682 cell wall glucanase CDH52611.1
Rhizopus delemar RA 99 880 RO3G 15315 EIE90604.1
Mucor circinelloides f. lusitanicus CBS 277.49 glycoside hydrolase family 17 protein OAD01936.1
Cryptococcus neoformans var. neoformans JEC21 glucan 1 3 beta glucosidase protein putative AAW47222.2
Cryptococcus gattii CA1873 I314 05661 KIR58417.1
Candida albicans SC5314 CAALFM C401220CA AOW28906.1
Candida tropicalis MYA 3404 CTRG 00031 EER35292.1
Lichtheimia corymbifera JMRC FSU 9682 glycoside hydrolase family 17 protein CDH50675.1
Talaromyces marneffei ATCC 18224 cell wall glucanase putative EEA23867.1
Aspergillus fumigatus Af293 cell wall glucanase putative EAL91721.1
Aspergillus lentulus probable glucan endo 1 3 beta glucosidase btgC GAQ09381.1
Rhinocladiella mackenziei CBS 650.93 Z518 00587 KIX09507.1
Exophiala oligosperma PV06 09309 KIW38337.1
Cladophialophora bantiana CBS 173.52 Z519 09820 KIW89664.1
Verruconis gallopava PV09 04278 KIW04523.1
Glyco_hydro_17
TFIIA
