# Supplementary material for: Diversity of Cell Wall Related Proteins in Human Pathogenic Fungi
Source: J Fungi (Basel). 2017 Dec 29;4(1):6. doi: 10.3390/jof4010006 (PMC5872309; doi:10.3390/jof4010006)
Supplement: Supplementary file 1 [file jof-04-00006-s001.zip › jof-252817-final-supplementary materials/Dataset S3_trees_pdf/GH2.pdf]

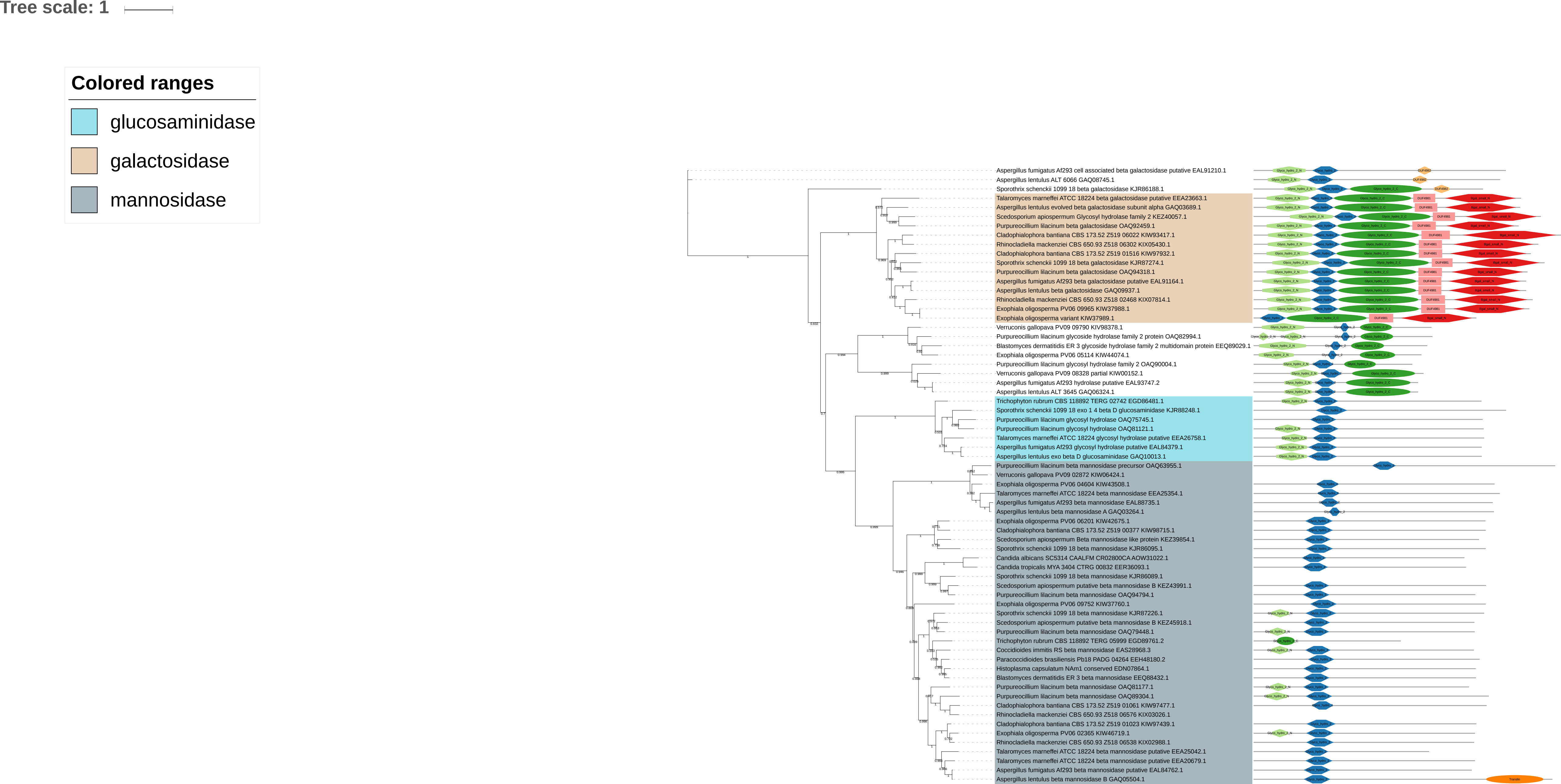
Tree scale: 1
Colored ranges
glucosaminidase
galactosidase
mannosidase
Aspergillus fumigatus Af293 cell associated beta galactosidase putative EAL91210.1
Aspergillus lentulus ALT 6066 GAQ08745.1
Sporothrix schenckii 1099 18 beta galactosidase KJR86188.1
Talaromyces marneffei ATCC 18224 beta galactosidase putative EEA23663.1
Aspergillus lentulus evolved beta galactosidase subunit alpha GAQ03689.1
Scedosporium apiospermum Glycosyl hydrolase family 2 KEZ40057.1
Purpureocillium lilacinum beta galactosidase OAQ92459.1
Cladophialophora bantiana CBS 173.52 Z519 06022 KIW93417.1
Rhinocladiella mackenziei CBS 650.93 Z518 06302 KIX05430.1
Cladophialophora bantiana CBS 173.52 Z519 01516 KIW97932.1
Sporothrix schenckii 1099 18 beta galactosidase KJR87274.1
Purpureocillium lilacinum beta galactosidase OAQ94318.1
Aspergillus fumigatus Af293 beta galactosidase putative EAL91164.1
Aspergillus lentulus beta galactosidase GAQ09937.1
Rhinocladiella mackenziei CBS 650.93 Z518 02468 KIX07814.1
Exophiala oligosperma PV06 09965 KIW37988.1
Exophiala oligosperma variant KIW37989.1
Verruconis gallopava PV09 09790 KIV98378.1
Purpureocillium lilacinum glycoside hydrolase family 2 protein OAQ82994.1
Blastomyces dermatitidis ER 3 glycoside hydrolase family 2 multidomain protein EEQ89029.1
Exophiala oligosperma PV06 05114 KIW44074.1
Purpureocillium lilacinum glycosyl hydrolase family 2 OAQ90004.1
Verruconis gallopava PV09 08328 partial KIW00152.1
Aspergillus fumigatus Af293 hydrolase putative EAL93747.2
Aspergillus lentulus ALT 3645 GAQ06324.1
Trichophyton rubrum CBS 118892 TERG 02742 EGD86481.1
Sporothrix schenckii 1099 18 exo 1 4 beta D glucosaminidase KJR88248.1
Purpureocillium lilacinum glycosyl hydrolase OAQ75745.1
Purpureocillium lilacinum glycosyl hydrolase OAQ81121.1
Talaromyces marneffei ATCC 18224 glycosyl hydrolase putative EEA26758.1
Aspergillus fumigatus Af293 glycosyl hydrolase putative EAL84379.1
Aspergillus lentulus exo beta D glucosaminidase GAQ10013.1
Purpureocillium lilacinum beta mannosidase precursor OAQ63955.1
Verruconis gallopava PV09 02872 KIW06424.1
Exophiala oligosperma PV06 04604 KIW43508.1
Talaromyces marneffei ATCC 18224 beta mannosidase EEA25354.1
Aspergillus fumigatus Af293 beta mannosidase EAL88735.1
Aspergillus lentulus beta mannosidase A GAQ03264.1
Exophiala oligosperma PV06 06201 KIW42675.1
Cladophialophora bantiana CBS 173.52 Z519 00377 KIW98715.1
Scedosporium apiospermum Beta mannosidase like protein KEZ39854.1
Sporothrix schenckii 1099 18 beta mannosidase KJR86095.1
Candida albicans SC5314 CAALFM CR02800CA AOW31022.1
Candida tropicalis MYA 3404 CTRG 00832 EER36093.1
Sporothrix schenckii 1099 18 beta mannosidase KJR86089.1
Scedosporium apiospermum putative beta mannosidase B KEZ43991.1
Purpureocillium lilacinum beta mannosidase OAQ94794.1
Exophiala oligosperma PV06 09752 KIW37760.1
Sporothrix schenckii 1099 18 beta mannosidase KJR87226.1
Scedosporium apiospermum putative beta mannosidase B KEZ45918.1
Purpureocillium lilacinum beta mannosidase OAQ79448.1
Trichophyton rubrum CBS 118892 TERG 05999 EGD89761.2
Coccidioides immitis RS beta mannosidase EAS28968.3
Paracoccidioides brasiliensis Pb18 PADG 04264 EEH48180.2
Histoplasma capsulatum NAm1 conserved EDN07864.1
Blastomyces dermatitidis ER 3 beta mannosidase EEQ88432.1
Purpureocillium lilacinum beta mannosidase OAQ81177.1
Purpureocillium lilacinum beta mannosidase OAQ89304.1
Cladophialophora bantiana CBS 173.52 Z519 01061 KIW97477.1
Rhinocladiella mackenziei CBS 650.93 Z518 06576 KIX03026.1
Cladophialophora bantiana CBS 173.52 Z519 01023 KIW97439.1
Exophiala oligosperma PV06 02365 KIW46719.1
Rhinocladiella mackenziei CBS 650.93 Z518 06538 KIX02988.1
Talaromyces marneffei ATCC 18224 beta mannosidase putative EEA25042.1
Talaromyces marneffei ATCC 18224 beta mannosidase putative EEA20679.1
Aspergillus fumigatus Af293 beta mannosidase putative EAL84762.1
Aspergillus lentulus beta mannosidase B GAQ05504.1
