# Supplementary material for: Diversity of Cell Wall Related Proteins in Human Pathogenic Fungi
Source: J Fungi (Basel). 2017 Dec 29;4(1):6. doi: 10.3390/jof4010006 (PMC5872309; doi:10.3390/jof4010006)
Supplement: Supplementary file 1 [file jof-04-00006-s001.zip › jof-252817-final-supplementary materials/Dataset S3_trees_pdf/GH20.pdf]

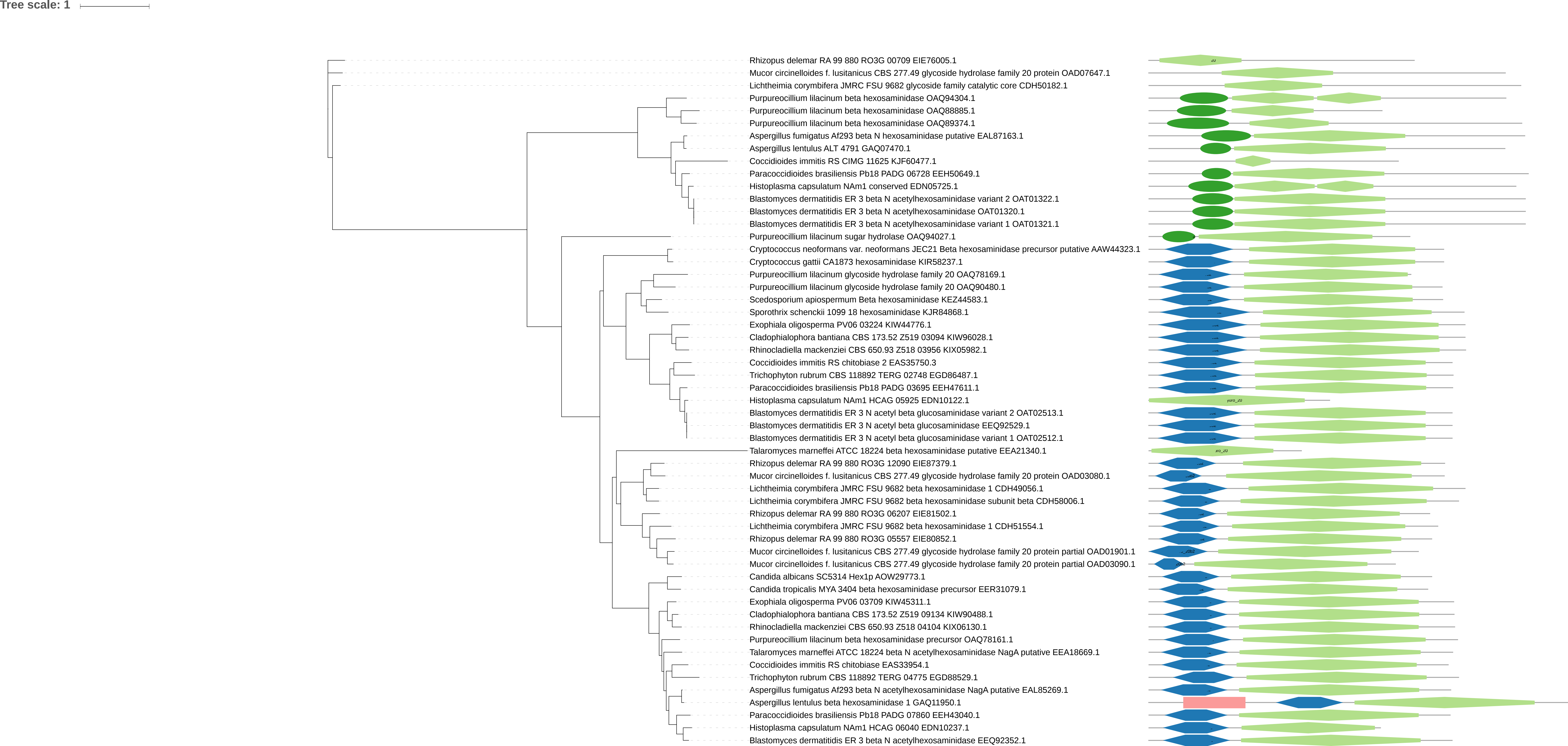

Tree scale: 1
Rhizopus delemar RA 99 880 RO3G 00709 EIE76005.1
Mucor circinelloides f. lusitanicus CBS 277.49 glycoside hydrolase family 20 protein OAD07647.1
Lichtheimia corymbifera JMRC FSU 9682 glycoside family catalytic core CDH50182.1
Purpureocillium lilacinum beta hexosaminidase OAQ94304.1
Purpureocillium lilacinum beta hexosaminidase OAQ88885.1
Purpureocillium lilacinum beta hexosaminidase OAQ89374.1
Aspergillus fumigatus Af293 beta N hexosaminidase putative EAL87163.1
Aspergillus lentulus ALT 4791 GAQ07470.1
Coccidioides immitis RS CIMG 11625 KJF60477.1
Paracoccidioides brasiliensis Pb18 PADG 06728 EEH50649.1
Histoplasma capsulatum NAm1 conserved EDN05725.1
Blastomyces dermatitidis ER 3 beta N acetylhexosaminidase variant 2 OAT01322.1
Blastomyces dermatitidis ER 3 beta N acetylhexosaminidase OAT01320.1
Blastomyces dermatitidis ER 3 beta N acetylhexosaminidase variant 1 OAT01321.1
Purpureocillium lilacinum sugar hydrolase OAQ94027.1
Cryptococcus neoformans var. neoformans JEC21 Beta hexosaminidase precursor putative AAW44323.1
Cryptococcus gattii CA1873 hexosaminidase KIR58237.1
Purpureocillium lilacinum glycoside hydrolase family 20 OAQ78169.1
Purpureocillium lilacinum glycoside hydrolase family 20 OAQ90480.1
Scedosporium apiospermum Beta hexosaminidase KEZ44583.1
Sporothrix schenckii 1099 18 hexosaminidase KJR84868.1
Exophiala oligosperma PV06 03224 KIW44776.1
Cladophialophora bantiana CBS 173.52 Z519 03094 KIW96028.1
Rhinocladiella mackenziei CBS 650.93 Z518 03956 KIX05982.1
Coccidioides immitis RS chitobiase 2 EAS35750.3
Trichophyton rubrum CBS 118892 TERG 02748 EGD86487.1
Paracoccidioides brasiliensis Pb18 PADG 03695 EEH47611.1
Histoplasma capsulatum NAm1 HCAG 05925 EDN10122.1
Blastomyces dermatitidis ER 3 N acetyl beta glucosaminidase variant 2 OAT02513.1
Blastomyces dermatitidis ER 3 N acetyl beta glucosaminidase EEQ92529.1
Blastomyces dermatitidis ER 3 N acetyl beta glucosaminidase variant 1 OAT02512.1
Talaromyces marneffei ATCC 18224 beta hexosaminidase putative EEA21340.1
Rhizopus delemar RA 99 880 RO3G 12090 EIE87379.1
Mucor circinelloides f. lusitanicus CBS 277.49 glycoside hydrolase family 20 protein OAD03080.1
Lichtheimia corymbifera JMRC FSU 9682 beta hexosaminidase 1 CDH49056.1
Lichtheimia corymbifera JMRC FSU 9682 beta hexosaminidase subunit beta CDH58006.1
Rhizopus delemar RA 99 880 RO3G 06207 EIE81502.1
Lichtheimia corymbifera JMRC FSU 9682 beta hexosaminidase 1 CDH51554.1
Rhizopus delemar RA 99 880 RO3G 05557 EIE80852.1
Mucor circinelloides f. lusitanicus CBS 277.49 glycoside hydrolase family 20 protein partial OAD01901.1
Mucor circinelloides f. lusitanicus CBS 277.49 glycoside hydrolase family 20 protein partial OAD03090.1
Candida albicans SC5314 Hex1p AOW29773.1
Candida tropicalis MYA 3404 beta hexosaminidase precursor EER31079.1
Exophiala oligosperma PV06 03709 KIW45311.1
Cladophialophora bantiana CBS 173.52 Z519 09134 KIW90488.1
Rhinocladiella mackenziei CBS 650.93 Z518 04104 KIX06130.1
Purpureocillium lilacinum beta hexosaminidase precursor OAQ78161.1
Talaromyces marneffei ATCC 18224 beta N acetylhexosaminidase NagA putative EEA18669.1
Coccidioides immitis RS chitobiase EAS33954.1
Trichophyton rubrum CBS 118892 TERG 04775 EGD88529.1
Aspergillus fumigatus Af293 beta N acetylhexosaminidase NagA putative EAL85269.1
Aspergillus lentulus beta hexosaminidase 1 GAQ11950.1
Paracoccidioides brasiliensis Pb18 PADG 07860 EEH43040.1
Histoplasma capsulatum NAm1 HCAG 06040 EDN10237.1
Blastomyces dermatitidis ER 3 beta N acetylhexosaminidase EEQ92352.1
