# Supplementary material for: Diversity of Cell Wall Related Proteins in Human Pathogenic Fungi
Source: J Fungi (Basel). 2017 Dec 29;4(1):6. doi: 10.3390/jof4010006 (PMC5872309; doi:10.3390/jof4010006)
Supplement: Supplementary file 1 [file jof-04-00006-s001.zip › jof-252817-final-supplementary materials/Dataset S3_trees_pdf/GH27.pdf]

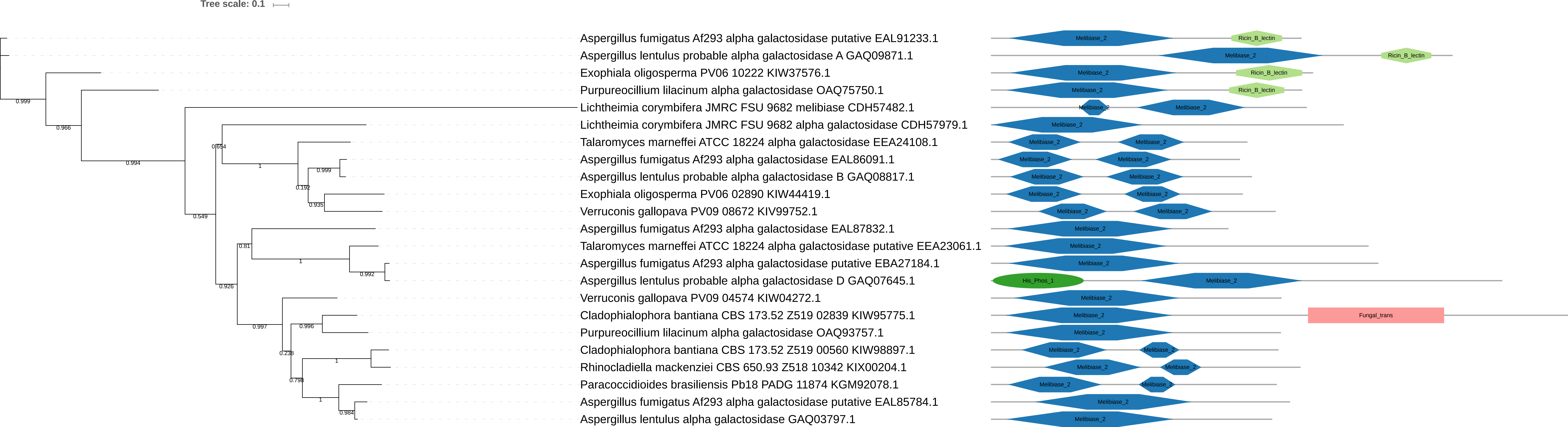
Tree scale: 0.1
Aspergillus fumigatus Af293 alpha galactosidase putative EAL91233.1
Aspergillus lentulus probable alpha galactosidase A GAQ09871.1
Exophiala oligosperma PV06 10222 KIW37576.1
Purpureocillium lilacinum alpha galactosidase OAQ75750.1
Lichtheimia corymbifera JMRC FSU 9682 melibiase CDH57482.1
Lichtheimia corymbifera JMRC FSU 9682 alpha galactosidase CDH57979.1
Talaromyces marneffei ATCC 18224 alpha galactosidase EEA24108.1
Aspergillus fumigatus Af293 alpha galactosidase EAL86091.1
Aspergillus lentulus probable alpha galactosidase B GAQ08817.1
Exophiala oligosperma PV06 02890 KIW44419.1
Verruconis gallopava PV09 08672 KIV99752.1
Aspergillus fumigatus Af293 alpha galactosidase EAL87832.1
Talaromyces marneffei ATCC 18224 alpha galactosidase putative EEA23061.1
Aspergillus fumigatus Af293 alpha galactosidase putative EBA27184.1
Aspergillus lentulus probable alpha galactosidase D GAQ07645.1
Verruconis gallopava PV09 04574 KIW04272.1
Cladophialophora bantiana CBS 173.52 Z519 02839 KIW95775.1
Purpureocillium lilacinum alpha galactosidase OAQ93757.1
Cladophialophora bantiana CBS 173.52 Z519 00560 KIW98897.1
Rhinocladiella mackenziei CBS 650.93 Z518 10342 KIX00204.1
Paracoccidioides brasiliensis Pb18 PADG 11874 KGM92078.1
Aspergillus fumigatus Af293 alpha galactosidase putative EAL85784.1
Aspergillus lentulus alpha galactosidase GAQ03797.1
0.999
0.966
0.994
0.654
1
0.999
0.192
0.935
0.549
0.81
1
0.992
0.926
0.997
0.996
0.238
1
0.798
1
0.984
Melibiase_2
Ricin_B_lectin
His_Phos_1
Fungal_trans
