# Supplementary material for: Diversity of Cell Wall Related Proteins in Human Pathogenic Fungi
Source: J Fungi (Basel). 2017 Dec 29;4(1):6. doi: 10.3390/jof4010006 (PMC5872309; doi:10.3390/jof4010006)
Supplement: Supplementary file 1 [file jof-04-00006-s001.zip › jof-252817-final-supplementary materials/Dataset S3_trees_pdf/GH28.pdf]

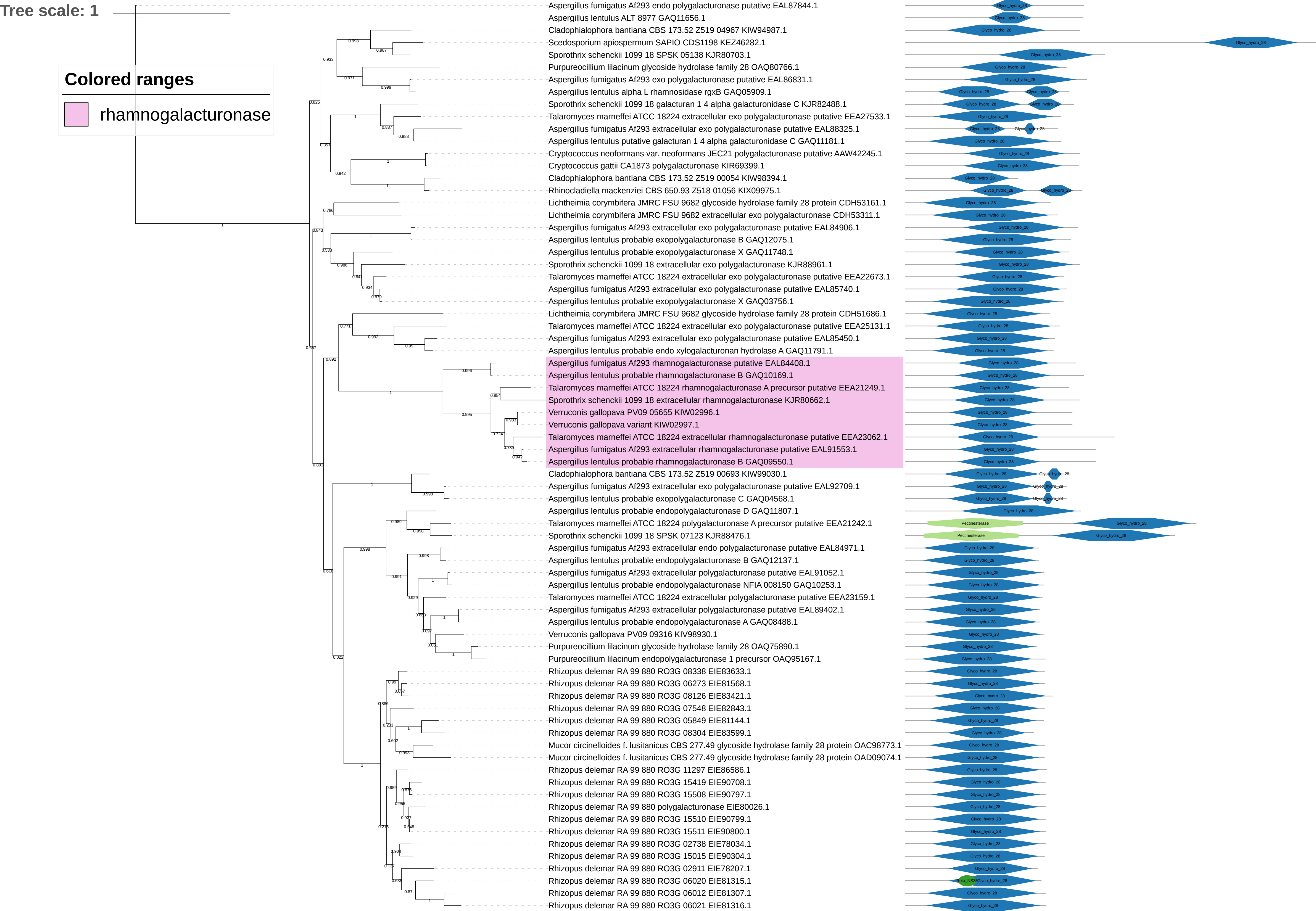

Tree scale: 1
Colored ranges
rhamnogalacturonase
Aspergillus fumigatus Af293 endo polygalacturonase putative EAL87844.1
Aspergillus lentulus ALT 8977 GAQ11656.1
Cladophialophora bantiana CBS 173.52 Z519 04967 KIW94987.1
Scedosporium apiospermum SAPIO CDS1198 KEZ46282.1
Sporothrix schenckii 1099 18 SPSK 05138 KJR80703.1
Purpureocillium lilacinum glycoside hydrolase family 28 OAQ80766.1
Aspergillus fumigatus Af293 exo polygalacturonase putative EAL86831.1
Aspergillus lentulus alpha L rhamnosidase rgxB GAQ05909.1
Sporothrix schenckii 1099 18 galacturan 1 4 alpha galacturonidase C KJR82488.1
Talaromyces marneffei ATCC 18224 extracellular exo polygalacturonase putative EEA27533.1
Aspergillus fumigatus Af293 extracellular exo polygalacturonase putative EAL88325.1
Aspergillus lentulus putative galacturan 1 4 alpha galacturonidase C GAQ11181.1
Cryptococcus neoformans var. neoformans JEC21 polygalacturonase putative AAW42245.1
Cryptococcus gattii CA1873 polygalacturonase KIR69399.1
Cladophialophora bantiana CBS 173.52 Z519 00054 KIW98394.1
Rhinocladiella mackenziei CBS 650.93 Z518 01056 KIX09975.1
Lichtheimia corymbifera JMRC FSU 9682 glycoside hydrolase family 28 protein CDH53161.1
Lichtheimia corymbifera JMRC FSU 9682 extracellular exo polygalacturonase CDH53311.1
Aspergillus fumigatus Af293 extracellular exo polygalacturonase putative EAL84906.1
Aspergillus lentulus probable exopolygalacturonase B GAQ12075.1
Aspergillus lentulus probable exopolygalacturonase X GAQ11748.1
Sporothrix schenckii 1099 18 extracellular exo polygalacturonase KJR88961.1
Talaromyces marneffei ATCC 18224 extracellular exo polygalacturonase putative EEA22673.1
Aspergillus fumigatus Af293 extracellular exo polygalacturonase putative EAL85740.1
Aspergillus lentulus probable exopolygalacturonase X GAQ03756.1
Lichtheimia corymbifera JMRC FSU 9682 glycoside hydrolase family 28 protein CDH51686.1
Talaromyces marneffei ATCC 18224 extracellular exo polygalacturonase putative EEA25131.1
Aspergillus fumigatus Af293 extracellular exo polygalacturonase putative EAL85450.1
Aspergillus lentulus probable endo xylogalacturonan hydrolase A GAQ11791.1
Aspergillus fumigatus Af293 rhamnogalacturonase putative EAL84408.1
Aspergillus lentulus probable rhamnogalacturonase B GAQ10169.1
Talaromyces marneffei ATCC 18224 rhamnogalacturonase A precursor putative EEA21249.1
Sporothrix schenckii 1099 18 extracellular rhamnogalacturonase KJR80662.1
Verruconis gallopava PV09 05655 KIW02996.1
Verruconis gallopava variant KIW02997.1
Talaromyces marneffei ATCC 18224 extracellular rhamnogalacturonase putative EEA23062.1
Aspergillus fumigatus Af293 extracellular rhamnogalacturonase putative EAL91553.1
Aspergillus lentulus probable rhamnogalacturonase B GAQ09550.1
Cladophialophora bantiana CBS 173.52 Z519 00693 KIW99030.1
Aspergillus fumigatus Af293 extracellular exo polygalacturonase putative EAL92709.1
Aspergillus lentulus probable exopolygalacturonase C GAQ04568.1
Aspergillus lentulus probable endopolygalacturonase D GAQ11807.1
Talaromyces marneffei ATCC 18224 polygalacturonase A precursor putative EEA21242.1
Sporothrix schenckii 1099 18 SPSK 07123 KJR88476.1
Aspergillus fumigatus Af293 extracellular endo polygalacturonase putative EAL84971.1
Aspergillus lentulus probable endopolygalacturonase B GAQ12137.1
Aspergillus fumigatus Af293 extracellular polygalacturonase putative EAL91052.1
Aspergillus lentulus probable endopolygalacturonase NFIA 008150 GAQ10253.1
Talaromyces marneffei ATCC 18224 extracellular polygalacturonase putative EEA23159.1
Aspergillus fumigatus Af293 extracellular polygalacturonase putative EAL89402.1
Aspergillus lentulus probable endopolygalacturonase A GAQ08488.1
Verruconis gallopava PV09 09316 KIV98930.1
Purpureocillium lilacinum glycoside hydrolase family 28 OAQ75890.1
Purpureocillium lilacinum endopolygalacturonase 1 precursor OAQ95167.1
Rhizopus delemar RA 99 880 RO3G 08338 EIE83633.1
Rhizopus delemar RA 99 880 RO3G 06273 EIE81568.1
Rhizopus delemar RA 99 880 RO3G 08126 EIE83421.1
Rhizopus delemar RA 99 880 RO3G 07548 EIE82843.1
Rhizopus delemar RA 99 880 RO3G 05849 EIE81144.1
Rhizopus delemar RA 99 880 RO3G 08304 EIE83599.1
Mucor circinelloides f. lusitanicus CBS 277.49 glycoside hydrolase family 28 protein OAC98773.1
Mucor circinelloides f. lusitanicus CBS 277.49 glycoside hydrolase family 28 protein OAD09074.1
Rhizopus delemar RA 99 880 RO3G 11297 EIE86586.1
Rhizopus delemar RA 99 880 RO3G 15419 EIE90708.1
Rhizopus delemar RA 99 880 RO3G 15508 EIE90797.1
Rhizopus delemar RA 99 880 polygalacturonase EIE80026.1
Rhizopus delemar RA 99 880 RO3G 15510 EIE90799.1
Rhizopus delemar RA 99 880 RO3G 15511 EIE90800.1
Rhizopus delemar RA 99 880 RO3G 02738 EIE78034.1
Rhizopus delemar RA 99 880 RO3G 15015 EIE90304.1
Rhizopus delemar RA 99 880 RO3G 02911 EIE78207.1
Rhizopus delemar RA 99 880 RO3G 06020 EIE81315.1
Rhizopus delemar RA 99 880 RO3G 06012 EIE81307.1
Rhizopus delemar RA 99 880 RO3G 06021 EIE81316.1
Pectinesterase
Glyco_hydro_28
