# Supplementary material for: Diversity of Cell Wall Related Proteins in Human Pathogenic Fungi
Source: J Fungi (Basel). 2017 Dec 29;4(1):6. doi: 10.3390/jof4010006 (PMC5872309; doi:10.3390/jof4010006)
Supplement: Supplementary file 1 [file jof-04-00006-s001.zip › jof-252817-final-supplementary materials/Dataset S3_trees_pdf/GH29.pdf]

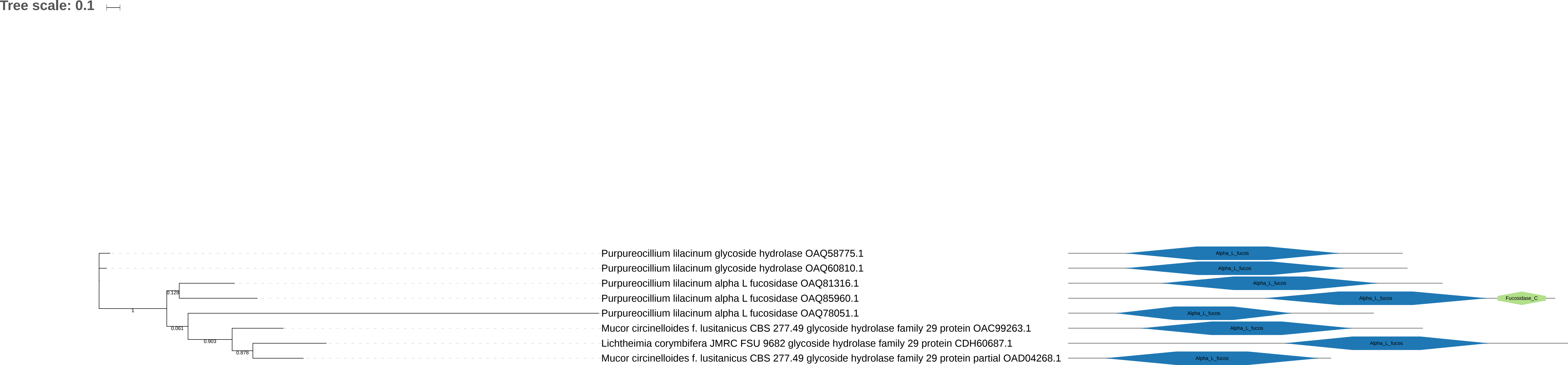
Tree scale: 0.1
Purpureocillium lilacinum glycoside hydrolase OAQ58775.1
Purpureocillium lilacinum glycoside hydrolase OAQ60810.1
Purpureocillium lilacinum alpha L fucosidase OAQ81316.1
Purpureocillium lilacinum alpha L fucosidase OAQ85960.1
Purpureocillium lilacinum alpha L fucosidase OAQ78051.1
Mucor circinelloides f. lusitanicus CBS 277.49 glycoside hydrolase family 29 protein OAC99263.1
Lichtheimia corymbifera JMRC FSU 9682 glycoside hydrolase family 29 protein CDH60687.1
Mucor circinelloides f. lusitanicus CBS 277.49 glycoside hydrolase family 29 protein partial OAD04268.1
0.128
1
0.061
0.903
0.878
Alpha_L_fucos
Fucosidase_C
