# Supplementary material for: Diversity of Cell Wall Related Proteins in Human Pathogenic Fungi
Source: J Fungi (Basel). 2017 Dec 29;4(1):6. doi: 10.3390/jof4010006 (PMC5872309; doi:10.3390/jof4010006)
Supplement: Supplementary file 1 [file jof-04-00006-s001.zip › jof-252817-final-supplementary materials/Dataset S3_trees_pdf/GH30.pdf]

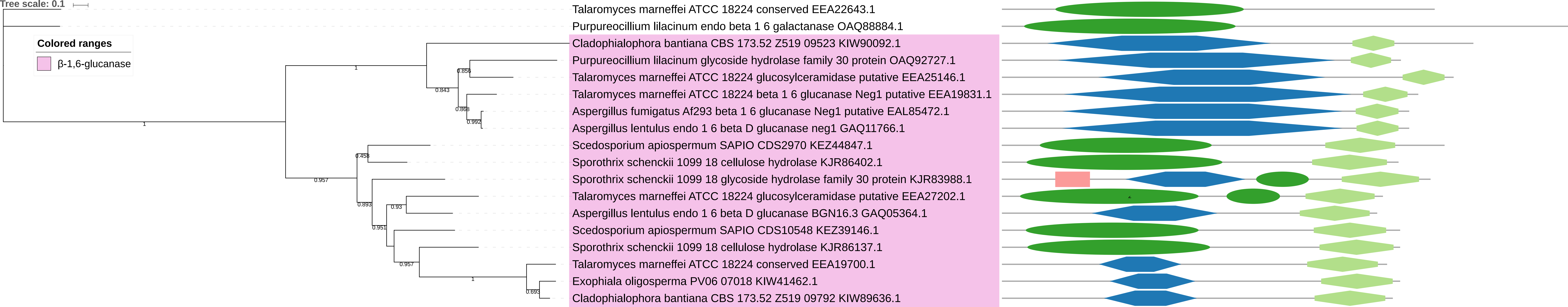

Tree scale: 0.1
Colored ranges
β-1,6-glucanase
Talaromyces marneffei ATCC 18224 conserved EEA22643.1
Purpureocillium lilacinum endo beta 1 6 galactanase OAQ88884.1
Cladophialophora bantiana CBS 173.52 Z519 09523 KIW90092.1
Purpureocillium lilacinum glycoside hydrolase family 30 protein OAQ92727.1
Talaromyces marneffei ATCC 18224 glucosylceramidase putative EEA25146.1
Talaromyces marneffei ATCC 18224 beta 1 6 glucanase Neg1 putative EEA19831.1
Aspergillus fumigatus Af293 beta 1 6 glucanase Neg1 putative EAL85472.1
Aspergillus lentulus endo 1 6 beta D glucanase neg1 GAQ11766.1
Scedosporium apiospermum SAPIO CDS2970 KEZ44847.1
Sporothrix schenckii 1099 18 cellulose hydrolase KJR86402.1
Sporothrix schenckii 1099 18 glycoside hydrolase family 30 protein KJR83988.1
Talaromyces marneffei ATCC 18224 glucosylceramidase putative EEA27202.1
Aspergillus lentulus endo 1 6 beta D glucanase BGN16.3 GAQ05364.1
Scedosporium apiospermum SAPIO CDS10548 KEZ39146.1
Sporothrix schenckii 1099 18 cellulose hydrolase KJR86137.1
Talaromyces marneffei ATCC 18224 conserved EEA19700.1
Exophiala oligosperma PV06 07018 KIW41462.1
Cladophialophora bantiana CBS 173.52 Z519 09792 KIW89636.1
1
1
0.856
0.843
0.868
0.992
0.458
0.957
0.893
0.93
0.951
0.957
1
0.693
