# Supplementary material for: Diversity of Cell Wall Related Proteins in Human Pathogenic Fungi
Source: J Fungi (Basel). 2017 Dec 29;4(1):6. doi: 10.3390/jof4010006 (PMC5872309; doi:10.3390/jof4010006)
Supplement: Supplementary file 1 [file jof-04-00006-s001.zip › jof-252817-final-supplementary materials/Dataset S3_trees_pdf/Gh38.pdf]

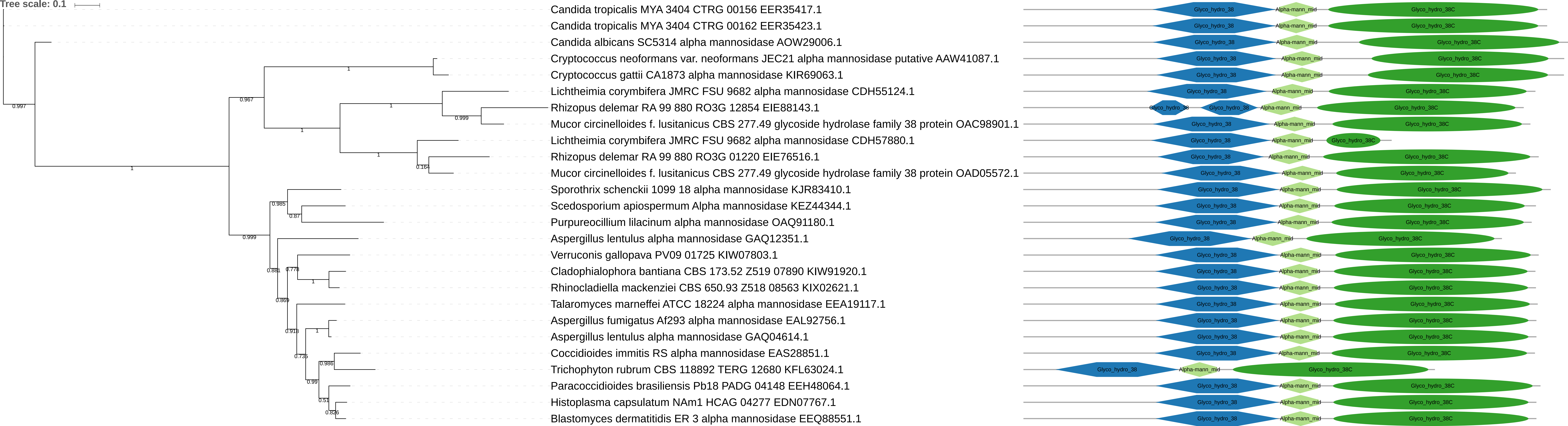

Tree scale: 0.1
Candida tropicalis MYA 3404 CTRG 00156 EER35417.1
Candida tropicalis MYA 3404 CTRG 00162 EER35423.1
Candida albicans SC5314 alpha mannosidase AOW29006.1
Cryptococcus neoformans var. neoformans JEC21 alpha mannosidase putative AAW41087.1
Cryptococcus gattii CA1873 alpha mannosidase KIR69063.1
Lichtheimia corymbifera JMRC FSU 9682 alpha mannosidase CDH55124.1
Rhizopus delemar RA 99 880 RO3G 12854 EIE88143.1
Mucor circinelloides f. lusitanicus CBS 277.49 glycoside hydrolase family 38 protein OAC98901.1
Lichtheimia corymbifera JMRC FSU 9682 alpha mannosidase CDH57880.1
Rhizopus delemar RA 99 880 RO3G 01220 EIE76516.1
Mucor circinelloides f. lusitanicus CBS 277.49 glycoside hydrolase family 38 protein OAD05572.1
Sporothrix schenckii 1099 18 alpha mannosidase KJR83410.1
Scedosporium apiospermum Alpha mannosidase KEZ44344.1
Purpureocillium lilacinum alpha mannosidase OAQ91180.1
Aspergillus lentulus alpha mannosidase GAQ12351.1
Verruconis gallopava PV09 01725 KIW07803.1
Cladophialophora bantiana CBS 173.52 Z519 07890 KIW91920.1
Rhinocladiella mackenziei CBS 650.93 Z518 08563 KIX02621.1
Talaromyces marneffei ATCC 18224 alpha mannosidase EEA19117.1
Aspergillus fumigatus Af293 alpha mannosidase EAL92756.1
Aspergillus lentulus alpha mannosidase GAQ04614.1
Coccidioides immitis RS alpha mannosidase EAS28851.1
Trichophyton rubrum CBS 118892 TERG 12680 KFL63024.1
Paracoccidioides brasiliensis Pb18 PADG 04148 EEH48064.1
Histoplasma capsulatum NAm1 HCAG 04277 EDN07767.1
Blastomyces dermatitidis ER 3 alpha mannosidase EEQ88551.1
0.997
1
0.967
1
1
0.999
1
0.164
0.965
0.87
0.999
0.881
0.778
1
0.869
0.918
1
0.735
0.986
0.99
0.51
0.826
Glyco_hydro_38
Alpha-mann_mid
Glyco_hydro_38C
