# Supplementary material for: Diversity of Cell Wall Related Proteins in Human Pathogenic Fungi
Source: J Fungi (Basel). 2017 Dec 29;4(1):6. doi: 10.3390/jof4010006 (PMC5872309; doi:10.3390/jof4010006)
Supplement: Supplementary file 1 [file jof-04-00006-s001.zip › jof-252817-final-supplementary materials/Dataset S3_trees_pdf/GH55.pdf]

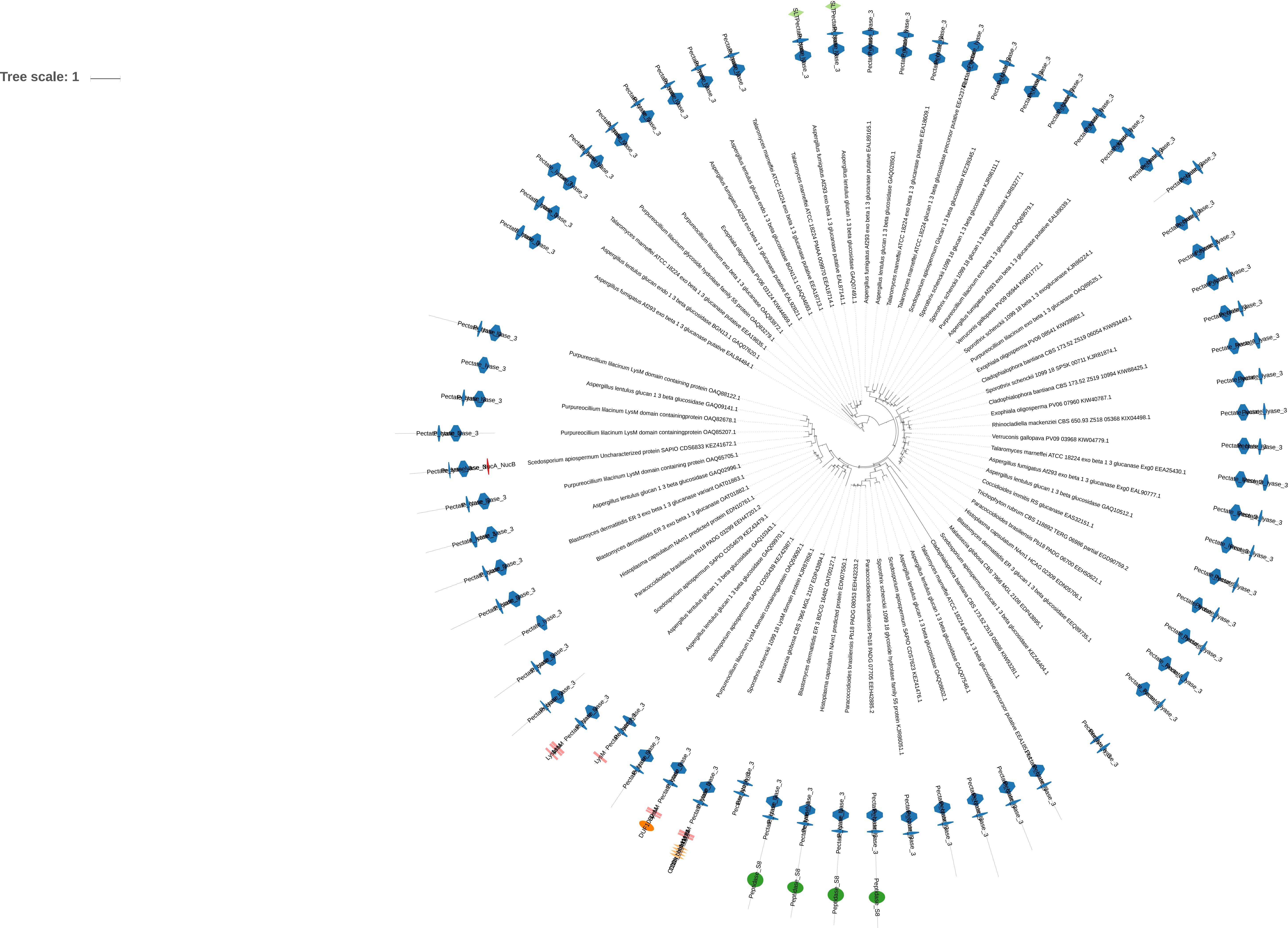

Tree scale: 1
Aspergillus lentulus glucan 1 3 beta glucosidase GAQ07491.1
Aspergillus fumigatus Af293 exo beta 1 3 glucanase putative EAL89165.1
Aspergillus lentulus glucan 1 3 beta glucosidase GAQ02850.1
Talaromyces marneffei ATCC 18224 exo beta 1 3 glucanase putative EEA18609.1
Talaromyces marneffei ATCC 18224 glucan 1 3 beta glucosidase precursor putative EEA23743.1
Scedosporium apiospermum Glucan 1 3 beta glucosidase KEZ39345.1
Sporothrix schenckii 1099 18 glucan 1 3 beta glucosidase KJR86111.1
Sporothrix schenckii 1099 18 glucan 1 3 beta glucosidase KJR83277.1
Purpureocillium lilacinum exo beta 1 3 glucanase OAQ69579.1
Aspergillus fumigatus Af293 exo beta 1 3 glucanase putative EAL89039.1
Verruconis gallopava PV09 06944 KIW01772.1
Sporothrix schenckii 1099 18 beta 1 3 exoglucanase KJR86224.1
Purpureocillium lilacinum exo beta 1 3 glucanase OAQ89525.1
Exophiala oligosperma PV06 08541 KIW39982.1
Cladophialophora bantiana CBS 173.52 Z519 06054 KIW93449.1
Sporothrix schenckii 1099 18 SPSK 00711 KJR81874.1
Cladophialophora bantiana CBS 173.52 Z519 10994 KIW88425.1
Exophiala oligosperma PV06 07960 KIW40787.1
Rhinocladiella mackenziei CBS 650.93 Z518 05368 KIX04498.1
Verruconis gallopava PV09 03968 KIW04779.1
Talaromyces marneffei ATCC 18224 exo beta 1 3 glucanase Exg0 EEA25430.1
Aspergillus fumigatus Af293 exo beta 1 3 glucanase Exg0 EAL90777.1
Aspergillus lentulus glucan 1 3 beta glucosidase GAQ10512.1
Coccidioides immitis RS glucanase EAS32151.1
Trichophyton rubrum CBS 118892 TERG 06986 partial EGD90759.2
Paracoccidioides brasiliensis Pb18 PADG 06700 EEH50621.1
Histoplasma capsulatum NAm1 HCAG 02309 EDN05706.1
Blastomyces dermatitidis ER 3 glucan 1 3 beta glucosidase EEQ89735.1
Malassezia globosa CBS 7966 MGL 2108 EDP43895.1
Scedosporium apiospermum Glucan 1 3 beta glucosidase KEZ46404.1
Cladophialophora bantiana CBS 173.52 Z519 05886 KIW93281.1
Talaromyces marneffei ATCC 18224 glucan 1 3 beta glucosidase precursor putative EEA18...
Aspergillus lentulus glucan 1 3 beta glucosidase GAQ07546.1
Aspergillus lentulus glucan 1 3 beta glucosidase GAQ08602.1
Scedosporium apiospermum SAPIO CDS7623 KEZ41476.1
Sporothrix schenckii 1099 18 glycoside hydrolase family 55 protein KJR86051.1
Paracoccidioides brasiliensis Pb18 PADG 08053 EEH42885.2
Paracoccidioides brasiliensis Pb18 PADG 07705 EEH42885.2
Histoplasma capsulatum NAm1 predicted protein EDN07550.1
Blastomyces dermatitidis ER 3 BDCG 16482 OAT00127.1
Malassezia globosa CBS 7966 MGL 2107 EDP43894.1
Sporothrix schenckii 1099 18 LysM domain protein KJR87858.1
Purpureocillium lilacinum LysM domain containingprotein OAQ58092.1
Scedosporium apiospermum SAPIO CDS5439 KEZ42987.1
Aspergillus lentulus glucan 1 3 beta glucosidase GAQ09970.1
Aspergillus lentulus glucan 1 3 beta glucosidase GAQ10343.1
Scedosporium apiospermum SAPIO CDS4679 KEZ43479.1
Paracoccidioides brasiliensis Pb18 PADG 03299 EEH47201.2
Histoplasma capsulatum NAm1 predicted protein EDN10761.1
Blastomyces dermatitidis ER 3 exo beta 1 3 glucanase OAT01882.1
Blastomyces dermatitidis ER 3 exo beta 1 3 glucanase variant OAT01883.1
Aspergillus lentulus glucan 1 3 beta glucosidase GAQ02996.1
Purpureocillium lilacinum LysM domain containing protein OAQ65705.1
Scedosporium apiospermum Uncharacterized protein SAPIO CDS6833 KEZ41672.1
Purpureocillium lilacinum LysM domain containingprotein OAQ85207.1
Purpureocillium lilacinum LysM domain containingprotein OAQ82678.1
Aspergillus lentulus glucan 1 3 beta glucosidase GAQ09141.1
Purpureocillium lilacinum LysM domain containing protein OAQ88122.1
Aspergillus fumigatus Af293 exo beta 1 3 glucanase putative EAL84484.1
Aspergillus lentulus glucan endo 1 3 beta glucosidase BGN13.1 GAQ07620.1
Talaromyces marneffei ATCC 18224 exo beta 1 3 glucanase putative EEA19835.1
Purpureocillium lilacinum glycoside hydrolase family 55 protein OAQ83279.1
Purpureocillium lilacinum exo beta 1 3 glucanase OAQ93872.1
Exophiala oligosperma PV06 03124 KIW44689.1
Aspergillus fumigatus Af293 exo beta 1 3 glucanase putative EAL92821.1
Aspergillus lentulus glucan endo 1 3 beta glucosidase BGN13.1 GAQ04693.1
Talaromyces marneffei ATCC 18224 exo beta 1 3 glucanase putative EEA18713.1
Talaromyces marneffei ATCC 18224 PMAA 009970 EEA18714.1
Aspergillus fumigatus Af293 exo beta 1 3 glucanase putative EAL87141.1
Pectate_lyase_3
SLT
LysM
NucA_NucB
DUF
Peptidase_S8
