# Supplementary material for: Diversity of Cell Wall Related Proteins in Human Pathogenic Fungi
Source: J Fungi (Basel). 2017 Dec 29;4(1):6. doi: 10.3390/jof4010006 (PMC5872309; doi:10.3390/jof4010006)
Supplement: Supplementary file 1 [file jof-04-00006-s001.zip › jof-252817-final-supplementary materials/Dataset S3_trees_pdf/Gh63.pdf]

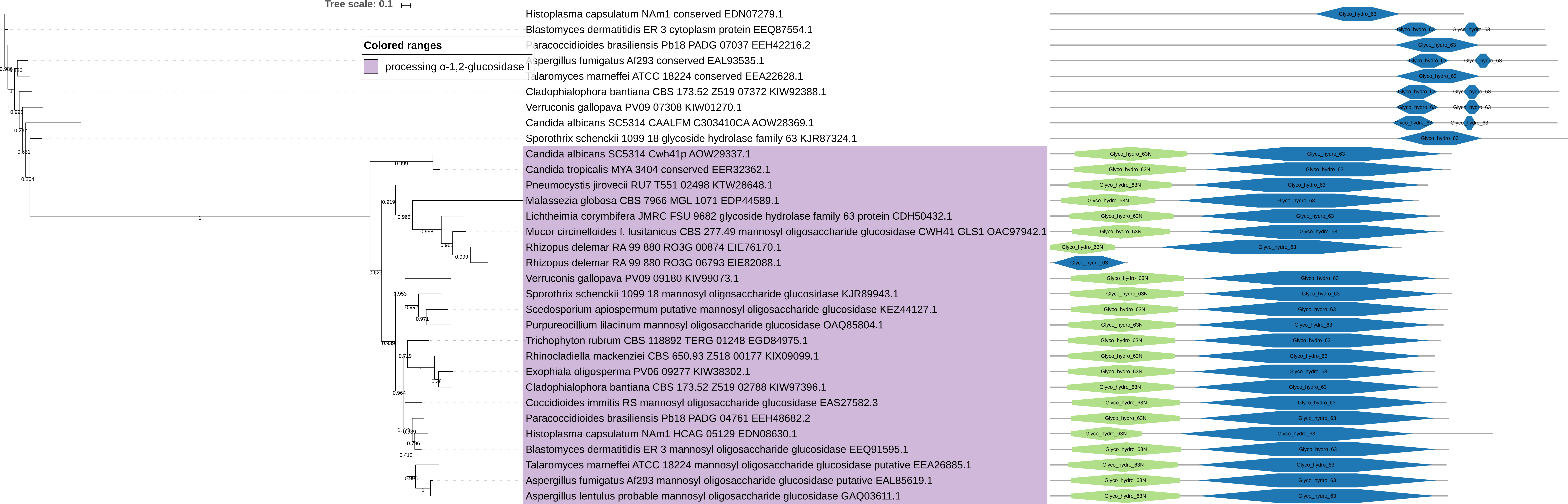

Tree scale: 0.1
Colored ranges
processing α-1,2-glucosidase I
Histoplasma capsulatum NAm1 conserved EDN07279.1
Blastomyces dermatitidis ER 3 cytoplasm protein EEQ87554.1
Paracoccidioides brasiliensis Pb18 PADG 07037 EEH42216.2
spergillus fumigatus Af293 conserved EAL93535.1
Talaromyces marneffei ATCC 18224 conserved EEA22628.1
Cladophialophora bantiana CBS 173.52 Z519 07372 KIW92388.1
Verruconis gallopava PV09 07308 KIW01270.1
Candida albicans SC5314 CAALFM C303410CA AOW28369.1
Sporothrix schenckii 1099 18 glycoside hydrolase family 63 KJR87324.1
Candida albicans SC5314 Cwh41p AOW29337.1
Candida tropicalis MYA 3404 conserved EER32362.1
Pneumocystis jirovecii RU7 T551 02498 KTW28648.1
Malassezia globosa CBS 7966 MGL 1071 EDP44589.1
Lichtheimia corymbifera JMRC FSU 9682 glycoside hydrolase family 63 protein CDH50432.1
Mucor circinelloides f. lusitanicus CBS 277.49 mannosyl oligosaccharide glucosidase CWH41 GLS1 OAC97942.1
Rhizopus delemar RA 99 880 RO3G 00874 EIE76170.1
Rhizopus delemar RA 99 880 RO3G 06793 EIE82088.1
Verruconis gallopava PV09 09180 KIV99073.1
Sporothrix schenckii 1099 18 mannosyl oligosaccharide glucosidase KJR89943.1
Scedosporium apiospermum putative mannosyl oligosaccharide glucosidase KEZ44127.1
Purpureocillium lilacinum mannosyl oligosaccharide glucosidase OAQ85804.1
Trichophyton rubrum CBS 118892 TERG 01248 EGD84975.1
Rhinocladiella mackenziei CBS 650.93 Z518 00177 KIX09099.1
Exophiala oligosperma PV06 09277 KIW38302.1
Cladophialophora bantiana CBS 173.52 Z519 02788 KIW97396.1
Coccidioides immitis RS mannosyl oligosaccharide glucosidase EAS27582.3
Paracoccidioides brasiliensis Pb18 PADG 04761 EEH48682.2
Histoplasma capsulatum NAm1 HCAG 05129 EDN08630.1
Blastomyces dermatitidis ER 3 mannosyl oligosaccharide glucosidase EEQ91595.1
Talaromyces marneffei ATCC 18224 mannosyl oligosaccharide glucosidase putative EEA26885.1
Aspergillus fumigatus Af293 mannosyl oligosaccharide glucosidase putative EAL85619.1
Aspergillus lentulus probable mannosyl oligosaccharide glucosidase GAQ03611.1
Glyco_hydro_63N
Glyco_hydro_63
