# Supplementary material for: Diversity of Cell Wall Related Proteins in Human Pathogenic Fungi
Source: J Fungi (Basel). 2017 Dec 29;4(1):6. doi: 10.3390/jof4010006 (PMC5872309; doi:10.3390/jof4010006)
Supplement: Supplementary file 1 [file jof-04-00006-s001.zip › jof-252817-final-supplementary materials/Dataset S3_trees_pdf/GH64.pdf]

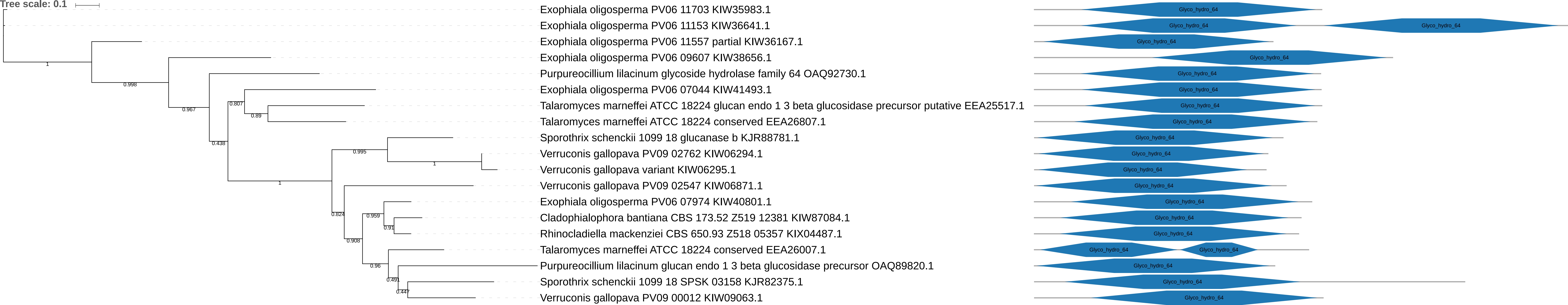
Tree scale: 0.1
Exophiala oligosperma PV06 11703 KIW35983.1
Exophiala oligosperma PV06 11153 KIW36641.1
Exophiala oligosperma PV06 11557 partial KIW36167.1
Exophiala oligosperma PV06 09607 KIW38656.1
Purpureocillium lilacinum glycoside hydrolase family 64 OAQ92730.1
Exophiala oligosperma PV06 07044 KIW41493.1
Talaromyces marneffei ATCC 18224 glucan endo 1 3 beta glucosidase precursor putative EEA25517.1
Talaromyces marneffei ATCC 18224 conserved EEA26807.1
Sporothrix schenckii 1099 18 glucanase b KJR88781.1
Verruconis gallopava PV09 02762 KIW06294.1
Verruconis gallopava variant KIW06295.1
Verruconis gallopava PV09 02547 KIW06871.1
Exophiala oligosperma PV06 07974 KIW40801.1
Cladophialophora bantiana CBS 173.52 Z519 12381 KIW87084.1
Rhinocladiella mackenziei CBS 650.93 Z518 05357 KIX04487.1
Talaromyces marneffei ATCC 18224 conserved EEA26007.1
Purpureocillium lilacinum glucan endo 1 3 beta glucosidase precursor OAQ89820.1
Sporothrix schenckii 1099 18 SPSK 03158 KJR82375.1
Verruconis gallopava PV09 00012 KIW09063.1
1
0.998
0.967
0.807
0.89
0.438
0.995
1
1
0.824
0.959
0.91
0.908
0.96
0.491
0.447
Glyco_hydro_64
