# Supplementary material for: Diversity of Cell Wall Related Proteins in Human Pathogenic Fungi
Source: J Fungi (Basel). 2017 Dec 29;4(1):6. doi: 10.3390/jof4010006 (PMC5872309; doi:10.3390/jof4010006)
Supplement: Supplementary file 1 [file jof-04-00006-s001.zip › jof-252817-final-supplementary materials/Dataset S3_trees_pdf/GH71.pdf]

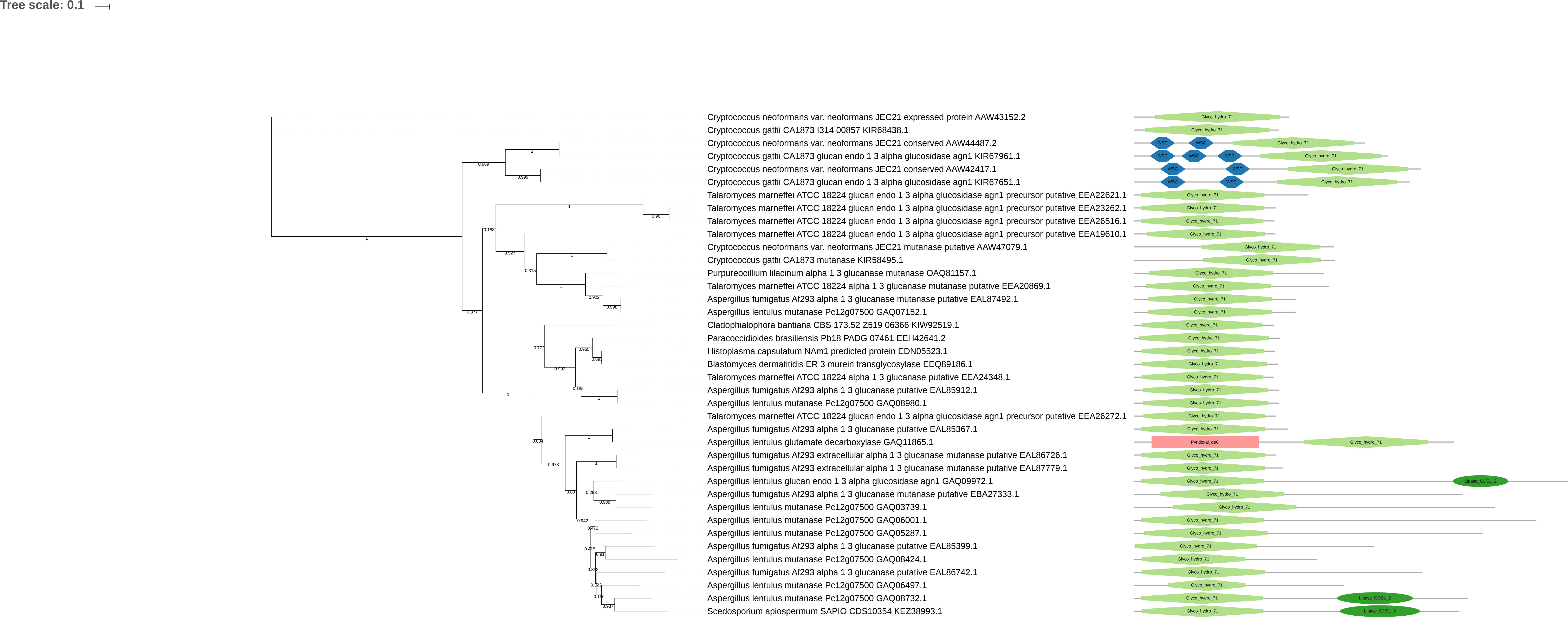
Tree scale: 0.1
Cryptococcus neoformans var. neoformans JEC21 expressed protein AAW43152.2
Cryptococcus gattii CA1873 I314 00857 KIR68438.1
Cryptococcus neoformans var. neoformans JEC21 conserved AAW44487.2
Cryptococcus gattii CA1873 glucan endo 1 3 alpha glucosidase agn1 KIR67961.1
Cryptococcus neoformans var. neoformans JEC21 conserved AAW42417.1
Cryptococcus gattii CA1873 glucan endo 1 3 alpha glucosidase agn1 KIR67651.1
Talaromyces marneffei ATCC 18224 glucan endo 1 3 alpha glucosidase agn1 precursor putative EEA22621.1
Talaromyces marneffei ATCC 18224 glucan endo 1 3 alpha glucosidase agn1 precursor putative EEA23262.1
Talaromyces marneffei ATCC 18224 glucan endo 1 3 alpha glucosidase agn1 precursor putative EEA26516.1
Talaromyces marneffei ATCC 18224 glucan endo 1 3 alpha glucosidase agn1 precursor putative EEA19610.1
Cryptococcus neoformans var. neoformans JEC21 mutanase putative AAW47079.1
Cryptococcus gattii CA1873 mutanase KIR58495.1
Purpureocillium lilacinum alpha 1 3 glucanase mutanase OAQ81157.1
Talaromyces marneffei ATCC 18224 alpha 1 3 glucanase mutanase putative EEA20869.1
Aspergillus fumigatus Af293 alpha 1 3 glucanase mutanase putative EAL87492.1
Aspergillus lentulus mutanase Pc12g07500 GAQ07152.1
Cladophialophora bantiana CBS 173.52 Z519 06366 KIW92519.1
Paracoccidioides brasiliensis Pb18 PADG 07461 EEH42641.2
Histoplasma capsulatum NAm1 predicted protein EDN05523.1
Blastomyces dermatitidis ER 3 murein transglycosylase EEQ89186.1
Talaromyces marneffei ATCC 18224 alpha 1 3 glucanase putative EEA24348.1
Aspergillus fumigatus Af293 alpha 1 3 glucanase putative EAL85912.1
Aspergillus lentulus mutanase Pc12g07500 GAQ08980.1
Talaromyces marneffei ATCC 18224 glucan endo 1 3 alpha glucosidase agn1 precursor putative EEA26272.1
Aspergillus fumigatus Af293 alpha 1 3 glucanase putative EAL85367.1
Aspergillus lentulus glutamate decarboxylase GAQ11865.1
Aspergillus fumigatus Af293 extracellular alpha 1 3 glucanase mutanase putative EAL86726.1
Aspergillus fumigatus Af293 extracellular alpha 1 3 glucanase mutanase putative EAL87779.1
Aspergillus lentulus glucan endo 1 3 alpha glucosidase agn1 GAQ09972.1
Aspergillus fumigatus Af293 alpha 1 3 glucanase mutanase putative EBA27333.1
Aspergillus lentulus mutanase Pc12g07500 GAQ03739.1
Aspergillus lentulus mutanase Pc12g07500 GAQ06001.1
Aspergillus lentulus mutanase Pc12g07500 GAQ05287.1
Aspergillus fumigatus Af293 alpha 1 3 glucanase putative EAL85399.1
Aspergillus lentulus mutanase Pc12g07500 GAQ08424.1
Aspergillus fumigatus Af293 alpha 1 3 glucanase putative EAL86742.1
Aspergillus lentulus mutanase Pc12g07500 GAQ06497.1
Aspergillus lentulus mutanase Pc12g07500 GAQ08732.1
Scedosporium apiospermum SAPIO CDS10354 KEZ38993.1
Glyco_hydro_71
WSC
Pyridoxal_deC
Lipase_GDSL_2
