# Supplementary material for: Diversity of Cell Wall Related Proteins in Human Pathogenic Fungi
Source: J Fungi (Basel). 2017 Dec 29;4(1):6. doi: 10.3390/jof4010006 (PMC5872309; doi:10.3390/jof4010006)
Supplement: Supplementary file 1 [file jof-04-00006-s001.zip › jof-252817-final-supplementary materials/Dataset S3_trees_pdf/GH75.pdf]

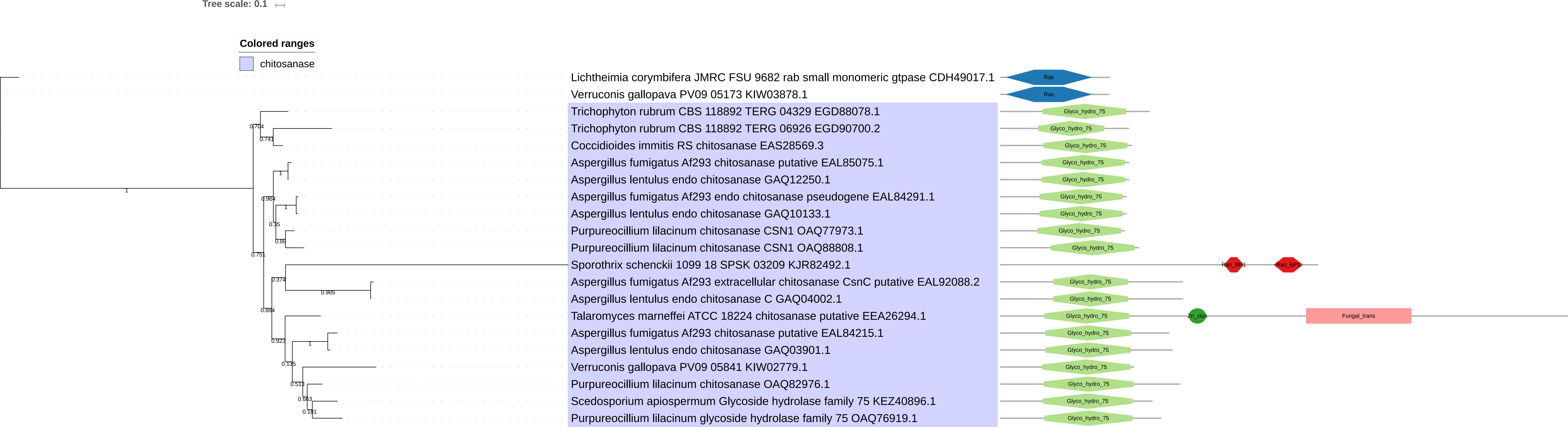

Tree scale: 0.1
Colored ranges
chitosanase
Lichtheimia corymbifera JMRC FSU 9682 rab small monomeric gtpase CDH49017.1
Verruconis gallopava PV09 05173 KIW03878.1
Trichophyton rubrum CBS 118892 TERG 04329 EGD88078.1
Trichophyton rubrum CBS 118892 TERG 06926 EGD90700.2
Coccidioides immitis RS chitosanase EAS28569.3
Aspergillus fumigatus Af293 chitosanase putative EAL85075.1
Aspergillus lentulus endo chitosanase GAQ12250.1
Aspergillus fumigatus Af293 endo chitosanase pseudogene EAL84291.1
Aspergillus lentulus endo chitosanase GAQ10133.1
Purpureocillium lilacinum chitosanase CSN1 OAQ77973.1
Purpureocillium lilacinum chitosanase CSN1 OAQ88808.1
Sporothrix schenckii 1099 18 SPSK 03209 KJR82492.1
Aspergillus fumigatus Af293 extracellular chitosanase CsnC putative EAL92088.2
Aspergillus lentulus endo chitosanase C GAQ04002.1
Talaromyces marneffei ATCC 18224 chitosanase putative EEA26294.1
Aspergillus fumigatus Af293 chitosanase putative EAL84215.1
Aspergillus lentulus endo chitosanase GAQ03901.1
Verruconis gallopava PV09 05841 KIW02779.1
Purpureocillium lilacinum chitosanase OAQ82976.1
Scedosporium apiospermum Glycoside hydrolase family 75 KEZ40896.1
Purpureocillium lilacinum glycoside hydrolase family 75 OAQ76919.1
1
0.704
0.741
1
0.964
1
0.35
0.88
0.751
0.374
0.985
0.884
0.923
1
0.135
0.513
0.663
0.181
Ras
Ras
Glyco_hydro_75
Ran_BP1
Ran_BP1
Zn_clus
Fungal_trans
