# Supplementary material for: Diversity of Cell Wall Related Proteins in Human Pathogenic Fungi
Source: J Fungi (Basel). 2017 Dec 29;4(1):6. doi: 10.3390/jof4010006 (PMC5872309; doi:10.3390/jof4010006)
Supplement: Supplementary file 1 [file jof-04-00006-s001.zip › jof-252817-final-supplementary materials/Dataset S3_trees_pdf/GH81.pdf]

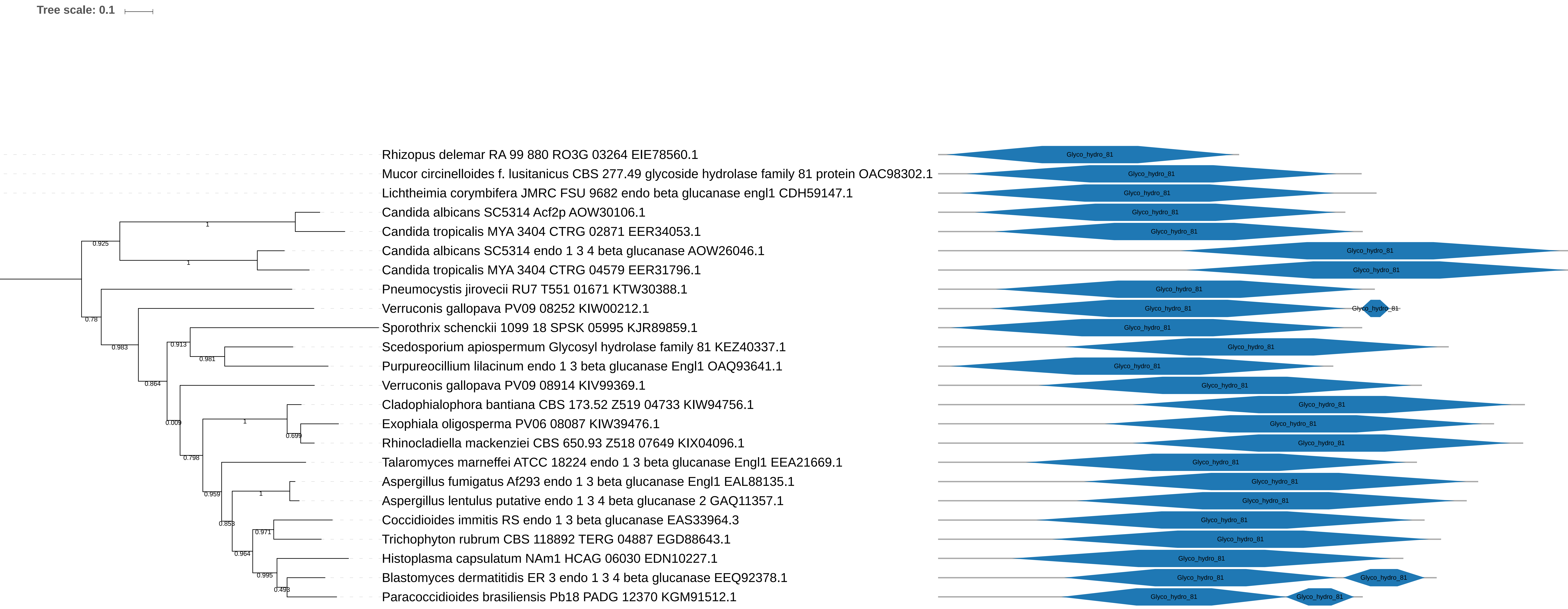
Tree scale: 0.1
Rhizopus delemar RA 99 880 RO3G 03264 EIE78560.1
Mucor circinelloides f. lusitanicus CBS 277.49 glycoside hydrolase family 81 protein OAC98302.1
Lichtheimia corymbifera JMRC FSU 9682 endo beta glucanase engl1 CDH59147.1
Candida albicans SC5314 Acf2p AOW30106.1
Candida tropicalis MYA 3404 CTRG 02871 EER34053.1
Candida albicans SC5314 endo 1 3 4 beta glucanase AOW26046.1
Candida tropicalis MYA 3404 CTRG 04579 EER31796.1
Pneumocystis jirovecii RU7 T551 01671 KTW30388.1
Verruconis gallopava PV09 08252 KIW00212.1
Sporothrix schenckii 1099 18 SPSK 05995 KJR89859.1
Scedosporium apiospermum Glycosyl hydrolase family 81 KEZ40337.1
Purpureocillium lilacinum endo 1 3 beta glucanase Engl1 OAQ93641.1
Verruconis gallopava PV09 08914 KIV99369.1
Cladophialophora bantiana CBS 173.52 Z519 04733 KIW94756.1
Exophiala oligosperma PV06 08087 KIW39476.1
Rhinocladiella mackenziei CBS 650.93 Z518 07649 KIX04096.1
Talaromyces marneffei ATCC 18224 endo 1 3 beta glucanase Engl1 EEA21669.1
Aspergillus fumigatus Af293 endo 1 3 beta glucanase Engl1 EAL88135.1
Aspergillus lentulus putative endo 1 3 4 beta glucanase 2 GAQ11357.1
Coccidioides immitis RS endo 1 3 beta glucanase EAS33964.3
Trichophyton rubrum CBS 118892 TERG 04887 EGD88643.1
Histoplasma capsulatum NAm1 HCAG 06030 EDN10227.1
Blastomyces dermatitidis ER 3 endo 1 3 4 beta glucanase EEQ92378.1
Paracoccidioides brasiliensis Pb18 PADG 12370 KGM91512.1
1
0.925
1
0.78
0.983
0.913
0.981
0.864
0.009
1
0.699
0.798
0.959
1
0.853
0.971
0.964
0.995
0.493
Glyco_hydro_81
