# Supplementary material for: Diversity of Cell Wall Related Proteins in Human Pathogenic Fungi
Source: J Fungi (Basel). 2017 Dec 29;4(1):6. doi: 10.3390/jof4010006 (PMC5872309; doi:10.3390/jof4010006)
Supplement: Supplementary file 1 [file jof-04-00006-s001.zip › jof-252817-final-supplementary materials/Dataset S3_trees_pdf/GH92.pdf]

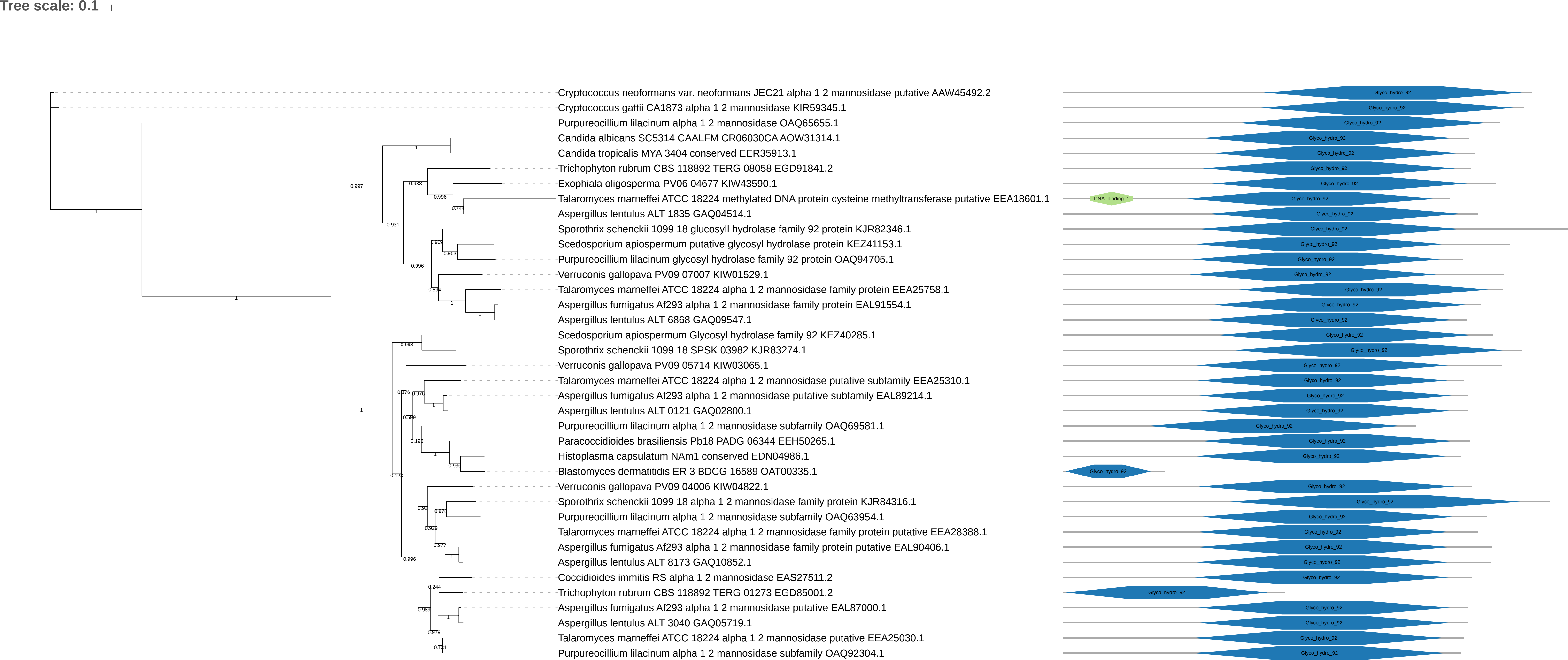
Tree scale: 0.1
Cryptococcus neoformans var. neoformans JEC21 alpha 1 2 mannosidase putative AAW45492.2
Cryptococcus gattii CA1873 alpha 1 2 mannosidase KIR59345.1
Purpureocillium lilacinum alpha 1 2 mannosidase OAQ65655.1
Candida albicans SC5314 CAALFM CR06030CA AOW31314.1
Candida tropicalis MYA 3404 conserved EER35913.1
Trichophyton rubrum CBS 118892 TERG 08058 EGD91841.2
Exophiala oligosperma PV06 04677 KIW43590.1
Talaromyces marneffei ATCC 18224 methylated DNA protein cysteine methyltransferase putative EEA18601.1
Aspergillus lentulus ALT 1835 GAQ04514.1
Sporothrix schenckii 1099 18 glucosyll hydrolase family 92 protein KJR82346.1
Scedosporium apiospermum putative glycosyl hydrolase protein KEZ41153.1
Purpureocillium lilacinum glycosyl hydrolase family 92 protein OAQ94705.1
Verruconis gallopava PV09 07007 KIW01529.1
Talaromyces marneffei ATCC 18224 alpha 1 2 mannosidase family protein EEA25758.1
Aspergillus fumigatus Af293 alpha 1 2 mannosidase family protein EAL91554.1
Aspergillus lentulus ALT 6868 GAQ09547.1
Scedosporium apiospermum Glycosyl hydrolase family 92 KEZ40285.1
Sporothrix schenckii 1099 18 SPSK 03982 KJR83274.1
Verruconis gallopava PV09 05714 KIW03065.1
Talaromyces marneffei ATCC 18224 alpha 1 2 mannosidase putative subfamily EEA25310.1
Aspergillus fumigatus Af293 alpha 1 2 mannosidase putative subfamily EAL89214.1
Aspergillus lentulus ALT 0121 GAQ02800.1
Purpureocillium lilacinum alpha 1 2 mannosidase subfamily OAQ69581.1
Paracoccidioides brasiliensis Pb18 PADG 06344 EEH50265.1
Histoplasma capsulatum NAm1 conserved EDN04986.1
Blastomyces dermatitidis ER 3 BDCG 16589 OAT00335.1
Verruconis gallopava PV09 04006 KIW04822.1
Sporothrix schenckii 1099 18 alpha 1 2 mannosidase family protein KJR84316.1
Purpureocillium lilacinum alpha 1 2 mannosidase subfamily OAQ63954.1
Talaromyces marneffei ATCC 18224 alpha 1 2 mannosidase family protein putative EEA28388.1
Aspergillus fumigatus Af293 alpha 1 2 mannosidase family protein putative EAL90406.1
Aspergillus lentulus ALT 8173 GAQ10852.1
Coccidioides immitis RS alpha 1 2 mannosidase EAS27511.2
Trichophyton rubrum CBS 118892 TERG 01273 EGD85001.2
Aspergillus fumigatus Af293 alpha 1 2 mannosidase putative EAL87000.1
Aspergillus lentulus ALT 3040 GAQ05719.1
Talaromyces marneffei ATCC 18224 alpha 1 2 mannosidase putative EEA25030.1
Purpureocillium lilacinum alpha 1 2 mannosidase subfamily OAQ92304.1
Glyco_hydro_92
DNA_binding_1
