# Supplementary material for: Diversity of Cell Wall Related Proteins in Human Pathogenic Fungi
Source: J Fungi (Basel). 2017 Dec 29;4(1):6. doi: 10.3390/jof4010006 (PMC5872309; doi:10.3390/jof4010006)
Supplement: Supplementary file 1 [file jof-04-00006-s001.zip › jof-252817-final-supplementary materials/Dataset S3_trees_pdf/GT10.pdf]

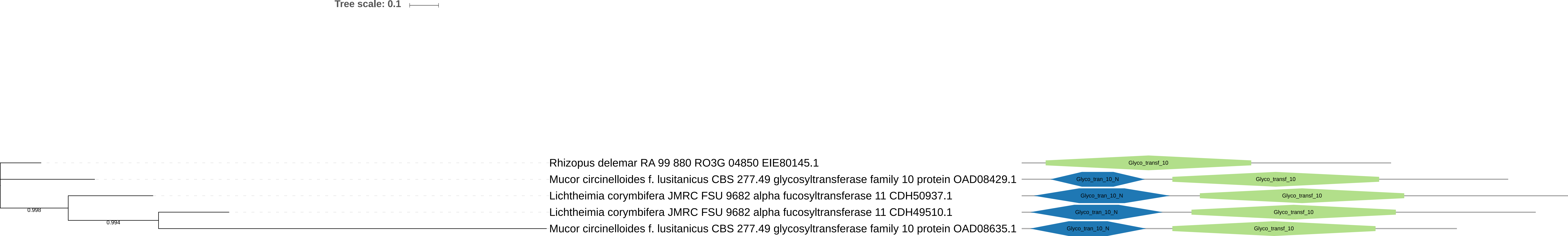

Tree scale: 0.1
Rhizopus delemar RA 99 880 RO3G 04850 EIE80145.1
Mucor circinelloides f. lusitanicus CBS 277.49 glycosyltransferase family 10 protein OAD08429.1
Lichtheimia corymbifera JMRC FSU 9682 alpha fucosyltransferase 11 CDH50937.1
Lichtheimia corymbifera JMRC FSU 9682 alpha fucosyltransferase 11 CDH49510.1
Mucor circinelloides f. lusitanicus CBS 277.49 glycosyltransferase family 10 protein OAD08635.1
0.998
0.994
Glyco_transf_10
Glyco_tran_10_N
