# Supplementary material for: Diversity of Cell Wall Related Proteins in Human Pathogenic Fungi
Source: J Fungi (Basel). 2017 Dec 29;4(1):6. doi: 10.3390/jof4010006 (PMC5872309; doi:10.3390/jof4010006)
Supplement: Supplementary file 1 [file jof-04-00006-s001.zip › jof-252817-final-supplementary materials/Dataset S3_trees_pdf/GT21.pdf]

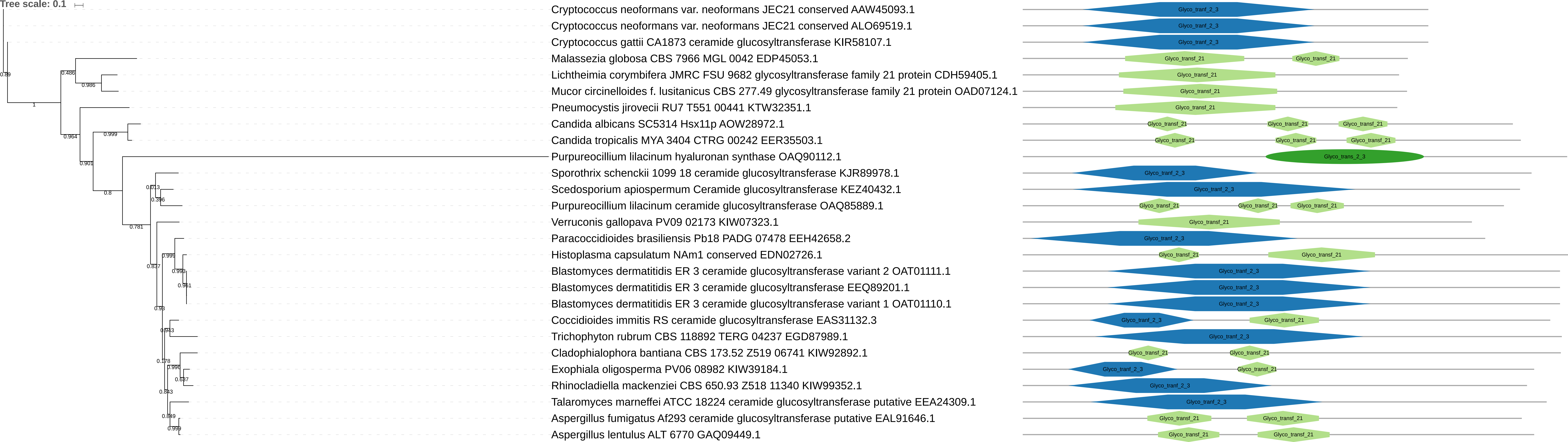
Tree scale: 0.1
Cryptococcus neoformans var. neoformans JEC21 conserved AAW45093.1
Cryptococcus neoformans var. neoformans JEC21 conserved ALO69519.1
Cryptococcus gattii CA1873 ceramide glucosyltransferase KIR58107.1
Malassezia globosa CBS 7966 MGL 0042 EDP45053.1
Lichtheimia corymbifera JMRC FSU 9682 glycosyltransferase family 21 protein CDH59405.1
Mucor circinelloides f. lusitanicus CBS 277.49 glycosyltransferase family 21 protein OAD07124.1
Pneumocystis jirovecii RU7 T551 00441 KTW32351.1
Candida albicans SC5314 Hsx11p AOW28972.1
Candida tropicalis MYA 3404 CTRG 00242 EER35503.1
Purpureocillium lilacinum hyaluronan synthase OAQ90112.1
Sporothrix schenckii 1099 18 ceramide glucosyltransferase KJR89978.1
Scedosporium apiospermum Ceramide glucosyltransferase KEZ40432.1
Purpureocillium lilacinum ceramide glucosyltransferase OAQ85889.1
Verruconis gallopava PV09 02173 KIW07323.1
Paracoccidioides brasiliensis Pb18 PADG 07478 EEH42658.2
Histoplasma capsulatum NAm1 conserved EDN02726.1
Blastomyces dermatitidis ER 3 ceramide glucosyltransferase variant 2 OAT01111.1
Blastomyces dermatitidis ER 3 ceramide glucosyltransferase EEQ89201.1
Blastomyces dermatitidis ER 3 ceramide glucosyltransferase variant 1 OAT01110.1
Coccidioides immitis RS ceramide glucosyltransferase EAS31132.3
Trichophyton rubrum CBS 118892 TERG 04237 EGD87989.1
Cladophialophora bantiana CBS 173.52 Z519 06741 KIW92892.1
Exophiala oligosperma PV06 08982 KIW39184.1
Rhinocladiella mackenziei CBS 650.93 Z518 11340 KIW99352.1
Talaromyces marneffei ATCC 18224 ceramide glucosyltransferase putative EEA24309.1
Aspergillus fumigatus Af293 ceramide glucosyltransferase putative EAL91646.1
Aspergillus lentulus ALT 6770 GAQ09449.1
Glyco_tranf_2_3
Glyco_transf_21
Glyco_trans_2_3
