# Supplementary material for: Diversity of Cell Wall Related Proteins in Human Pathogenic Fungi
Source: J Fungi (Basel). 2017 Dec 29;4(1):6. doi: 10.3390/jof4010006 (PMC5872309; doi:10.3390/jof4010006)
Supplement: Supplementary file 1 [file jof-04-00006-s001.zip › jof-252817-final-supplementary materials/Dataset S3_trees_pdf/GT22.pdf]

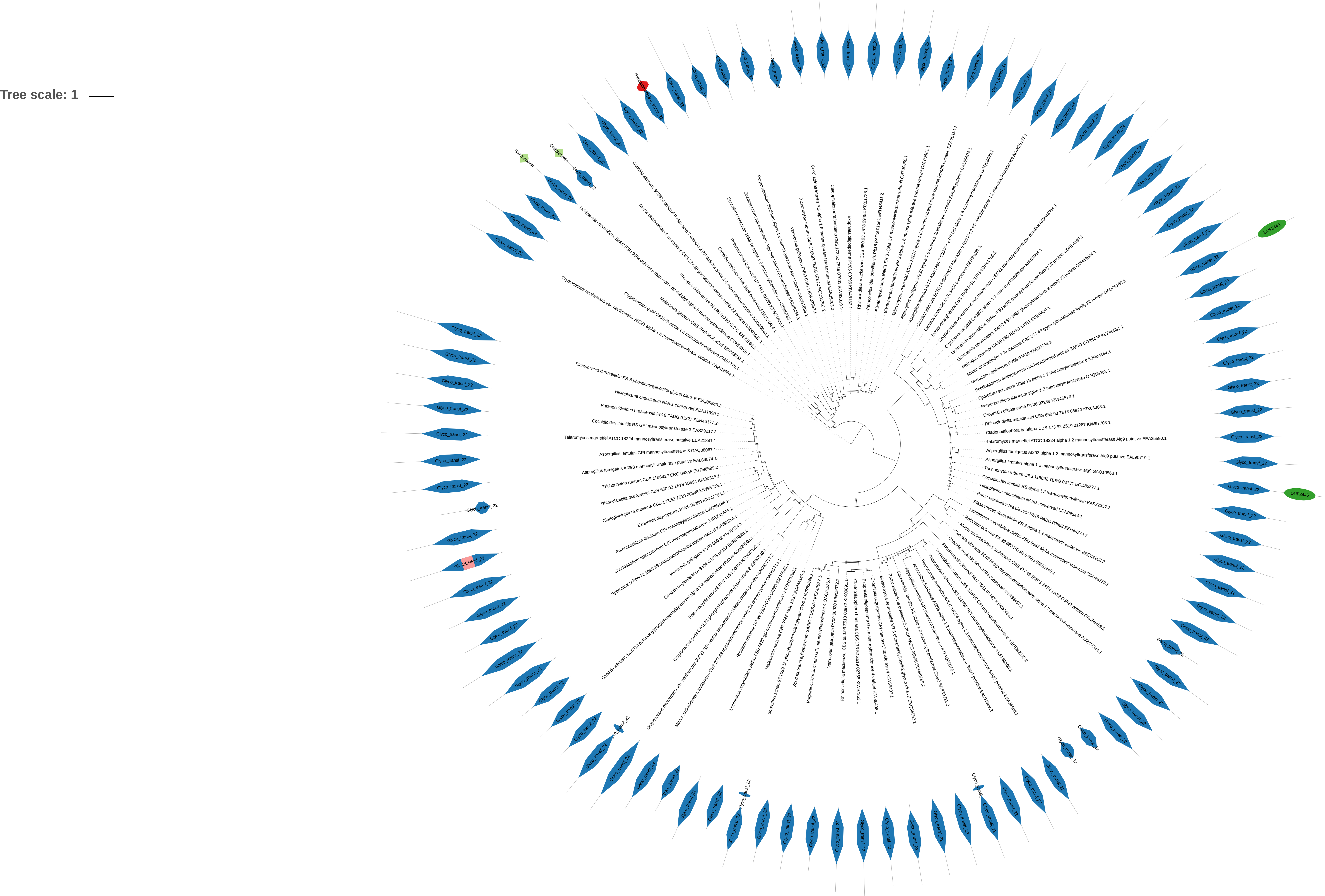

Tree scale: 1
Blastomyces dermatitidis ER 3 phosphatidylinositol glycan class B EEQ85549.2
Histoplasma capsulatum NAm1 conserved EDN11390.1
Paracoccidioides brasiliensis Pb18 PADG 01327 EEH45177.2
Coccidioides immitis RS GPI mannosyltransferase 3 EAS29217.3
Talaromyces marneffei ATCC 18224 mannosyltransferase putative EEA21841.1
Aspergillus lentulus GPI mannosyltransferase 3 GAQ08067.1
Aspergillus fumigatus Af293 mannosyltransferase putative EAL89874.1
Trichophyton rubrum CBS 118892 TERG 04845 EGD88599.2
Rhinocladiella mackenziei CBS 650.93 Z518 10454 KIX00315.1
Cladophialophora bantiana CBS 173.52 Z519 00396 KIW98733.1
Exophiala oligosperma PV06 06269 KIW42754.1
Exophiala oligosperma PV06 02239 KIW46573.1
Rhinocladiella mackenziei CBS 650.93 Z518 06920 KIX03368.1
Cladophialophora bantiana CBS 173.52 Z519 01287 KIW97703.1
Talaromyces marneffei ATCC 18224 alpha 1 2 mannosyltransferase Alg9 putative EEA25590.1
Aspergillus fumigatus Af293 alpha 1 2 mannosyltransferase Alg9 putative EAL90719.1
Aspergillus lentulus alpha 1 2 mannosyltransferase alg9 GAQ10563.1
Trichophyton rubrum CBS 118892 TERG 03131 EGD86877.1
Glyco_transf_22
DUF3445
Glutaredoxin
