# Supplementary material for: Diversity of Cell Wall Related Proteins in Human Pathogenic Fungi
Source: J Fungi (Basel). 2017 Dec 29;4(1):6. doi: 10.3390/jof4010006 (PMC5872309; doi:10.3390/jof4010006)
Supplement: Supplementary file 1 [file jof-04-00006-s001.zip › jof-252817-final-supplementary materials/Dataset S3_trees_pdf/GT24.pdf]

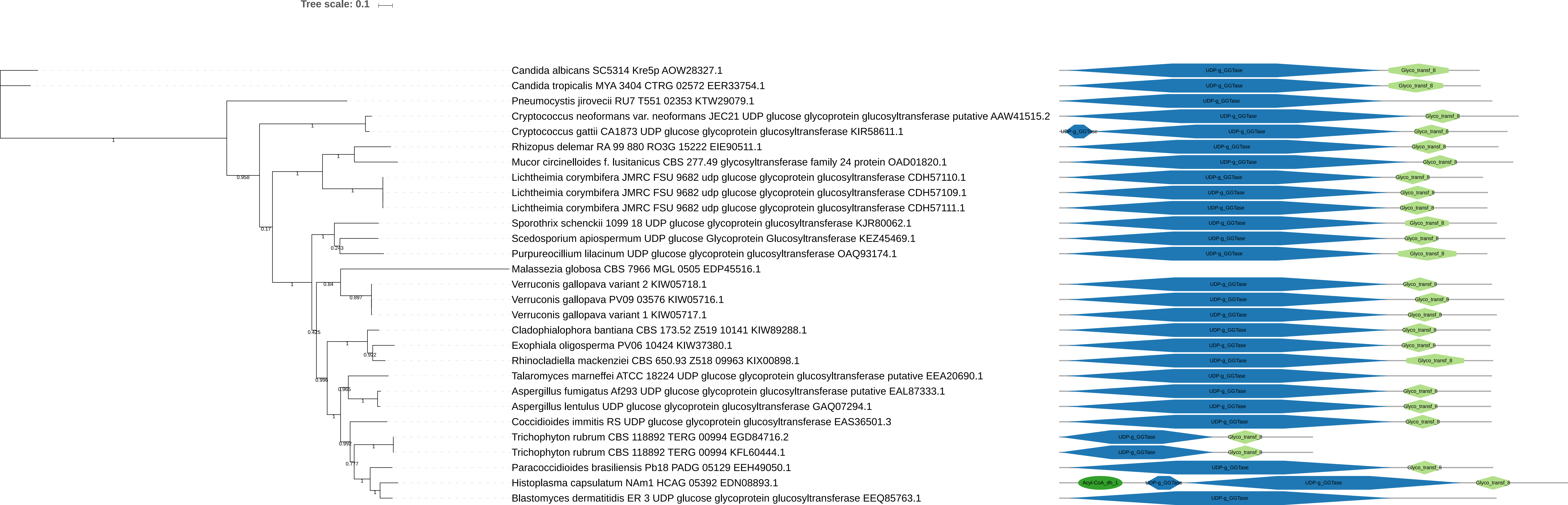
Tree scale: 0.1
Candida albicans SC5314 Kre5p AOW28327.1
Candida tropicalis MYA 3404 CTRG 02572 EER33754.1
Pneumocystis jirovecii RU7 T551 02353 KTW29079.1
Cryptococcus neoformans var. neoformans JEC21 UDP glucose glycoprotein glucosyltransferase putative AAW41515.2
Cryptococcus gattii CA1873 UDP glucose glycoprotein glucosyltransferase KIR58611.1
Rhizopus delemar RA 99 880 RO3G 15222 EIE90511.1
Mucor circinelloides f. lusitanicus CBS 277.49 glycosyltransferase family 24 protein OAD01820.1
Lichtheimia corymbifera JMRC FSU 9682 udp glucose glycoprotein glucosyltransferase CDH57110.1
Lichtheimia corymbifera JMRC FSU 9682 udp glucose glycoprotein glucosyltransferase CDH57109.1
Lichtheimia corymbifera JMRC FSU 9682 udp glucose glycoprotein glucosyltransferase CDH57111.1
Sporothrix schenckii 1099 18 UDP glucose glycoprotein glucosyltransferase KJR80062.1
Scedosporium apiospermum UDP glucose Glycoprotein Glucosyltransferase KEZ45469.1
Purpureocillium lilacinum UDP glucose glycoprotein glucosyltransferase OAQ93174.1
Malassezia globosa CBS 7966 MGL 0505 EDP45516.1
Verruconis gallopava variant 2 KIW05718.1
Verruconis gallopava PV09 03576 KIW05716.1
Verruconis gallopava variant 1 KIW05717.1
Cladophialophora bantiana CBS 173.52 Z519 10141 KIW89288.1
Exophiala oligosperma PV06 10424 KIW37380.1
Rhinocladiella mackenziei CBS 650.93 Z518 09963 KIX00898.1
Talaromyces marneffei ATCC 18224 UDP glucose glycoprotein glucosyltransferase putative EEA20690.1
Aspergillus fumigatus Af293 UDP glucose glycoprotein glucosyltransferase putative EAL87333.1
Aspergillus lentulus UDP glucose glycoprotein glucosyltransferase GAQ07294.1
Coccidioides immitis RS UDP glucose glycoprotein glucosyltransferase EAS36501.3
Trichophyton rubrum CBS 118892 TERG 00994 EGD84716.2
Trichophyton rubrum CBS 118892 TERG 00994 KFL60444.1
Paracoccidioides brasiliensis Pb18 PADG 05129 EEH49050.1
Histoplasma capsulatum NAm1 HCAG 05392 EDN08893.1
Blastomyces dermatitidis ER 3 UDP glucose glycoprotein glucosyltransferase EEQ85763.1
UDP-g_GGTase
Glyco_transf_8
Acyl-CoA_dh_1
