# Supplementary material for: Diversity of Cell Wall Related Proteins in Human Pathogenic Fungi
Source: J Fungi (Basel). 2017 Dec 29;4(1):6. doi: 10.3390/jof4010006 (PMC5872309; doi:10.3390/jof4010006)
Supplement: Supplementary file 1 [file jof-04-00006-s001.zip › jof-252817-final-supplementary materials/Dataset S3_trees_pdf/GT31.pdf]

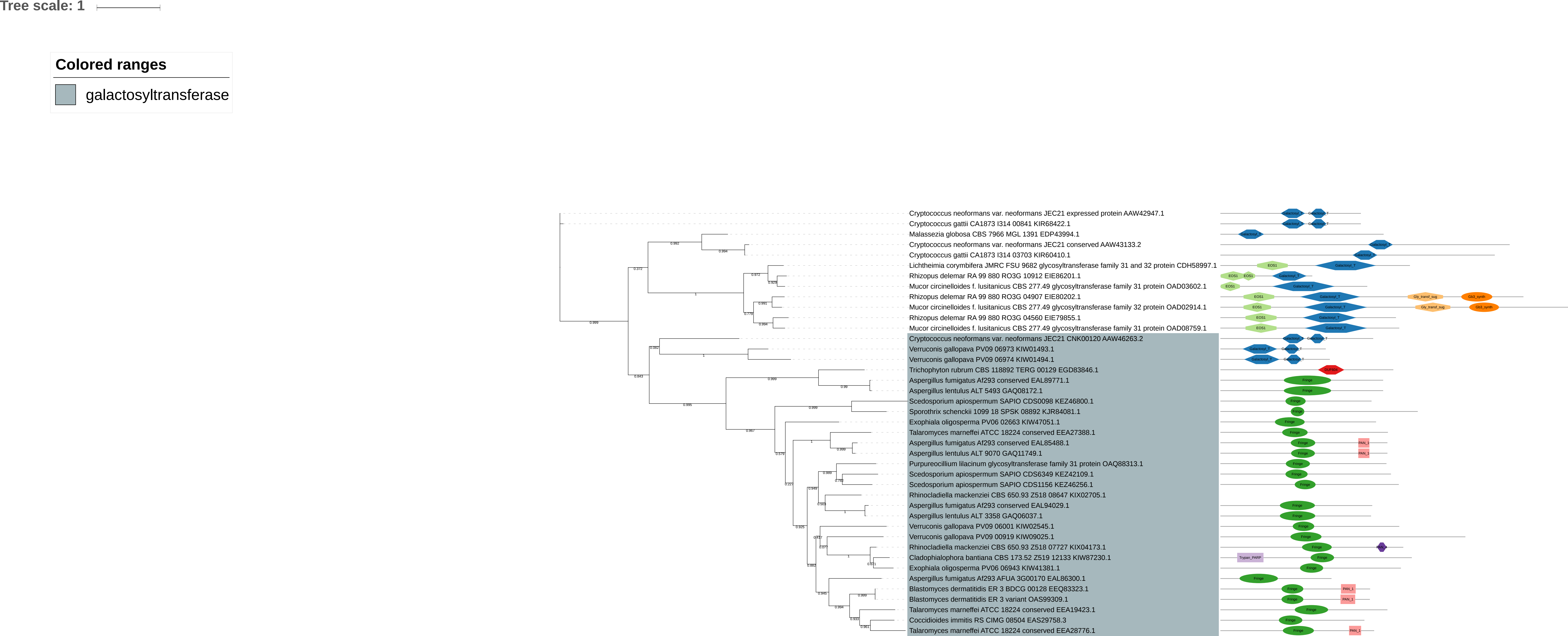

Tree scale: 1
Colored ranges
galactosyltransferase
Cryptococcus neoformans var. neoformans JEC21 expressed protein AAW42947.1
Cryptococcus gattii CA1873 I314 00841 KIR68422.1
Malassezia globosa CBS 7966 MGL 1391 EDP43994.1
Cryptococcus neoformans var. neoformans JEC21 conserved AAW43133.2
Cryptococcus gattii CA1873 I314 03703 KIR60410.1
Lichtheimia corymbifera JMRC FSU 9682 glycosyltransferase family 31 and 32 protein CDH58997.1
Rhizopus delemar RA 99 880 RO3G 10912 EIE86201.1
Mucor circinelloides f. lusitanicus CBS 277.49 glycosyltransferase family 31 protein OAD03602.1
Rhizopus delemar RA 99 880 RO3G 04907 EIE80202.1
Mucor circinelloides f. lusitanicus CBS 277.49 glycosyltransferase family 32 protein OAD02914.1
Rhizopus delemar RA 99 880 RO3G 04560 EIE79855.1
Mucor circinelloides f. lusitanicus CBS 277.49 glycosyltransferase family 31 protein OAD08759.1
Cryptococcus neoformans var. neoformans JEC21 CNK00120 AAW46263.2
Verruconis gallopava PV09 06973 KIW01493.1
Verruconis gallopava PV09 06974 KIW01494.1
Trichophyton rubrum CBS 118892 TERG 00129 EGD83846.1
Aspergillus fumigatus Af293 conserved EAL89771.1
Aspergillus lentulus ALT 5493 GAQ08172.1
Scedosporium apiospermum SAPIO CDS0098 KEZ46800.1
Sporothrix schenckii 1099 18 SPSK 08892 KJR84081.1
Exophiala oligosperma PV06 02663 KIW47051.1
Talaromyces marneffei ATCC 18224 conserved EEA27388.1
Aspergillus fumigatus Af293 conserved EAL85488.1
Aspergillus lentulus ALT 9070 GAQ11749.1
Purpureocillium lilacinum glycosyltransferase family 31 protein OAQ88313.1
Scedosporium apiospermum SAPIO CDS6349 KEZ42109.1
Scedosporium apiospermum SAPIO CDS1156 KEZ46256.1
Rhinocladiella mackenziei CBS 650.93 Z518 08647 KIX02705.1
Aspergillus fumigatus Af293 conserved EAL94029.1
Aspergillus lentulus ALT 3358 GAQ06037.1
Verruconis gallopava PV09 06001 KIW02545.1
Verruconis gallopava PV09 00919 KIW09025.1
Rhinocladiella mackenziei CBS 650.93 Z518 07727 KIX04173.1
Cladophialophora bantiana CBS 173.52 Z519 12133 KIW87230.1
Exophiala oligosperma PV06 06943 KIW41381.1
Aspergillus fumigatus Af293 AFUA 3G00170 EAL86300.1
Blastomyces dermatitidis ER 3 BDCG 00128 EEQ83323.1
Blastomyces dermatitidis ER 3 variant OAS99309.1
Talaromyces marneffei ATCC 18224 conserved EEA19423.1
Coccidioides immitis RS CIMG 08504 EAS29758.3
Talaromyces marneffei ATCC 18224 conserved EEA28776.1
EOS1
Galactosyl_T
Gly_transf_sug
Gb3_synth
DUF604
Fringe
PAN_1
Trypan_PARP
