# Supplementary material for: Diversity of Cell Wall Related Proteins in Human Pathogenic Fungi
Source: J Fungi (Basel). 2017 Dec 29;4(1):6. doi: 10.3390/jof4010006 (PMC5872309; doi:10.3390/jof4010006)
Supplement: Supplementary file 1 [file jof-04-00006-s001.zip › jof-252817-final-supplementary materials/Dataset S3_trees_pdf/GT33.pdf]

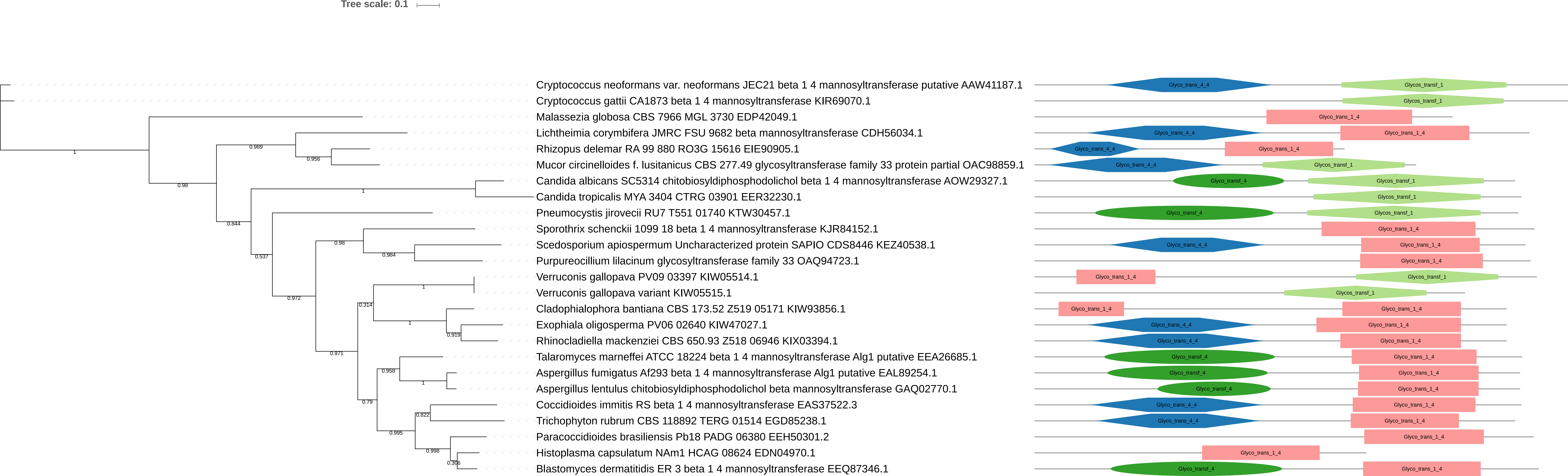
Tree scale: 0.1
Cryptococcus neoformans var. neoformans JEC21 beta 1 4 mannosyltransferase putative AAW41187.1
Cryptococcus gattii CA1873 beta 1 4 mannosyltransferase KIR69070.1
Malassezia globosa CBS 7966 MGL 3730 EDP42049.1
Lichtheimia corymbifera JMRC FSU 9682 beta mannosyltransferase CDH56034.1
Rhizopus delemar RA 99 880 RO3G 15616 EIE90905.1
Mucor circinelloides f. lusitanicus CBS 277.49 glycosyltransferase family 33 protein partial OAC98859.1
Candida albicans SC5314 chitobiosyldiphosphodolichol beta 1 4 mannosyltransferase AOW29327.1
Candida tropicalis MYA 3404 CTRG 03901 EER32230.1
Pneumocystis jirovecii RU7 T551 01740 KTW30457.1
Sporothrix schenckii 1099 18 beta 1 4 mannosyltransferase KJR84152.1
Scedosporium apiospermum Uncharacterized protein SAPIO CDS8446 KEZ40538.1
Purpureocillium lilacinum glycosyltransferase family 33 OAQ94723.1
Verruconis gallopava PV09 03397 KIW05514.1
Verruconis gallopava variant KIW05515.1
Cladophialophora bantiana CBS 173.52 Z519 05171 KIW93856.1
Exophiala oligosperma PV06 02640 KIW47027.1
Rhinocladiella mackenziei CBS 650.93 Z518 06946 KIX03394.1
Talaromyces marneffei ATCC 18224 beta 1 4 mannosyltransferase Alg1 putative EEA26685.1
Aspergillus fumigatus Af293 beta 1 4 mannosyltransferase Alg1 putative EAL89254.1
Aspergillus lentulus chitobiosyldiphosphodolichol beta mannosyltransferase GAQ02770.1
Coccidioides immitis RS beta 1 4 mannosyltransferase EAS37522.3
Trichophyton rubrum CBS 118892 TERG 01514 EGD85238.1
Paracoccidioides brasiliensis Pb18 PADG 06380 EEH50301.2
Histoplasma capsulatum NAm1 HCAG 08624 EDN04970.1
Blastomyces dermatitidis ER 3 beta 1 4 mannosyltransferase EEQ87346.1
Glyco_trans_4_4
Glycos_transf_1
Glyco_trans_1_4
Glyco_transf_4
