# Supplementary material for: Diversity of Cell Wall Related Proteins in Human Pathogenic Fungi
Source: J Fungi (Basel). 2017 Dec 29;4(1):6. doi: 10.3390/jof4010006 (PMC5872309; doi:10.3390/jof4010006)
Supplement: Supplementary file 1 [file jof-04-00006-s001.zip › jof-252817-final-supplementary materials/Dataset S3_trees_pdf/GT34.pdf]

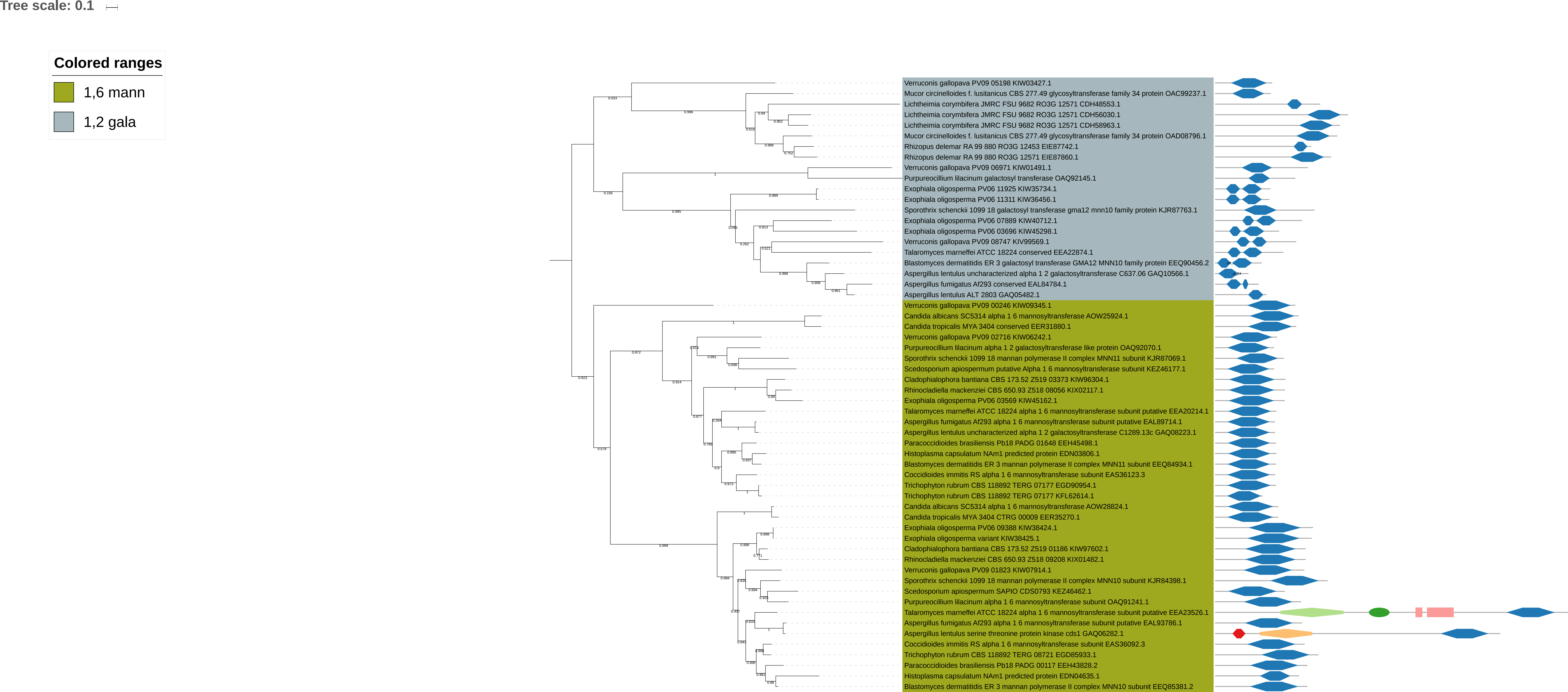
Tree scale: 0.1
Colored ranges
1,6 mann
1,2 gala
Verruconis gallopava PV09 05198 KIW03427.1
Mucor circinelloides f. lusitanicus CBS 277.49 glycosyltransferase family 34 protein OAC99237.1
Lichtheimia corymbifera JMRC FSU 9682 RO3G 12571 CDH48553.1
Lichtheimia corymbifera JMRC FSU 9682 RO3G 12571 CDH56030.1
Lichtheimia corymbifera JMRC FSU 9682 RO3G 12571 CDH58963.1
Mucor circinelloides f. lusitanicus CBS 277.49 glycosyltransferase family 34 protein OAD08796.1
Rhizopus delemar RA 99 880 RO3G 12453 EIE87742.1
Rhizopus delemar RA 99 880 RO3G 12571 EIE87860.1
Verruconis gallopava PV09 06971 KIW01491.1
Purpureocillium lilacinum galactosyl transferase OAQ92145.1
Exophiala oligosperma PV06 11925 KIW35734.1
Exophiala oligosperma PV06 11311 KIW36456.1
Sporothrix schenckii 1099 18 galactosyl transferase gma12 mnn10 family protein KJR87763.1
Exophiala oligosperma PV06 07889 KIW40712.1
Exophiala oligosperma PV06 03696 KIW45298.1
Verruconis gallopava PV09 08747 KIV99569.1
Talaromyces marneffei ATCC 18224 conserved EEA22874.1
Blastomyces dermatitidis ER 3 galactosyl transferase GMA12 MNN10 family protein EEQ90456.2
Aspergillus lentulus uncharacterized alpha 1 2 galactosyltransferase C637.06 GAQ10566.1
Aspergillus fumigatus Af293 conserved EAL84784.1
Aspergillus lentulus ALT 2803 GAQ05482.1
Verruconis gallopava PV09 00246 KIW09345.1
Candida albicans SC5314 alpha 1 6 mannosyltransferase AOW25924.1
Candida tropicalis MYA 3404 conserved EER31880.1
Verruconis gallopava PV09 02716 KIW06242.1
Purpureocillium lilacinum alpha 1 2 galactosyltransferase like protein OAQ92070.1
Sporothrix schenckii 1099 18 mannan polymerase II complex MNN11 subunit KJR87069.1
Scedosporium apiospermum putative Alpha 1 6 mannosyltransferase subunit KEZ46177.1
Cladophialophora bantiana CBS 173.52 Z519 03373 KIW96304.1
Rhinocladiella mackenziei CBS 650.93 Z518 08056 KIX02117.1
Exophiala oligosperma PV06 03569 KIW45162.1
Talaromyces marneffei ATCC 18224 alpha 1 6 mannosyltransferase subunit putative EEA20214.1
Aspergillus fumigatus Af293 alpha 1 6 mannosyltransferase subunit putative EAL89714.1
Aspergillus lentulus uncharacterized alpha 1 2 galactosyltransferase C1289.13c GAQ08223.1
Paracoccidioides brasiliensis Pb18 PADG 01648 EEH45498.1
Histoplasma capsulatum NAm1 predicted protein EDN03806.1
Blastomyces dermatitidis ER 3 mannan polymerase II complex MNN11 subunit EEQ84934.1
Coccidioides immitis RS alpha 1 6 mannosyltransferase subunit EAS36123.3
Trichophyton rubrum CBS 118892 TERG 07177 EGD90954.1
Trichophyton rubrum CBS 118892 TERG 07177 KFL62614.1
Candida albicans SC5314 alpha 1 6 mannosyltransferase AOW28824.1
Candida tropicalis MYA 3404 CTRG 00009 EER35270.1
Exophiala oligosperma PV06 09388 KIW38424.1
Exophiala oligosperma variant KIW38425.1
Cladophialophora bantiana CBS 173.52 Z519 01186 KIW97602.1
Rhinocladiella mackenziei CBS 650.93 Z518 09208 KIX01482.1
Verruconis gallopava PV09 01823 KIW07914.1
Sporothrix schenckii 1099 18 mannan polymerase II complex MNN10 subunit KJR84398.1
Scedosporium apiospermum SAPIO CDS0793 KEZ46462.1
Purpureocillium lilacinum alpha 1 6 mannosyltransferase subunit OAQ91241.1
Talaromyces marneffei ATCC 18224 alpha 1 6 mannosyltransferase subunit putative EEA23526.1
Aspergillus fumigatus Af293 alpha 1 6 mannosyltransferase subunit putative EAL93786.1
Aspergillus lentulus serine threonine protein kinase cds1 GAQ06282.1
Coccidioides immitis RS alpha 1 6 mannosyltransferase subunit EAS36092.3
Trichophyton rubrum CBS 118892 TERG 08721 EGD85933.1
Paracoccidioides brasiliensis Pb18 PADG 00117 EEH43828.2
Histoplasma capsulatum NAm1 predicted protein EDN04635.1
Blastomyces dermatitidis ER 3 mannan polymerase II complex MNN10 subunit EEQ85381.2
