# Supplementary material for: Diversity of Cell Wall Related Proteins in Human Pathogenic Fungi
Source: J Fungi (Basel). 2017 Dec 29;4(1):6. doi: 10.3390/jof4010006 (PMC5872309; doi:10.3390/jof4010006)
Supplement: Supplementary file 1 [file jof-04-00006-s001.zip › jof-252817-final-supplementary materials/Dataset S3_trees_pdf/GT47.pdf]

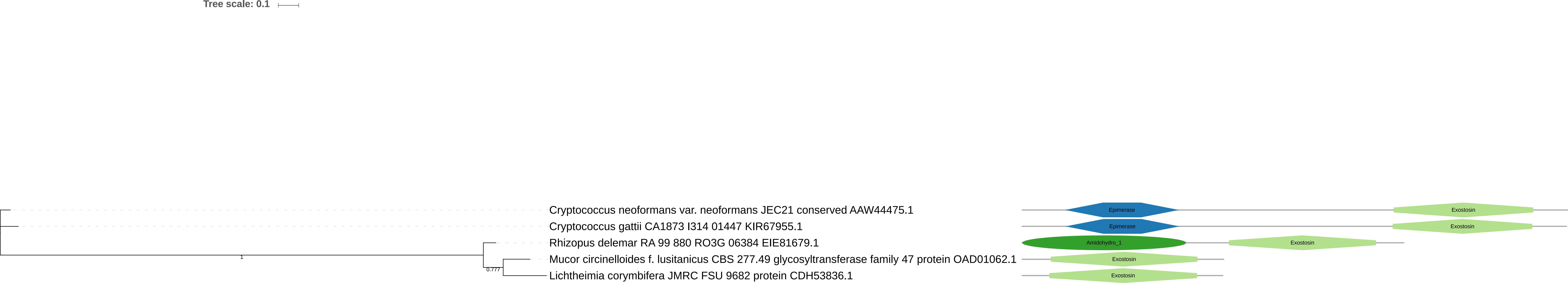
Tree scale: 0.1
Cryptococcus neoformans var. neoformans JEC21 conserved AAW44475.1
Cryptococcus gattii CA1873 I314 01447 KIR67955.1
Rhizopus delemar RA 99 880 RO3G 06384 EIE81679.1
Mucor circinelloides f. lusitanicus CBS 277.49 glycosyltransferase family 47 protein OAD01062.1
Lichtheimia corymbifera JMRC FSU 9682 protein CDH53836.1
1
0.777
Epimerase
Exostosin
Epimerase
Exostosin
Amidohydro_1
Exostosin
Exostosin
Exostosin
