# Supplementary material for: Diversity of Cell Wall Related Proteins in Human Pathogenic Fungi
Source: J Fungi (Basel). 2017 Dec 29;4(1):6. doi: 10.3390/jof4010006 (PMC5872309; doi:10.3390/jof4010006)
Supplement: Supplementary file 1 [file jof-04-00006-s001.zip › jof-252817-final-supplementary materials/Dataset S3_trees_pdf/GT48.pdf]

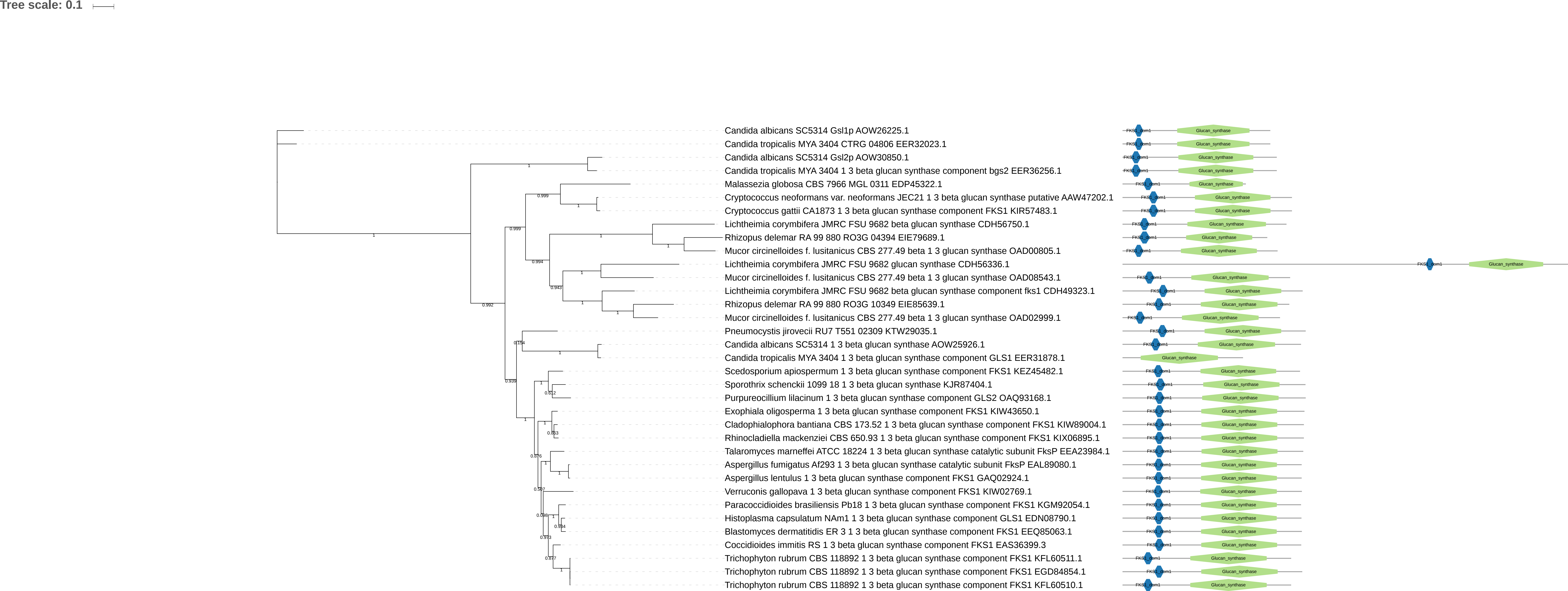
Tree scale: 0.1
Candida albicans SC5314 Gsl1p AOW26225.1
Candida tropicalis MYA 3404 CTRG 04806 EER32023.1
Candida albicans SC5314 Gsl2p AOW30850.1
Candida tropicalis MYA 3404 1 3 beta glucan synthase component bgs2 EER36256.1
Malassezia globosa CBS 7966 MGL 0311 EDP45322.1
Cryptococcus neoformans var. neoformans JEC21 1 3 beta glucan synthase putative AAW47202.1
Cryptococcus gattii CA1873 1 3 beta glucan synthase component FKS1 KIR57483.1
Lichtheimia corymbifera JMRC FSU 9682 beta glucan synthase CDH56750.1
Rhizopus delemar RA 99 880 RO3G 04394 EIE79689.1
Mucor circinelloides f. lusitanicus CBS 277.49 beta 1 3 glucan synthase OAD00805.1
Lichtheimia corymbifera JMRC FSU 9682 glucan synthase CDH56336.1
Mucor circinelloides f. lusitanicus CBS 277.49 beta 1 3 glucan synthase OAD08543.1
Lichtheimia corymbifera JMRC FSU 9682 beta glucan synthase component fks1 CDH49323.1
Rhizopus delemar RA 99 880 RO3G 10349 EIE85639.1
Mucor circinelloides f. lusitanicus CBS 277.49 beta 1 3 glucan synthase OAD02999.1
Pneumocystis jirovecii RU7 T551 02309 KTW29035.1
Candida albicans SC5314 1 3 beta glucan synthase AOW25926.1
Candida tropicalis MYA 3404 1 3 beta glucan synthase component GLS1 EER31878.1
Scedosporium apiospermum 1 3 beta glucan synthase component FKS1 KEZ45482.1
Sporothrix schenckii 1099 18 1 3 beta glucan synthase KJR87404.1
Purpureocillium lilacinum 1 3 beta glucan synthase component GLS2 OAQ93168.1
Exophiala oligosperma 1 3 beta glucan synthase component FKS1 KIW43650.1
Cladophialophora bantiana CBS 173.52 1 3 beta glucan synthase component FKS1 KIW89004.1
Rhinocladiella mackenziei CBS 650.93 1 3 beta glucan synthase component FKS1 KIX06895.1
Talaromyces marneffei ATCC 18224 1 3 beta glucan synthase catalytic subunit FksP EEA23984.1
Aspergillus fumigatus Af293 1 3 beta glucan synthase catalytic subunit FksP EAL89080.1
Aspergillus lentulus 1 3 beta glucan synthase component FKS1 GAQ02924.1
Verruconis gallopava 1 3 beta glucan synthase component FKS1 KIW02769.1
Paracoccidioides brasiliensis Pb18 1 3 beta glucan synthase component FKS1 KGM92054.1
Histoplasma capsulatum NAm1 1 3 beta glucan synthase component GLS1 EDN08790.1
Blastomyces dermatitidis ER 3 1 3 beta glucan synthase component FKS1 EEQ85063.1
Coccidioides immitis RS 1 3 beta glucan synthase component FKS1 EAS36399.3
Trichophyton rubrum CBS 118892 1 3 beta glucan synthase component FKS1 KFL60511.1
Trichophyton rubrum CBS 118892 1 3 beta glucan synthase component FKS1 EGD84854.1
Trichophyton rubrum CBS 118892 1 3 beta glucan synthase component FKS1 KFL60510.1
FKS1_dom1
Glucan_synthase
