# Supplementary material for: Diversity of Cell Wall Related Proteins in Human Pathogenic Fungi
Source: J Fungi (Basel). 2017 Dec 29;4(1):6. doi: 10.3390/jof4010006 (PMC5872309; doi:10.3390/jof4010006)
Supplement: Supplementary file 1 [file jof-04-00006-s001.zip › jof-252817-final-supplementary materials/Dataset S3_trees_pdf/GT49.pdf]

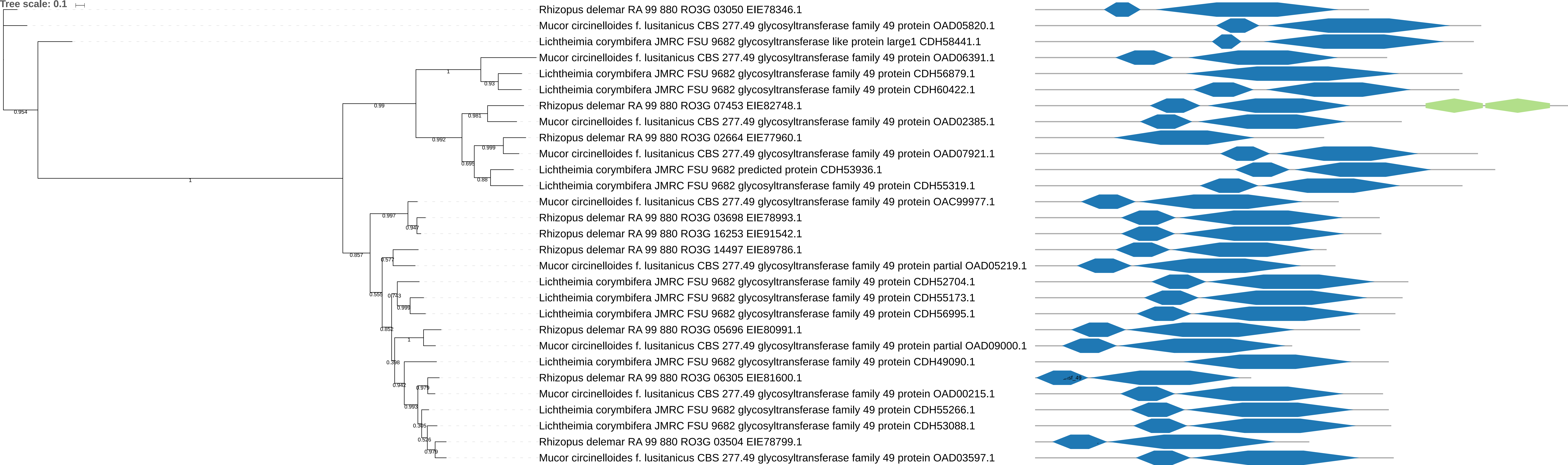
Tree scale: 0.1
Rhizopus delemar RA 99 880 RO3G 03050 EIE78346.1
Mucor circinelloides f. lusitanicus CBS 277.49 glycosyltransferase family 49 protein OAD05820.1
Lichtheimia corymbifera JMRC FSU 9682 glycosyltransferase like protein large1 CDH58441.1
Mucor circinelloides f. lusitanicus CBS 277.49 glycosyltransferase family 49 protein OAD06391.1
Lichtheimia corymbifera JMRC FSU 9682 glycosyltransferase family 49 protein CDH56879.1
Lichtheimia corymbifera JMRC FSU 9682 glycosyltransferase family 49 protein CDH60422.1
Rhizopus delemar RA 99 880 RO3G 07453 EIE82748.1
Mucor circinelloides f. lusitanicus CBS 277.49 glycosyltransferase family 49 protein OAD02385.1
Rhizopus delemar RA 99 880 RO3G 02664 EIE77960.1
Mucor circinelloides f. lusitanicus CBS 277.49 glycosyltransferase family 49 protein OAD07921.1
Lichtheimia corymbifera JMRC FSU 9682 predicted protein CDH53936.1
Lichtheimia corymbifera JMRC FSU 9682 glycosyltransferase family 49 protein CDH55319.1
Mucor circinelloides f. lusitanicus CBS 277.49 glycosyltransferase family 49 protein OAC99977.1
Rhizopus delemar RA 99 880 RO3G 03698 EIE78993.1
Rhizopus delemar RA 99 880 RO3G 16253 EIE91542.1
Rhizopus delemar RA 99 880 RO3G 14497 EIE89786.1
Mucor circinelloides f. lusitanicus CBS 277.49 glycosyltransferase family 49 protein partial OAD05219.1
Lichtheimia corymbifera JMRC FSU 9682 glycosyltransferase family 49 protein CDH52704.1
Lichtheimia corymbifera JMRC FSU 9682 glycosyltransferase family 49 protein CDH55173.1
Lichtheimia corymbifera JMRC FSU 9682 glycosyltransferase family 49 protein CDH56995.1
Rhizopus delemar RA 99 880 RO3G 05696 EIE80991.1
Mucor circinelloides f. lusitanicus CBS 277.49 glycosyltransferase family 49 protein partial OAD09000.1
Lichtheimia corymbifera JMRC FSU 9682 glycosyltransferase family 49 protein CDH49090.1
Rhizopus delemar RA 99 880 RO3G 06305 EIE81600.1
Mucor circinelloides f. lusitanicus CBS 277.49 glycosyltransferase family 49 protein OAD00215.1
Lichtheimia corymbifera JMRC FSU 9682 glycosyltransferase family 49 protein CDH55266.1
Lichtheimia corymbifera JMRC FSU 9682 glycosyltransferase family 49 protein CDH53088.1
Rhizopus delemar RA 99 880 RO3G 03504 EIE78799.1
Mucor circinelloides f. lusitanicus CBS 277.49 glycosyltransferase family 49 protein OAD03597.1
