# Supplementary material for: Diversity of Cell Wall Related Proteins in Human Pathogenic Fungi
Source: J Fungi (Basel). 2017 Dec 29;4(1):6. doi: 10.3390/jof4010006 (PMC5872309; doi:10.3390/jof4010006)
Supplement: Supplementary file 1 [file jof-04-00006-s001.zip › jof-252817-final-supplementary materials/Dataset S3_trees_pdf/GT5.pdf]

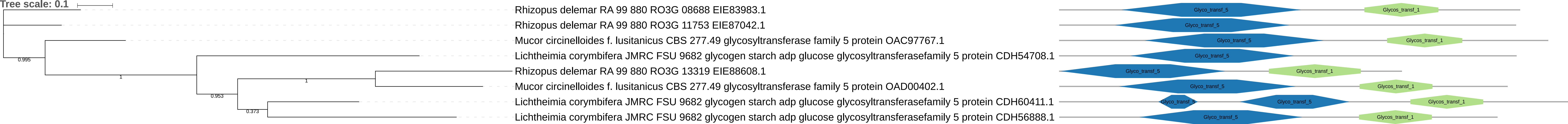
Tree scale: 0.1
Rhizopus delemar RA 99 880 RO3G 08688 EIE83983.1
Rhizopus delemar RA 99 880 RO3G 11753 EIE87042.1
Mucor circinelloides f. lusitanicus CBS 277.49 glycosyltransferase family 5 protein OAC97767.1
Lichtheimia corymbifera JMRC FSU 9682 glycogen starch adp glucose glycosyltransferasefamily 5 protein CDH54708.1
Rhizopus delemar RA 99 880 RO3G 13319 EIE88608.1
Mucor circinelloides f. lusitanicus CBS 277.49 glycosyltransferase family 5 protein OAD00402.1
Lichtheimia corymbifera JMRC FSU 9682 glycogen starch adp glucose glycosyltransferasefamily 5 protein CDH60411.1
Lichtheimia corymbifera JMRC FSU 9682 glycogen starch adp glucose glycosyltransferasefamily 5 protein CDH56888.1
0.995
1
1
0.953
0.373
Glyco_transf_5
Glycos_transf_1
