# Supplementary material for: Diversity of Cell Wall Related Proteins in Human Pathogenic Fungi
Source: J Fungi (Basel). 2017 Dec 29;4(1):6. doi: 10.3390/jof4010006 (PMC5872309; doi:10.3390/jof4010006)
Supplement: Supplementary file 1 [file jof-04-00006-s001.zip › jof-252817-final-supplementary materials/Dataset S3_trees_pdf/GT50.pdf]

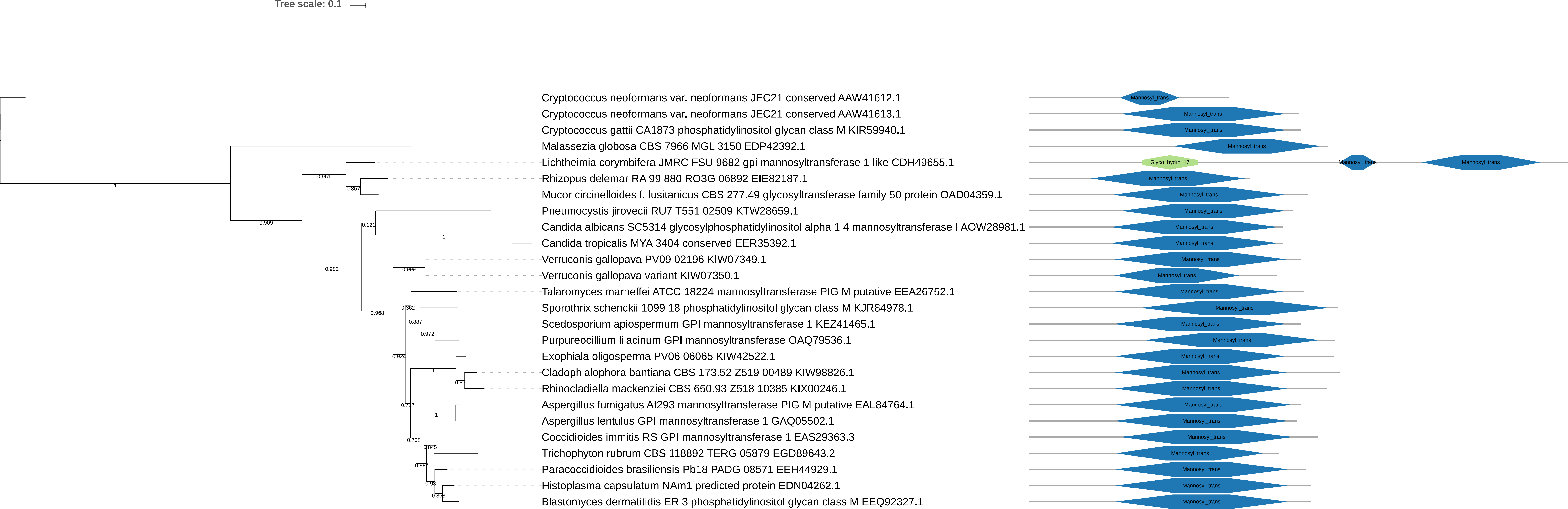
Tree scale: 0.1
Cryptococcus neoformans var. neoformans JEC21 conserved AAW41612.1
Cryptococcus neoformans var. neoformans JEC21 conserved AAW41613.1
Cryptococcus gattii CA1873 phosphatidylinositol glycan class M KIR59940.1
Malassezia globosa CBS 7966 MGL 3150 EDP42392.1
Lichtheimia corymbifera JMRC FSU 9682 gpi mannosyltransferase 1 like CDH49655.1
Rhizopus delemar RA 99 880 RO3G 06892 EIE82187.1
Mucor circinelloides f. lusitanicus CBS 277.49 glycosyltransferase family 50 protein OAD04359.1
Pneumocystis jirovecii RU7 T551 02509 KTW28659.1
Candida albicans SC5314 glycosylphosphatidylinositol alpha 1 4 mannosyltransferase I AOW28981.1
Candida tropicalis MYA 3404 conserved EER35392.1
Verruconis gallopava PV09 02196 KIW07349.1
Verruconis gallopava variant KIW07350.1
Talaromyces marneffei ATCC 18224 mannosyltransferase PIG M putative EEA26752.1
Sporothrix schenckii 1099 18 phosphatidylinositol glycan class M KJR84978.1
Scedosporium apiospermum GPI mannosyltransferase 1 KEZ41465.1
Purpureocillium lilacinum GPI mannosyltransferase OAQ79536.1
Exophiala oligosperma PV06 06065 KIW42522.1
Cladophialophora bantiana CBS 173.52 Z519 00489 KIW98826.1
Rhinocladiella mackenziei CBS 650.93 Z518 10385 KIX00246.1
Aspergillus fumigatus Af293 mannosyltransferase PIG M putative EAL84764.1
Aspergillus lentulus GPI mannosyltransferase 1 GAQ05502.1
Coccidioides immitis RS GPI mannosyltransferase 1 EAS29363.3
Trichophyton rubrum CBS 118892 TERG 05879 EGD89643.2
Paracoccidioides brasiliensis Pb18 PADG 08571 EEH44929.1
Histoplasma capsulatum NAm1 predicted protein EDN04262.1
Blastomyces dermatitidis ER 3 phosphatidylinositol glycan class M EEQ92327.1
Mannosyl_trans
Glyco_hydro_17
