# Supplementary material for: Diversity of Cell Wall Related Proteins in Human Pathogenic Fungi
Source: J Fungi (Basel). 2017 Dec 29;4(1):6. doi: 10.3390/jof4010006 (PMC5872309; doi:10.3390/jof4010006)
Supplement: Supplementary file 1 [file jof-04-00006-s001.zip › jof-252817-final-supplementary materials/Dataset S3_trees_pdf/GT57.pdf]

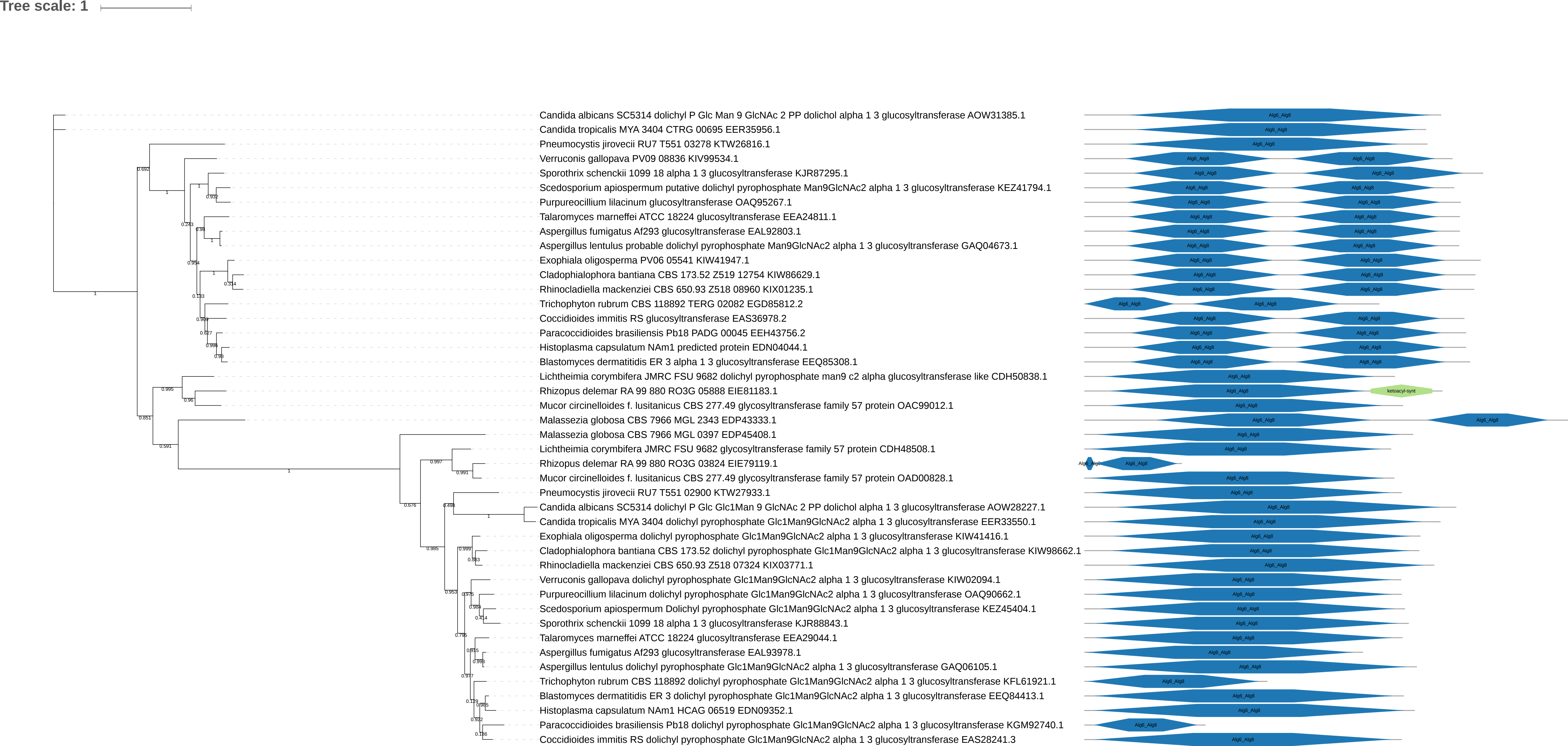

Tree scale: 1
Candida albicans SC5314 dolichyl P Glc Man 9 GlcNAc 2 PP dolichol alpha 1 3 glucosyltransferase AOW31385.1
Candida tropicalis MYA 3404 CTRG 00695 EER35956.1
Pneumocystis jirovecii RU7 T551 03278 KTW26816.1
Verruconis gallopava PV09 08836 KIV99534.1
Sporothrix schenckii 1099 18 alpha 1 3 glucosyltransferase KJR87295.1
Scedosporium apiospermum putative dolichyl pyrophosphate Man9GlcNAc2 alpha 1 3 glucosyltransferase KEZ41794.1
Purpureocillium lilacinum glucosyltransferase OAQ95267.1
Talaromyces marneffei ATCC 18224 glucosyltransferase EEA24811.1
Aspergillus fumigatus Af293 glucosyltransferase EAL92803.1
Aspergillus lentulus probable dolichyl pyrophosphate Man9GlcNAc2 alpha 1 3 glucosyltransferase GAQ04673.1
Exophiala oligosperma PV06 05541 KIW41947.1
Cladophialophora bantiana CBS 173.52 Z519 12754 KIW86629.1
Rhinocladiella mackenziei CBS 650.93 Z518 08960 KIX01235.1
Trichophyton rubrum CBS 118892 TERG 02082 EGD85812.2
Coccidioides immitis RS glucosyltransferase EAS36978.2
Paracoccidioides brasiliensis Pb18 PADG 00045 EEH43756.2
Histoplasma capsulatum NAm1 predicted protein EDN04044.1
Blastomyces dermatitidis ER 3 alpha 1 3 glucosyltransferase EEQ85308.1
Lichtheimia corymbifera JMRC FSU 9682 dolichyl pyrophosphate man9 c2 alpha glucosyltransferase like CDH50838.1
Rhizopus delemar RA 99 880 RO3G 05888 EIE81183.1
Mucor circinelloides f. lusitanicus CBS 277.49 glycosyltransferase family 57 protein OAC99012.1
Malassezia globosa CBS 7966 MGL 2343 EDP43333.1
Malassezia globosa CBS 7966 MGL 0397 EDP45408.1
Lichtheimia corymbifera JMRC FSU 9682 glycosyltransferase family 57 protein CDH48508.1
Rhizopus delemar RA 99 880 RO3G 03824 EIE79119.1
Mucor circinelloides f. lusitanicus CBS 277.49 glycosyltransferase family 57 protein OAD00828.1
Pneumocystis jirovecii RU7 T551 02900 KTW27933.1
Candida albicans SC5314 dolichyl P Glc Glc1Man 9 GlcNAc 2 PP dolichol alpha 1 3 glucosyltransferase AOW28227.1
Candida tropicalis MYA 3404 dolichyl pyrophosphate Glc1Man9GlcNAc2 alpha 1 3 glucosyltransferase EER33550.1
Exophiala oligosperma dolichyl pyrophosphate Glc1Man9GlcNAc2 alpha 1 3 glucosyltransferase KIW41416.1
Cladophialophora bantiana CBS 173.52 dolichyl pyrophosphate Glc1Man9GlcNAc2 alpha 1 3 glucosyltransferase KIW98662.1
Rhinocladiella mackenziei CBS 650.93 Z518 07324 KIX03771.1
Verruconis gallopava dolichyl pyrophosphate Glc1Man9GlcNAc2 alpha 1 3 glucosyltransferase KIW02094.1
Purpureocillium lilacinum dolichyl pyrophosphate Glc1Man9GlcNAc2 alpha 1 3 glucosyltransferase OAQ90662.1
Scedosporium apiospermum Dolichyl pyrophosphate Glc1Man9GlcNAc2 alpha 1 3 glucosyltransferase KEZ45404.1
Sporothrix schenckii 1099 18 alpha 1 3 glucosyltransferase KJR88843.1
Talaromyces marneffei ATCC 18224 glucosyltransferase EEA29044.1
Aspergillus fumigatus Af293 glucosyltransferase EAL93978.1
Aspergillus lentulus dolichyl pyrophosphate Glc1Man9GlcNAc2 alpha 1 3 glucosyltransferase GAQ06105.1
Trichophyton rubrum CBS 118892 dolichyl pyrophosphate Glc1Man9GlcNAc2 alpha 1 3 glucosyltransferase KFL61921.1
Blastomyces dermatitidis ER 3 dolichyl pyrophosphate Glc1Man9GlcNAc2 alpha 1 3 glucosyltransferase EEQ84413.1
Histoplasma capsulatum NAm1 HCAG 06519 EDN09352.1
Paracoccidioides brasiliensis Pb18 dolichyl pyrophosphate Glc1Man9GlcNAc2 alpha 1 3 glucosyltransferase KGM92740.1
Coccidioides immitis RS dolichyl pyrophosphate Glc1Man9GlcNAc2 alpha 1 3 glucosyltransferase EAS28241.3
Alg6_Alg8
ketoacyl-synt
