# Supplementary material for: Diversity of Cell Wall Related Proteins in Human Pathogenic Fungi
Source: J Fungi (Basel). 2017 Dec 29;4(1):6. doi: 10.3390/jof4010006 (PMC5872309; doi:10.3390/jof4010006)
Supplement: Supplementary file 1 [file jof-04-00006-s001.zip › jof-252817-final-supplementary materials/Dataset S3_trees_pdf/GT58.pdf]

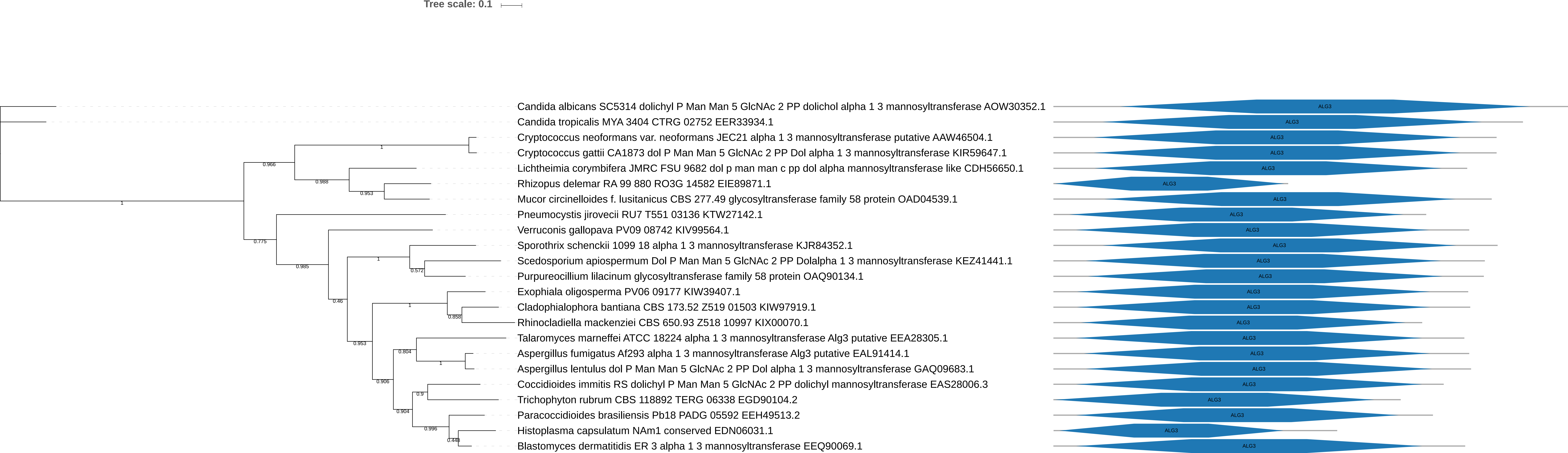
Tree scale: 0.1
Candida albicans SC5314 dolichyl P Man Man 5 GlcNAc 2 PP dolichol alpha 1 3 mannosyltransferase AOW30352.1
Candida tropicalis MYA 3404 CTRG 02752 EER33934.1
Cryptococcus neoformans var. neoformans JEC21 alpha 1 3 mannosyltransferase putative AAW46504.1
Cryptococcus gattii CA1873 dol P Man Man 5 GlcNAc 2 PP Dol alpha 1 3 mannosyltransferase KIR59647.1
Lichtheimia corymbifera JMRC FSU 9682 dol p man man c pp dol alpha mannosyltransferase like CDH56650.1
Rhizopus delemar RA 99 880 RO3G 14582 EIE89871.1
Mucor circinelloides f. lusitanicus CBS 277.49 glycosyltransferase family 58 protein OAD04539.1
Pneumocystis jirovecii RU7 T551 03136 KTW27142.1
Verruconis gallopava PV09 08742 KIV99564.1
Sporothrix schenckii 1099 18 alpha 1 3 mannosyltransferase KJR84352.1
Scedosporium apiospermum Dol P Man Man 5 GlcNAc 2 PP Dolalpha 1 3 mannosyltransferase KEZ41441.1
Purpureocillium lilacinum glycosyltransferase family 58 protein OAQ90134.1
Exophiala oligosperma PV06 09177 KIW39407.1
Cladophialophora bantiana CBS 173.52 Z519 01503 KIW97919.1
Rhinocladiella mackenziei CBS 650.93 Z518 10997 KIX00070.1
Talaromyces marneffei ATCC 18224 alpha 1 3 mannosyltransferase Alg3 putative EEA28305.1
Aspergillus fumigatus Af293 alpha 1 3 mannosyltransferase Alg3 putative EAL91414.1
Aspergillus lentulus dol P Man Man 5 GlcNAc 2 PP Dol alpha 1 3 mannosyltransferase GAQ09683.1
Coccidioides immitis RS dolichyl P Man Man 5 GlcNAc 2 PP dolichyl mannosyltransferase EAS28006.3
Trichophyton rubrum CBS 118892 TERG 06338 EGD90104.2
Paracoccidioides brasiliensis Pb18 PADG 05592 EEH49513.2
Histoplasma capsulatum NAm1 conserved EDN06031.1
Blastomyces dermatitidis ER 3 alpha 1 3 mannosyltransferase EEQ90069.1
ALG3
1
0.966
0.988
0.953
1
0.775
0.985
1
0.572
0.46
1
0.858
0.953
0.804
1
0.906
0.9
0.904
0.996
0.448
