# Supplementary material for: Diversity of Cell Wall Related Proteins in Human Pathogenic Fungi
Source: J Fungi (Basel). 2017 Dec 29;4(1):6. doi: 10.3390/jof4010006 (PMC5872309; doi:10.3390/jof4010006)
Supplement: Supplementary file 1 [file jof-04-00006-s001.zip › jof-252817-final-supplementary materials/Dataset S3_trees_pdf/GT59.pdf]

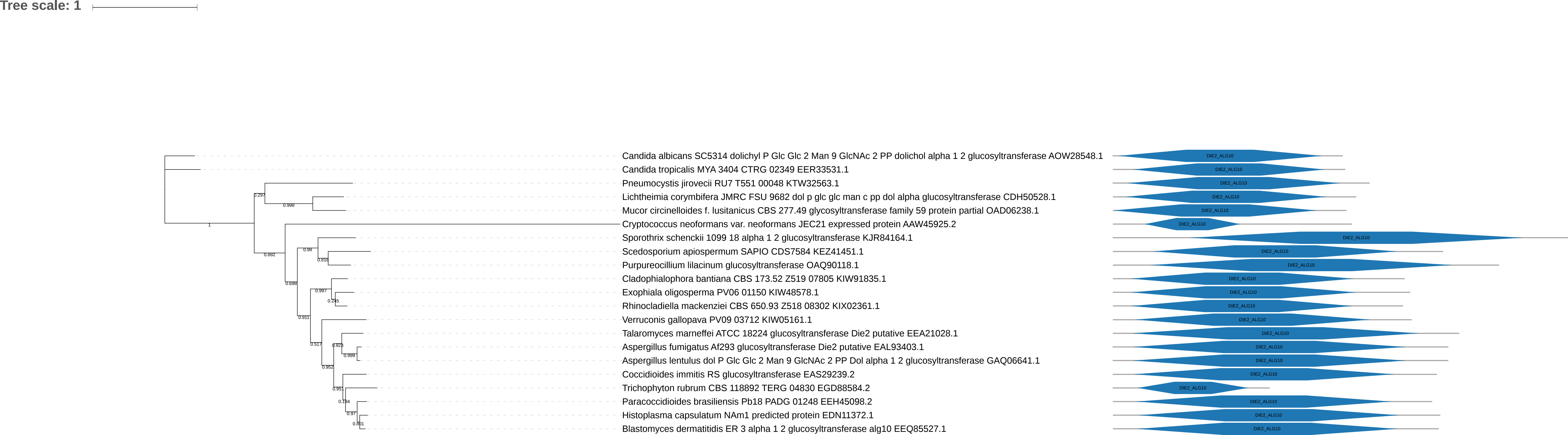
Tree scale: 1
Candida albicans SC5314 dolichyl P Glc Glc 2 Man 9 GlcNAc 2 PP dolichol alpha 1 2 glucosyltransferase AOW28548.1
Candida tropicalis MYA 3404 CTRG 02349 EER33531.1
Pneumocystis jirovecii RU7 T551 00048 KTW32563.1
Lichtheimia corymbifera JMRC FSU 9682 dol p glc glc man c pp dol alpha glucosyltransferase CDH50528.1
Mucor circinelloides f. lusitanicus CBS 277.49 glycosyltransferase family 59 protein partial OAD06238.1
Cryptococcus neoformans var. neoformans JEC21 expressed protein AAW45925.2
Sporothrix schenckii 1099 18 alpha 1 2 glucosyltransferase KJR84164.1
Scedosporium apiospermum SAPIO CDS7584 KEZ41451.1
Purpureocillium lilacinum glucosyltransferase OAQ90118.1
Cladophialophora bantiana CBS 173.52 Z519 07805 KIW91835.1
Exophiala oligosperma PV06 01150 KIW48578.1
Rhinocladiella mackenziei CBS 650.93 Z518 08302 KIX02361.1
Verruconis gallopava PV09 03712 KIW05161.1
Talaromyces marneffei ATCC 18224 glucosyltransferase Die2 putative EEA21028.1
Aspergillus fumigatus Af293 glucosyltransferase Die2 putative EAL93403.1
Aspergillus lentulus dol P Glc Glc 2 Man 9 GlcNAc 2 PP Dol alpha 1 2 glucosyltransferase GAQ06641.1
Coccidioides immitis RS glucosyltransferase EAS29239.2
Trichophyton rubrum CBS 118892 TERG 04830 EGD88584.2
Paracoccidioides brasiliensis Pb18 PADG 01248 EEH45098.2
Histoplasma capsulatum NAm1 predicted protein EDN11372.1
Blastomyces dermatitidis ER 3 alpha 1 2 glucosyltransferase alg10 EEQ85527.1
