# Supplementary material for: Diversity of Cell Wall Related Proteins in Human Pathogenic Fungi
Source: J Fungi (Basel). 2017 Dec 29;4(1):6. doi: 10.3390/jof4010006 (PMC5872309; doi:10.3390/jof4010006)
Supplement: Supplementary file 1 [file jof-04-00006-s001.zip › jof-252817-final-supplementary materials/Dataset S3_trees_pdf/GT62.pdf]

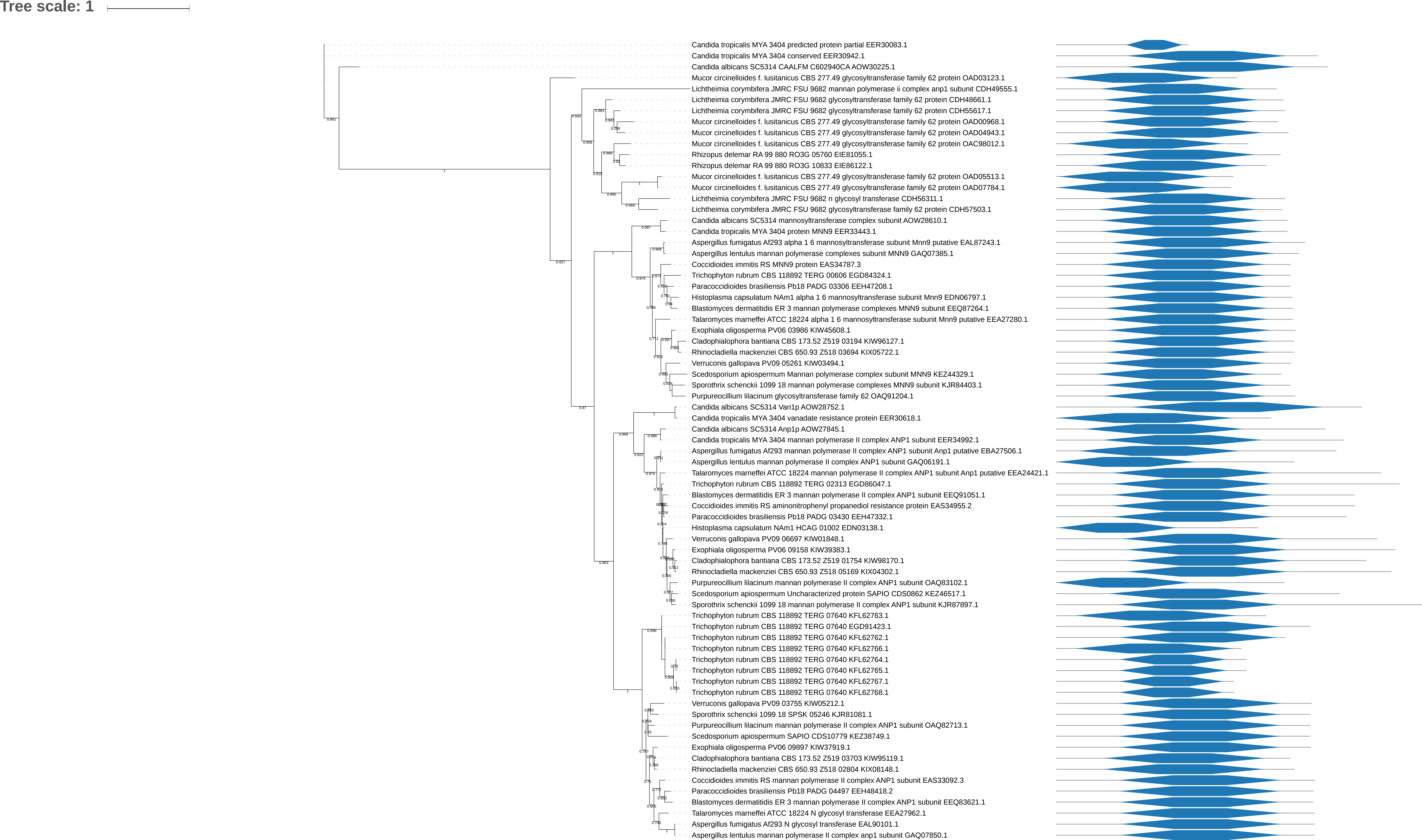

Tree scale: 1
Candida tropicalis MYA 3404 predicted protein partial EER30083.1
Candida tropicalis MYA 3404 conserved EER30942.1
Candida albicans SC5314 CAALFM C602940CA AOW30225.1
Mucor circinelloides f. lusitanicus CBS 277.49 glycosyltransferase family 62 protein OAD03123.1
Lichtheimia corymbifera JMRC FSU 9682 mannan polymerase ii complex anp1 subunit CDH49555.1
Lichtheimia corymbifera JMRC FSU 9682 glycosyltransferase family 62 protein CDH48661.1
Lichtheimia corymbifera JMRC FSU 9682 glycosyltransferase family 62 protein CDH55617.1
Mucor circinelloides f. lusitanicus CBS 277.49 glycosyltransferase family 62 protein OAD00968.1
Mucor circinelloides f. lusitanicus CBS 277.49 glycosyltransferase family 62 protein OAD04943.1
Mucor circinelloides f. lusitanicus CBS 277.49 glycosyltransferase family 62 protein OAC98012.1
Rhizopus delemar RA 99 880 RO3G 05760 EIE81055.1
Rhizopus delemar RA 99 880 RO3G 10833 EIE86122.1
Mucor circinelloides f. lusitanicus CBS 277.49 glycosyltransferase family 62 protein OAD05513.1
Mucor circinelloides f. lusitanicus CBS 277.49 glycosyltransferase family 62 protein OAD07784.1
Lichtheimia corymbifera JMRC FSU 9682 n glycosyl transferase CDH56311.1
Lichtheimia corymbifera JMRC FSU 9682 glycosyltransferase family 62 protein CDH57503.1
Candida albicans SC5314 mannosyltransferase complex subunit AOW28610.1
Candida tropicalis MYA 3404 protein MNN9 EER33443.1
Aspergillus fumigatus Af293 alpha 1 6 mannosyltransferase subunit Mnn9 putative EAL87243.1
Aspergillus lentulus mannan polymerase complexes subunit MNN9 GAQ07385.1
Coccidioides immitis RS MNN9 protein EAS34787.3
Trichophyton rubrum CBS 118892 TERG 00606 EGD84324.1
Paracoccidioides brasiliensis Pb18 PADG 03306 EEH47208.1
Histoplasma capsulatum NAm1 alpha 1 6 mannosyltransferase subunit Mnn9 EDN06797.1
Blastomyces dermatitidis ER 3 mannan polymerase complexes MNN9 subunit EEQ87264.1
Talaromyces marneffei ATCC 18224 alpha 1 6 mannosyltransferase subunit Mnn9 putative EEA27280.1
Exophiala oligosperma PV06 03986 KIW45608.1
Cladophialophora bantiana CBS 173.52 Z519 03194 KIW96127.1
Rhinocladiella mackenziei CBS 650.93 Z518 03694 KIX05722.1
Verruconis gallopava PV09 05261 KIW03494.1
Scedosporium apiospermum Mannan polymerase complex subunit MNN9 KEZ44329.1
Sporothrix schenckii 1099 18 mannan polymerase complexes MNN9 subunit KJR84403.1
Purpureocillium lilacinum glycosyltransferase family 62 OAQ91204.1
Candida albicans SC5314 Van1p AOW28752.1
Candida tropicalis MYA 3404 vanadate resistance protein EER30618.1
Candida albicans SC5314 Anp1p AOW27845.1
Candida tropicalis MYA 3404 mannan polymerase II complex ANP1 subunit EER34992.1
Aspergillus fumigatus Af293 mannan polymerase II complex ANP1 subunit Anp1 putative EBA27506.1
Aspergillus lentulus mannan polymerase II complex ANP1 subunit GAQ06191.1
Talaromyces marneffei ATCC 18224 mannan polymerase II complex ANP1 subunit Anp1 putative EEA24421.1
Trichophyton rubrum CBS 118892 TERG 02313 EGD86047.1
Blastomyces dermatitidis ER 3 mannan polymerase II complex ANP1 subunit EEQ91051.1
Coccidioides immitis RS aminonitrophenyl propanediol resistance protein EAS34955.2
Paracoccidioides brasiliensis Pb18 PADG 03430 EEH47332.1
Histoplasma capsulatum NAm1 HCAG 01002 EDN03138.1
Verruconis gallopava PV09 06697 KIW01848.1
Exophiala oligosperma PV06 09158 KIW39383.1
Cladophialophora bantiana CBS 173.52 Z519 01754 KIW98170.1
Rhinocladiella mackenziei CBS 650.93 Z518 05169 KIX04302.1
Purpureocillium lilacinum mannan polymerase II complex ANP1 subunit OAQ83102.1
Scedosporium apiospermum Uncharacterized protein SAPIO CDS0862 KEZ46517.1
Sporothrix schenckii 1099 18 mannan polymerase II complex ANP1 subunit KJR87897.1
Trichophyton rubrum CBS 118892 TERG 07640 KFL62763.1
Trichophyton rubrum CBS 118892 TERG 07640 EGD91423.1
Trichophyton rubrum CBS 118892 TERG 07640 KFL62762.1
Trichophyton rubrum CBS 118892 TERG 07640 KFL62766.1
Trichophyton rubrum CBS 118892 TERG 07640 KFL62764.1
Trichophyton rubrum CBS 118892 TERG 07640 KFL62765.1
Trichophyton rubrum CBS 118892 TERG 07640 KFL62767.1
Trichophyton rubrum CBS 118892 TERG 07640 KFL62768.1
Verruconis gallopava PV09 03755 KIW05212.1
Sporothrix schenckii 1099 18 SPSK 05246 KJR81081.1
Purpureocillium lilacinum mannan polymerase II complex ANP1 subunit OAQ82713.1
Scedosporium apiospermum SAPIO CDS10779 KEZ38749.1
Exophiala oligosperma PV06 09897 KIW37919.1
Cladophialophora bantiana CBS 173.52 Z519 03703 KIW95119.1
Rhinocladiella mackenziei CBS 650.93 Z518 02804 KIX08148.1
Coccidioides immitis RS mannan polymerase II complex ANP1 subunit EAS33092.3
Paracoccidioides brasiliensis Pb18 PADG 04497 EEH48418.2
Blastomyces dermatitidis ER 3 mannan polymerase II complex ANP1 subunit EEQ83621.1
Talaromyces marneffei ATCC 18224 N glycosyl transferase EEA27962.1
Aspergillus fumigatus Af293 N glycosyl transferase EAL90101.1
Aspergillus lentulus mannan polymerase II complex anp1 subunit GAQ07850.1
