# Supplementary material for: Diversity of Cell Wall Related Proteins in Human Pathogenic Fungi
Source: J Fungi (Basel). 2017 Dec 29;4(1):6. doi: 10.3390/jof4010006 (PMC5872309; doi:10.3390/jof4010006)
Supplement: Supplementary file 1 [file jof-04-00006-s001.zip › jof-252817-final-supplementary materials/Dataset S3_trees_pdf/GT66.pdf]

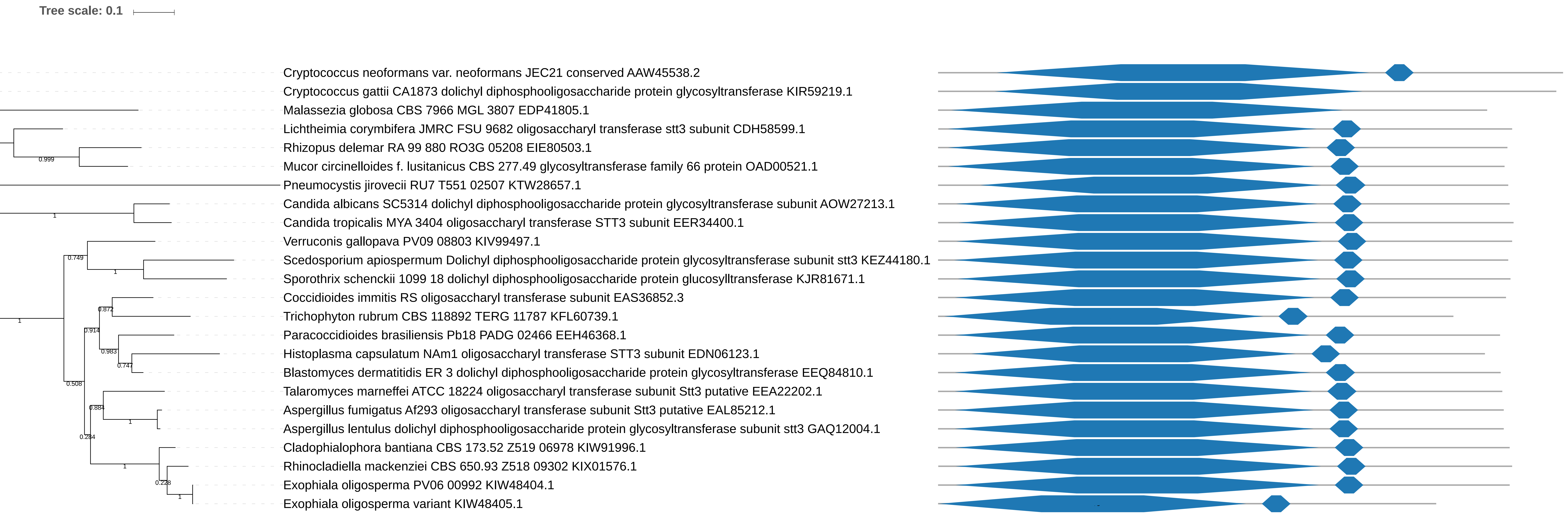
Tree scale: 0.1
Cryptococcus neoformans var. neoformans JEC21 conserved AAW45538.2
Cryptococcus gattii CA1873 dolichyl diphosphooligosaccharide protein glycosyltransferase KIR59219.1
Malassezia globosa CBS 7966 MGL 3807 EDP41805.1
Lichtheimia corymbifera JMRC FSU 9682 oligosaccharyl transferase stt3 subunit CDH58599.1
Rhizopus delemar RA 99 880 RO3G 05208 EIE80503.1
Mucor circinelloides f. lusitanicus CBS 277.49 glycosyltransferase family 66 protein OAD00521.1
Pneumocystis jirovecii RU7 T551 02507 KTW28657.1
Candida albicans SC5314 dolichyl diphosphooligosaccharide protein glycosyltransferase subunit AOW27213.1
Candida tropicalis MYA 3404 oligosaccharyl transferase STT3 subunit EER34400.1
Verruconis gallopava PV09 08803 KIV99497.1
Scedosporium apiospermum Dolichyl diphosphooligosaccharide protein glycosyltransferase subunit stt3 KEZ44180.1
Sporothrix schenckii 1099 18 dolichyl diphosphooligosaccharide protein glucosylltransferase KJR81671.1
Coccidioides immitis RS oligosaccharyl transferase subunit EAS36852.3
Trichophyton rubrum CBS 118892 TERG 11787 KFL60739.1
Paracoccidioides brasiliensis Pb18 PADG 02466 EEH46368.1
Histoplasma capsulatum NAm1 oligosaccharyl transferase STT3 subunit EDN06123.1
Blastomyces dermatitidis ER 3 dolichyl diphosphooligosaccharide protein glycosyltransferase EEQ84810.1
Talaromyces marneffei ATCC 18224 oligosaccharyl transferase subunit Stt3 putative EEA22202.1
Aspergillus fumigatus Af293 oligosaccharyl transferase subunit Stt3 putative EAL85212.1
Aspergillus lentulus dolichyl diphosphooligosaccharide protein glycosyltransferase subunit stt3 GAQ12004.1
Cladophialophora bantiana CBS 173.52 Z519 06978 KIW91996.1
Rhinocladiella mackenziei CBS 650.93 Z518 09302 KIX01576.1
Exophiala oligosperma PV06 00992 KIW48404.1
Exophiala oligosperma variant KIW48405.1
