# Supplementary material for: Diversity of Cell Wall Related Proteins in Human Pathogenic Fungi
Source: J Fungi (Basel). 2017 Dec 29;4(1):6. doi: 10.3390/jof4010006 (PMC5872309; doi:10.3390/jof4010006)
Supplement: Supplementary file 1 [file jof-04-00006-s001.zip › jof-252817-final-supplementary materials/Dataset S3_trees_pdf/GT69.pdf]

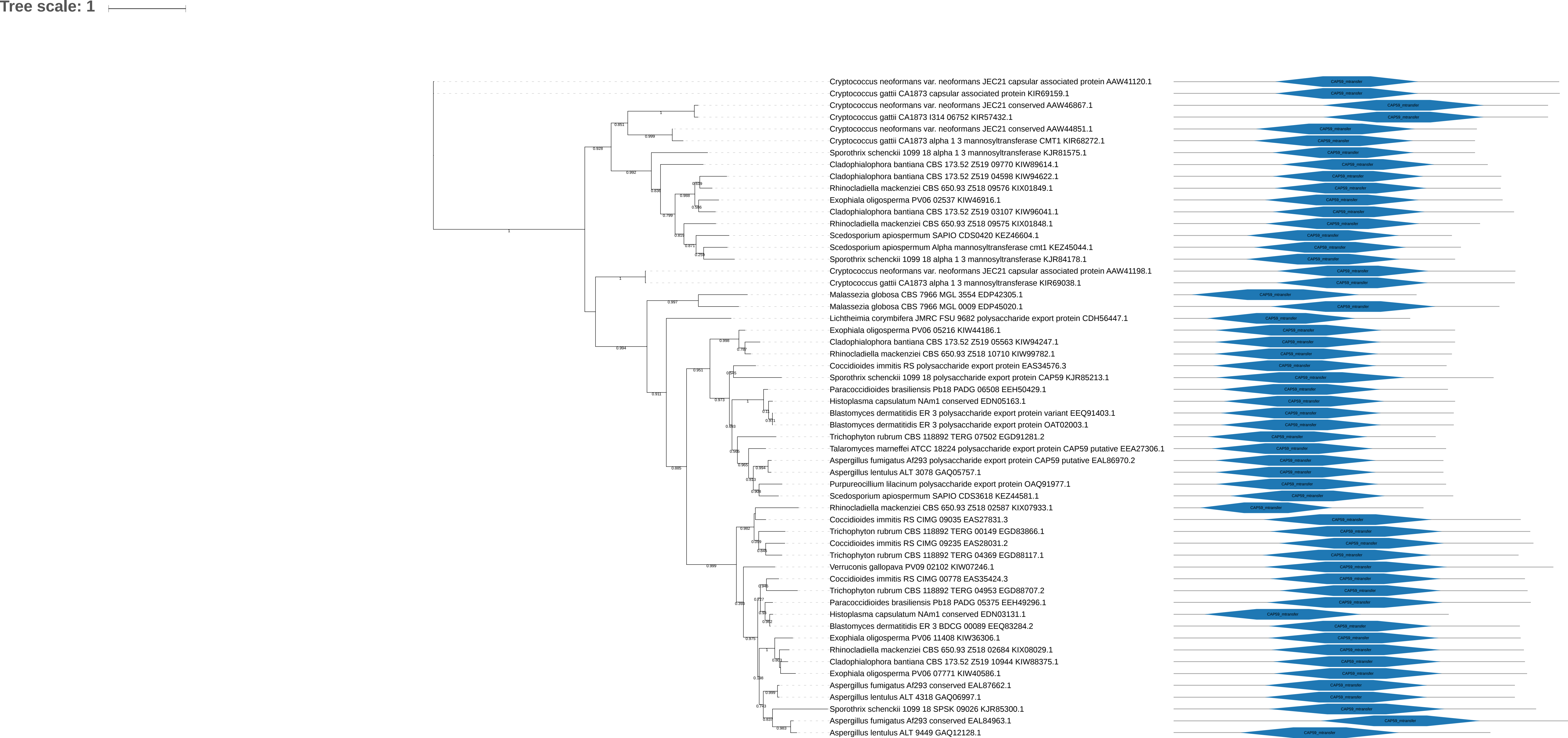
Tree scale: 1
Cryptococcus neoformans var. neoformans JEC21 capsular associated protein AAW41120.1
Cryptococcus gattii CA1873 capsular associated protein KIR69159.1
Cryptococcus neoformans var. neoformans JEC21 conserved AAW46867.1
Cryptococcus gattii CA1873 I314 06752 KIR57432.1
Cryptococcus neoformans var. neoformans JEC21 conserved AAW44851.1
Cryptococcus gattii CA1873 alpha 1 3 mannosyltransferase CMT1 KIR68272.1
Sporothrix schenckii 1099 18 alpha 1 3 mannosyltransferase KJR81575.1
Cladophialophora bantiana CBS 173.52 Z519 09770 KIW89614.1
Cladophialophora bantiana CBS 173.52 Z519 04598 KIW94622.1
Rhinocladiella mackenziei CBS 650.93 Z518 09576 KIX01849.1
Exophiala oligosperma PV06 02537 KIW46916.1
Cladophialophora bantiana CBS 173.52 Z519 03107 KIW96041.1
Rhinocladiella mackenziei CBS 650.93 Z518 09575 KIX01848.1
Scedosporium apiospermum SAPIO CDS0420 KEZ46604.1
Scedosporium apiospermum Alpha mannosyltransferase cmt1 KEZ45044.1
Sporothrix schenckii 1099 18 alpha 1 3 mannosyltransferase KJR84178.1
Cryptococcus neoformans var. neoformans JEC21 capsular associated protein AAW41198.1
Cryptococcus gattii CA1873 alpha 1 3 mannosyltransferase KIR69038.1
Malassezia globosa CBS 7966 MGL 3554 EDP42305.1
Malassezia globosa CBS 7966 MGL 0009 EDP45020.1
Lichtheimia corymbifera JMRC FSU 9682 polysaccharide export protein CDH56447.1
Exophiala oligosperma PV06 05216 KIW44186.1
Cladophialophora bantiana CBS 173.52 Z519 05563 KIW94247.1
Rhinocladiella mackenziei CBS 650.93 Z518 10710 KIW99782.1
Coccidioides immitis RS polysaccharide export protein EAS34576.3
Sporothrix schenckii 1099 18 polysaccharide export protein CAP59 KJR85213.1
Paracoccidioides brasiliensis Pb18 PADG 06508 EEH50429.1
Histoplasma capsulatum NAm1 conserved EDN05163.1
Blastomyces dermatitidis ER 3 polysaccharide export protein variant EEQ91403.1
Blastomyces dermatitidis ER 3 polysaccharide export protein OAT02003.1
Trichophyton rubrum CBS 118892 TERG 07502 EGD91281.2
Talaromyces marneffei ATCC 18224 polysaccharide export protein CAP59 putative EEA27306.1
Aspergillus fumigatus Af293 polysaccharide export protein CAP59 putative EAL86970.2
Aspergillus lentulus ALT 3078 GAQ05757.1
Purpureocillium lilacinum polysaccharide export protein OAQ91977.1
Scedosporium apiospermum SAPIO CDS3618 KEZ44581.1
Rhinocladiella mackenziei CBS 650.93 Z518 02587 KIX07933.1
Coccidioides immitis RS CIMG 09035 EAS27831.3
Trichophyton rubrum CBS 118892 TERG 00149 EGD83866.1
Coccidioides immitis RS CIMG 09235 EAS28031.2
Trichophyton rubrum CBS 118892 TERG 04369 EGD88117.1
Verruconis gallopava PV09 02102 KIW07246.1
Coccidioides immitis RS CIMG 00778 EAS35424.3
Trichophyton rubrum CBS 118892 TERG 04953 EGD88707.2
Paracoccidioides brasiliensis Pb18 PADG 05375 EEH49296.1
Histoplasma capsulatum NAm1 conserved EDN03131.1
Blastomyces dermatitidis ER 3 BDCG 00089 EEQ83284.2
Exophiala oligosperma PV06 11408 KIW36306.1
Rhinocladiella mackenziei CBS 650.93 Z518 02684 KIX08029.1
Cladophialophora bantiana CBS 173.52 Z519 10944 KIW88375.1
Exophiala oligosperma PV06 07771 KIW40586.1
Aspergillus fumigatus Af293 conserved EAL87662.1
Aspergillus lentulus ALT 4318 GAQ06997.1
Sporothrix schenckii 1099 18 SPSK 09026 KJR85300.1
Aspergillus fumigatus Af293 conserved EAL84963.1
Aspergillus lentulus ALT 9449 GAQ12128.1
