# Supplementary material for: Diversity of Cell Wall Related Proteins in Human Pathogenic Fungi
Source: J Fungi (Basel). 2017 Dec 29;4(1):6. doi: 10.3390/jof4010006 (PMC5872309; doi:10.3390/jof4010006)
Supplement: Supplementary file 1 [file jof-04-00006-s001.zip › jof-252817-final-supplementary materials/Dataset S3_trees_pdf/GT71.pdf]

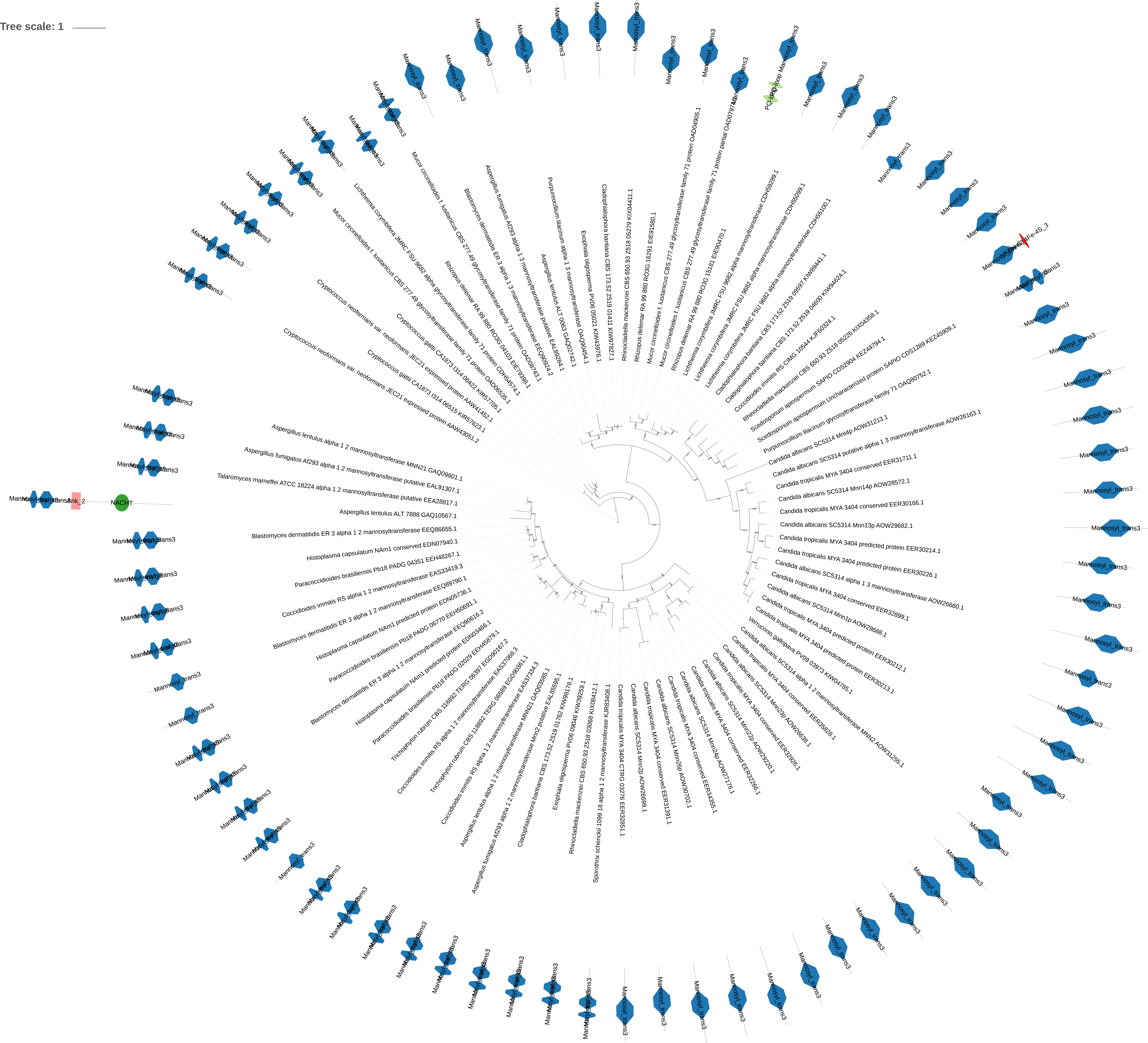
Tree scale: 1
Mucor circinelloides f. lusitanicus CBS 277.49 glycosyltransferase family 71 protein OAD08743.1
Blastomyces dermatitidis ER 3 alpha 1 3 mannosyltransferase EEQ90924.2
Aspergillus fumigatus Af293 alpha 1 3 mannosyltransferase putative EAL89284.1
Purpureocillium lilacinum alpha 1 3 mannosyltransferase OAQ90454.1
Aspergillus lentulus ALT 0063 GAQ02742.1
Exophiala oligosperma PV06 05021 KIW43976.1
Cladophialophora bantiana CBS 173.52 Z519 01411 KIW97877.1
Rhinocladiella mackenziei CBS 650.93 Z518 05279 KIX04411.1
Rhizopus delemar RA 99 880 RO3G 16291 EIE91580.1
Mucor circinelloides f. lusitanicus CBS 277.49 glycosyltransferase family 71 protein OAD04905.1
Mucor circinelloides f. lusitanicus CBS 277.49 glycosyltransferase family 71 protein partial OAD07974.1
Rhizopus delemar RA 99 880 RO3G 15181 EIE90470.1
Lichtheimia corymbifera JMRC FSU 9682 alpha mannosyltransferase CDH59289.1
Lichtheimia corymbifera JMRC FSU 9682 alpha mannosyltransferase CDH55099.1
Lichtheimia corymbifera JMRC FSU 9682 alpha mannosyltransferase CDH55100.1
Cladophialophora bantiana CBS 173.52 Z519 09597 KIW89441.1
Cladophialophora bantiana CBS 173.52 Z519 04600 KIW94624.1
Coccidioides immitis RS CIMG 10544 KJF60324.1
Rhinocladiella mackenziei CBS 650.93 Z518 05226 KIX04358.1
Scedosporium apiospermum SAPIO CDS2904 KEZ44794.1
Scedosporium apiospermum Uncharacterized protein SAPIO CDS1289 KEZ45909.1
Purpureocillium lilacinum glycosyltransferase family 71 OAQ80752.1
Candida albicans SC5314 Mnt4p AOW31213.1
Candida albicans SC5314 putative alpha 1 3 mannosyltransferase AOW26163.1
Candida tropicalis MYA 3404 conserved EER31711.1
Candida albicans SC5314 Mnn14p AOW28572.1
Candida tropicalis MYA 3404 conserved EER30166.1
Candida albicans SC5314 Mnn13p AOW29682.1
Candida tropicalis MYA 3404 predicted protein EER30214.1
Candida tropicalis MYA 3404 predicted protein EER30226.1
Candida albicans SC5314 alpha 1 3 mannosyltransferase AOW26660.1
Candida tropicalis MYA 3404 conserved EER32899.1
Candida albicans SC5314 Mnn1p AOW29688.1
Candida tropicalis MYA 3404 predicted protein EER30212.1
Candida tropicalis MYA 3404 predicted protein EER30213.1
Verruconis gallopava PV09 03973 KIW04785.1
Candida albicans SC5314 alpha 1 2 mannosyltransferase MNN2 AOW31255.1
Candida tropicalis MYA 3404 conserved EER35828.1
Candida albicans SC5314 Mnn23p AOW26638.1
Candida tropicalis MYA 3404 conserved EER32926.1
Candida albicans SC5314 Mnn22p AOW29220.1
Candida tropicalis MYA 3404 conserved EER32266.1
Candida albicans SC5314 Mnn24p AOW27176.1
Candida tropicalis MYA 3404 conserved EER34355.1
Candida albicans SC5314 Mnn26p AOW30702.1
Candida tropicalis MYA 3404 conserved EER31391.1
Candida albicans SC5314 Mnn2p AOW26699.1
Candida tropicalis MYA 3404 CTRG 03276 EER32851.1
Sporothrix schenckii 1099 18 alpha 1 2 mannosyltransferase KJR83408.1
Rhinocladiella mackenziei CBS 650.93 Z518 03068 KIX08412.1
Exophiala oligosperma PV06 09046 KIW39259.1
Cladophialophora bantiana CBS 173.52 Z519 01762 KIW98178.1
Aspergillus fumigatus Af293 alpha 1 2 mannosyltransferase Mnn2 putative EAL85595.1
Aspergillus lentulus alpha 1 2 mannosyltransferase MNN21 GAQ03585.1
Coccidioides immitis RS alpha 1 2 mannosyltransferase EAS37334.3
Trichophyton rubrum CBS 118892 TERG 06589 EGD90361.1
Coccidioides immitis RS alpha 1 2 mannosyltransferase EAS37068.3
Trichophyton rubrum CBS 118892 TERG 06397 EGD90167.2
Paracoccidioides brasiliensis Pb18 PADG 02029 EEH45879.1
Histoplasma capsulatum NAm1 predicted protein EDN03466.1
Blastomyces dermatitidis ER 3 alpha 1 2 mannosyltransferase EEQ90616.2
Paracoccidioides brasiliensis Pb18 PADG 06770 EEH50691.1
Histoplasma capsulatum NAm1 predicted protein EDN05736.1
Blastomyces dermatitidis ER 3 alpha 1 2 mannosyltransferase EEQ89790.1
Coccidioides immitis RS alpha 1 2 mannosyltransferase EAS33419.3
Paracoccidioides brasiliensis Pb18 PADG 04351 EEH48267.1
Histoplasma capsulatum NAm1 conserved EDN07940.1
Blastomyces dermatitidis ER 3 alpha 1 2 mannosyltransferase EEQ86655.1
Aspergillus lentulus ALT 7888 GAQ10567.1
Talaromyces marneffei ATCC 18224 alpha 1 2 mannosyltransferase putative EEA28817.1
Aspergillus fumigatus Af293 alpha 1 2 mannosyltransferase putative EAL91307.1
Aspergillus lentulus alpha 1 2 mannosyltransferase MNN21 GAQ09801.1
Cryptococcus neoformans var. neoformans JEC21 expressed protein AAW43051.2
Cryptococcus gattii CA1873 I314 06515 KIR57623.1
Cryptococcus neoformans var. neoformans JEC21 expressed protein AAW41452.1
Cryptococcus gattii CA1873 I314 06422 KIR57705.1
Mucor circinelloides f. lusitanicus CBS 277.49 glycosyltransferase family 71 protein OAD06535.1
Lichtheimia corymbifera JMRC FSU 9682 alpha glycosyltransferase family 71 protein CDH54574.1
Rhizopus delemar RA 99 880 RO3G 04103 EIE79398.1
Mannosyl_trans3
PQ-loop
Ank_2
NACHT
