# Supplementary material for: Diversity of Cell Wall Related Proteins in Human Pathogenic Fungi
Source: J Fungi (Basel). 2017 Dec 29;4(1):6. doi: 10.3390/jof4010006 (PMC5872309; doi:10.3390/jof4010006)
Supplement: Supplementary file 1 [file jof-04-00006-s001.zip › jof-252817-final-supplementary materials/Dataset S3_trees_pdf/GT76.pdf]

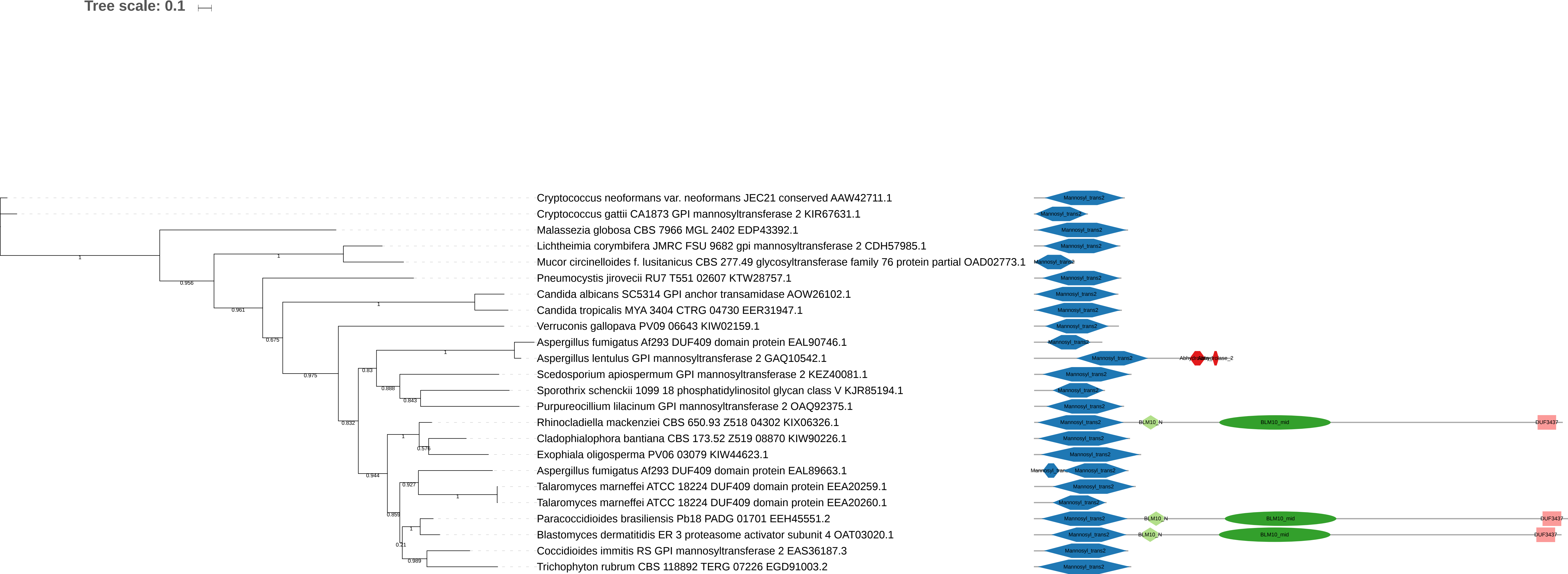

Tree scale: 0.1
Cryptococcus neoformans var. neoformans JEC21 conserved AAW42711.1
Cryptococcus gattii CA1873 GPI mannosyltransferase 2 KIR67631.1
Malassezia globosa CBS 7966 MGL 2402 EDP43392.1
Lichtheimia corymbifera JMRC FSU 9682 gpi mannosyltransferase 2 CDH57985.1
Mucor circinelloides f. lusitanicus CBS 277.49 glycosyltransferase family 76 protein partial OAD02773.1
Pneumocystis jirovecii RU7 T551 02607 KTW28757.1
Candida albicans SC5314 GPI anchor transamidase AOW26102.1
Candida tropicalis MYA 3404 CTRG 04730 EER31947.1
Verruconis gallopava PV09 06643 KIW02159.1
Aspergillus fumigatus Af293 DUF409 domain protein EAL90746.1
Aspergillus lentulus GPI mannosyltransferase 2 GAQ10542.1
Scedosporium apiospermum GPI mannosyltransferase 2 KEZ40081.1
Sporothrix schenckii 1099 18 phosphatidylinositol glycan class V KJR85194.1
Purpureocillium lilacinum GPI mannosyltransferase 2 OAQ92375.1
Rhinocladiella mackenziei CBS 650.93 Z518 04302 KIX06326.1
Cladophialophora bantiana CBS 173.52 Z519 08870 KIW90226.1
Exophiala oligosperma PV06 03079 KIW44623.1
Aspergillus fumigatus Af293 DUF409 domain protein EAL89663.1
Talaromyces marneffei ATCC 18224 DUF409 domain protein EEA20259.1
Talaromyces marneffei ATCC 18224 DUF409 domain protein EEA20260.1
Paracoccidioides brasiliensis Pb18 PADG 01701 EEH45551.2
Blastomyces dermatitidis ER 3 proteasome activator subunit 4 OAT03020.1
Coccidioides immitis RS GPI mannosyltransferase 2 EAS36187.3
Trichophyton rubrum CBS 118892 TERG 07226 EGD91003.2
1
0.956
1
0.961
0.675
1
0.975
1
0.83
0.888
0.843
0.832
1
0.576
0.944
0.927
1
0.859
1
0.21
0.989
Mannosyl_trans2
Abhydrolase_2
BLM10_N
BLM10_mid
DUF3437
