# Supplementary material for: Diversity of Cell Wall Related Proteins in Human Pathogenic Fungi
Source: J Fungi (Basel). 2017 Dec 29;4(1):6. doi: 10.3390/jof4010006 (PMC5872309; doi:10.3390/jof4010006)
Supplement: Supplementary file 1 [file jof-04-00006-s001.zip › jof-252817-final-supplementary materials/Dataset S3_trees_pdf/GT91.pdf]

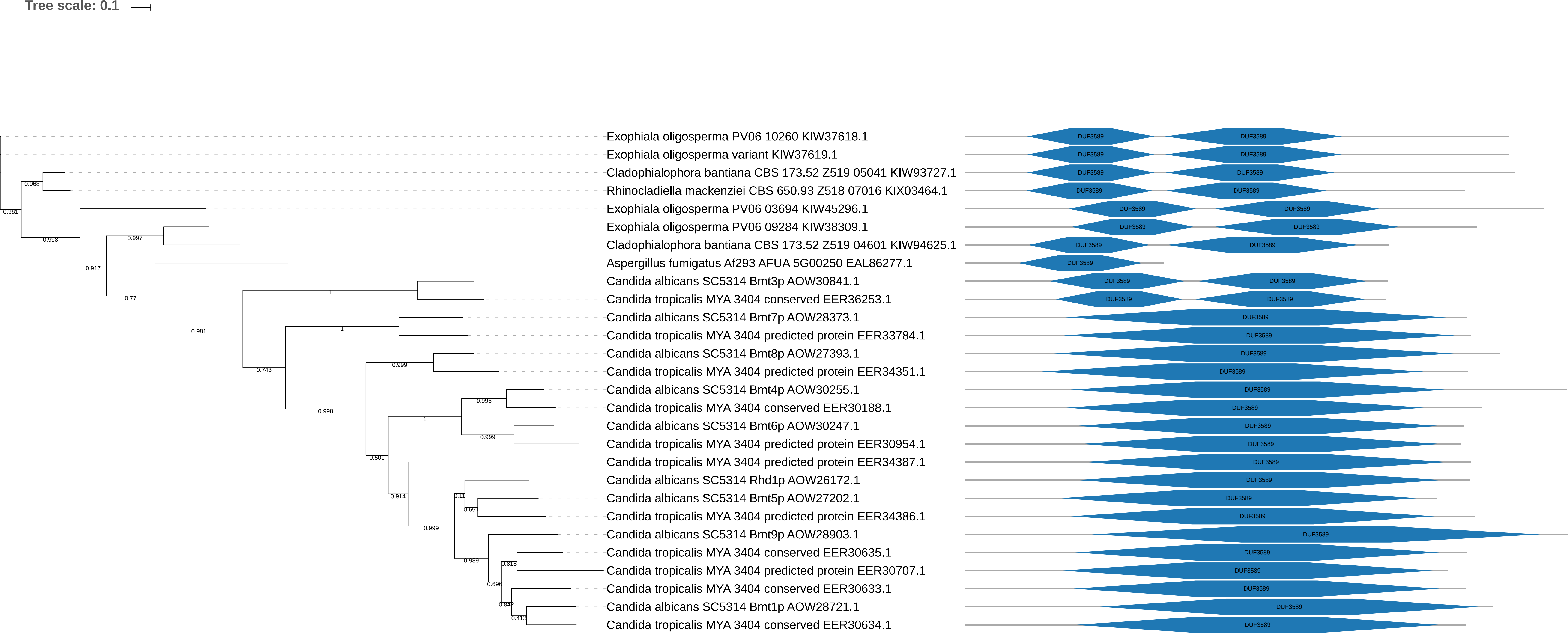
Tree scale: 0.1
Exophiala oligosperma PV06 10260 KIW37618.1
Exophiala oligosperma variant KIW37619.1
Cladophialophora bantiana CBS 173.52 Z519 05041 KIW93727.1
Rhinocladiella mackenziei CBS 650.93 Z518 07016 KIX03464.1
Exophiala oligosperma PV06 03694 KIW45296.1
Exophiala oligosperma PV06 09284 KIW38309.1
Cladophialophora bantiana CBS 173.52 Z519 04601 KIW94625.1
Aspergillus fumigatus Af293 AFUA 5G00250 EAL86277.1
Candida albicans SC5314 Bmt3p AOW30841.1
Candida tropicalis MYA 3404 conserved EER36253.1
Candida albicans SC5314 Bmt7p AOW28373.1
Candida tropicalis MYA 3404 predicted protein EER33784.1
Candida albicans SC5314 Bmt8p AOW27393.1
Candida tropicalis MYA 3404 predicted protein EER34351.1
Candida albicans SC5314 Bmt4p AOW30255.1
Candida tropicalis MYA 3404 conserved EER30188.1
Candida albicans SC5314 Bmt6p AOW30247.1
Candida tropicalis MYA 3404 predicted protein EER30954.1
Candida tropicalis MYA 3404 predicted protein EER34387.1
Candida albicans SC5314 Rhd1p AOW26172.1
Candida albicans SC5314 Bmt5p AOW27202.1
Candida tropicalis MYA 3404 predicted protein EER34386.1
Candida albicans SC5314 Bmt9p AOW28903.1
Candida tropicalis MYA 3404 conserved EER30635.1
Candida tropicalis MYA 3404 predicted protein EER30707.1
Candida tropicalis MYA 3404 conserved EER30633.1
Candida albicans SC5314 Bmt1p AOW28721.1
Candida tropicalis MYA 3404 conserved EER30634.1
DUF3589
