# Supplementary material for: Diversity of Cell Wall Related Proteins in Human Pathogenic Fungi
Source: J Fungi (Basel). 2017 Dec 29;4(1):6. doi: 10.3390/jof4010006 (PMC5872309; doi:10.3390/jof4010006)
Supplement: Supplementary file 1 [file jof-04-00006-s001.zip › jof-252817-final-supplementary materials/Dataset S3_trees_pdf/HsbA.pdf]

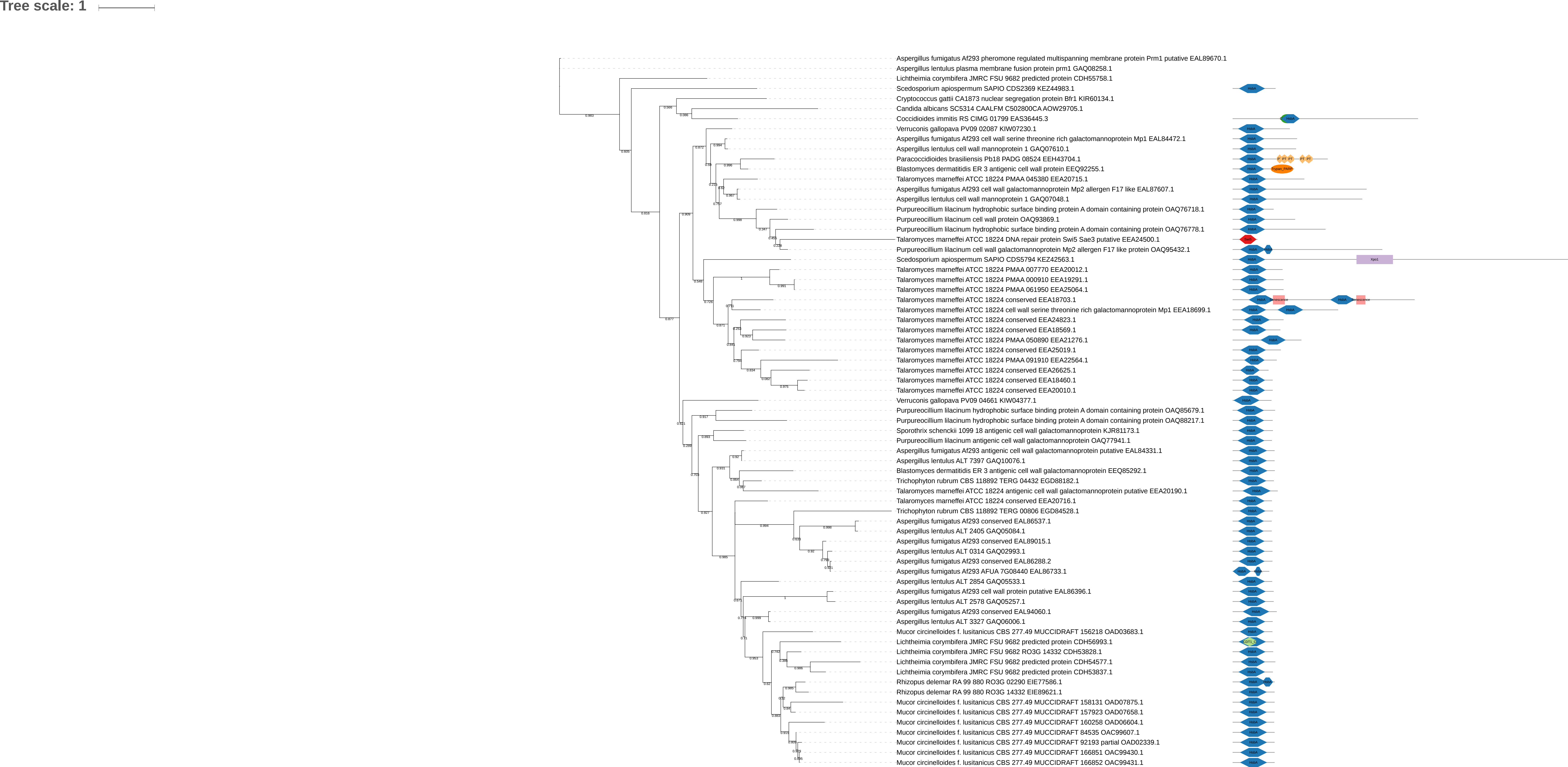
Tree scale: 1
Aspergillus fumigatus Af293 pheromone regulated multispanning membrane protein Prm1 putative EAL89670.1
Aspergillus lentulus plasma membrane fusion protein prm1 GAQ08258.1
Lichtheimia corymbifera JMRC FSU 9682 predicted protein CDH55758.1
Scedosporium apiospermum SAPIO CDS2369 KEZ44983.1
Cryptococcus gattii CA1873 nuclear segregation protein Bfr1 KIR60134.1
Candida albicans SC5314 CAALFM C502800CA AOW29705.1
Coccidioides immitis RS CIMG 01799 EAS36445.3
Verruconis gallopava PV09 02087 KIW07230.1
Aspergillus fumigatus Af293 cell wall serine threonine rich galactomannoprotein Mp1 EAL84472.1
Aspergillus lentulus cell wall mannoprotein 1 GAQ07610.1
Paracoccidioides brasiliensis Pb18 PADG 08524 EEH43704.1
Blastomyces dermatitidis ER 3 antigenic cell wall protein EEQ92255.1
Talaromyces marneffei ATCC 18224 PMAA 045380 EEA20715.1
Aspergillus fumigatus Af293 cell wall galactomannoprotein Mp2 allergen F17 like EAL87607.1
Aspergillus lentulus cell wall mannoprotein 1 GAQ07048.1
Purpureocillium lilacinum hydrophobic surface binding protein A domain containing protein OAQ76718.1
Purpureocillium lilacinum cell wall protein OAQ93869.1
Purpureocillium lilacinum hydrophobic surface binding protein A domain containing protein OAQ76778.1
Talaromyces marneffei ATCC 18224 DNA repair protein Swi5 Sae3 putative EEA24500.1
Purpureocillium lilacinum cell wall galactomannoprotein Mp2 allergen F17 like protein OAQ95432.1
Scedosporium apiospermum SAPIO CDS5794 KEZ42563.1
Talaromyces marneffei ATCC 18224 PMAA 007770 EEA20012.1
Talaromyces marneffei ATCC 18224 PMAA 000910 EEA19291.1
Talaromyces marneffei ATCC 18224 PMAA 061950 EEA25064.1
Talaromyces marneffei ATCC 18224 conserved EEA18703.1
Talaromyces marneffei ATCC 18224 cell wall serine threonine rich galactomannoprotein Mp1 EEA18699.1
Talaromyces marneffei ATCC 18224 conserved EEA24823.1
Talaromyces marneffei ATCC 18224 conserved EEA18569.1
Talaromyces marneffei ATCC 18224 PMAA 050890 EEA21276.1
Talaromyces marneffei ATCC 18224 conserved EEA25019.1
Talaromyces marneffei ATCC 18224 PMAA 091910 EEA22564.1
Talaromyces marneffei ATCC 18224 conserved EEA26625.1
Talaromyces marneffei ATCC 18224 conserved EEA18460.1
Talaromyces marneffei ATCC 18224 conserved EEA20010.1
Verruconis gallopava PV09 04661 KIW04377.1
Purpureocillium lilacinum hydrophobic surface binding protein A domain containing protein OAQ85679.1
Purpureocillium lilacinum hydrophobic surface binding protein A domain containing protein OAQ88217.1
Sporothrix schenckii 1099 18 antigenic cell wall galactomannoprotein KJR81173.1
Purpureocillium lilacinum antigenic cell wall galactomannoprotein OAQ77941.1
Aspergillus fumigatus Af293 antigenic cell wall galactomannoprotein putative EAL84331.1
Aspergillus lentulus ALT 7397 GAQ10076.1
Blastomyces dermatitidis ER 3 antigenic cell wall galactomannoprotein EEQ85292.1
Trichophyton rubrum CBS 118892 TERG 04432 EGD88182.1
Talaromyces marneffei ATCC 18224 antigenic cell wall galactomannoprotein putative EEA20190.1
Talaromyces marneffei ATCC 18224 conserved EEA20716.1
Trichophyton rubrum CBS 118892 TERG 00806 EGD84528.1
Aspergillus fumigatus Af293 conserved EAL86537.1
Aspergillus lentulus ALT 2405 GAQ05084.1
Aspergillus fumigatus Af293 conserved EAL89015.1
Aspergillus lentulus ALT 0314 GAQ02993.1
Aspergillus fumigatus Af293 conserved EAL86288.2
Aspergillus fumigatus Af293 AFUA 7G08440 EAL86733.1
Aspergillus lentulus ALT 2854 GAQ05533.1
Aspergillus fumigatus Af293 cell wall protein putative EAL86396.1
Aspergillus lentulus ALT 2578 GAQ05257.1
Aspergillus fumigatus Af293 conserved EAL94060.1
Aspergillus lentulus ALT 3327 GAQ06006.1
Mucor circinelloides f. lusitanicus CBS 277.49 MUCCIRDRAFT 156218 OAD03683.1
Lichtheimia corymbifera JMRC FSU 9682 predicted protein CDH56993.1
Lichtheimia corymbifera JMRC FSU 9682 RO3G 14332 CDH53828.1
Lichtheimia corymbifera JMRC FSU 9682 predicted protein CDH54577.1
Lichtheimia corymbifera JMRC FSU 9682 predicted protein CDH53837.1
Rhizopus delemar RA 99 880 RO3G 02290 EIE77586.1
Rhizopus delemar RA 99 880 RO3G 14332 EIE89621.1
Mucor circinelloides f. lusitanicus CBS 277.49 MUCCIRDRAFT 158131 OAD07875.1
Mucor circinelloides f. lusitanicus CBS 277.49 MUCCIRDRAFT 157923 OAD07658.1
Mucor circinelloides f. lusitanicus CBS 277.49 MUCCIRDRAFT 160258 OAD06604.1
Mucor circinelloides f. lusitanicus CBS 277.49 MUCCIRDRAFT 84535 OAC99607.1
Mucor circinelloides f. lusitanicus CBS 277.49 MUCCIRDRAFT 92193 partial OAD02339.1
Mucor circinelloides f. lusitanicus CBS 277.49 MUCCIRDRAFT 166851 OAC99430.1
Mucor circinelloides f. lusitanicus CBS 277.49 MUCCIRDRAFT 166852 OAC99431.1
