# Supplementary material for: Diversity of Cell Wall Related Proteins in Human Pathogenic Fungi
Source: J Fungi (Basel). 2017 Dec 29;4(1):6. doi: 10.3390/jof4010006 (PMC5872309; doi:10.3390/jof4010006)
Supplement: Supplementary file 1 [file jof-04-00006-s001.zip › jof-252817-final-supplementary materials/Dataset S3_trees_pdf/KRE9.pdf]

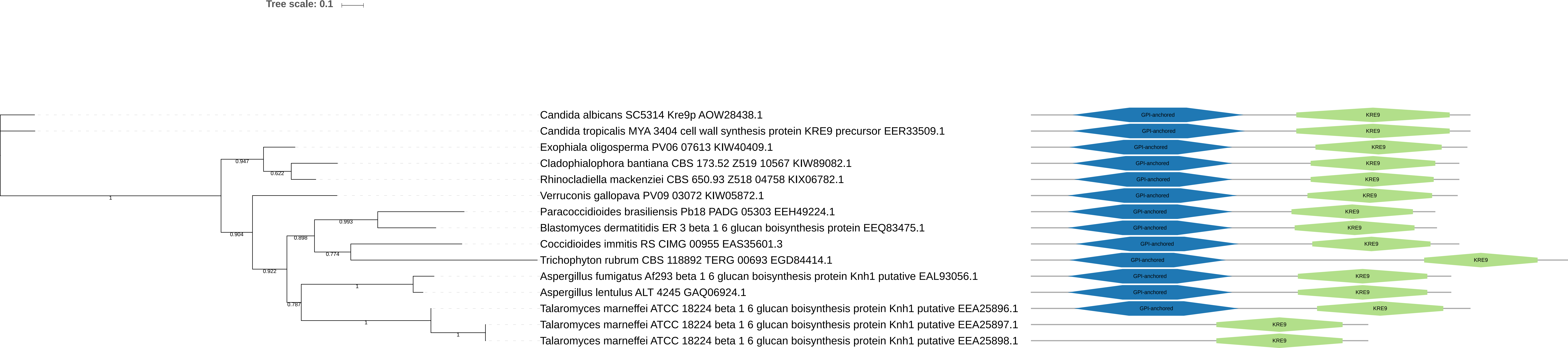

Tree scale: 0.1
Candida albicans SC5314 Kre9p AOW28438.1
Candida tropicalis MYA 3404 cell wall synthesis protein KRE9 precursor EER33509.1
Exophiala oligosperma PV06 07613 KIW40409.1
Cladophialophora bantiana CBS 173.52 Z519 10567 KIW89082.1
Rhinocladiella mackenziei CBS 650.93 Z518 04758 KIX06782.1
Verruconis gallopava PV09 03072 KIW05872.1
Paracoccidioides brasiliensis Pb18 PADG 05303 EEH49224.1
Blastomyces dermatitidis ER 3 beta 1 6 glucan boisynthesis protein EEQ83475.1
Coccidioides immitis RS CIMG 00955 EAS35601.3
Trichophyton rubrum CBS 118892 TERG 00693 EGD84414.1
Aspergillus fumigatus Af293 beta 1 6 glucan boisynthesis protein Knh1 putative EAL93056.1
Aspergillus lentulus ALT 4245 GAQ06924.1
Talaromyces marneffei ATCC 18224 beta 1 6 glucan boisynthesis protein Knh1 putative EEA25896.1
Talaromyces marneffei ATCC 18224 beta 1 6 glucan boisynthesis protein Knh1 putative EEA25897.1
Talaromyces marneffei ATCC 18224 beta 1 6 glucan boisynthesis protein Knh1 putative EEA25898.1
1
0.947
0.622
0.904
0.993
0.898
0.774
0.922
1
0.787
1
1
GPI-anchored
KRE9
