# Supplementary material for: Diversity of Cell Wall Related Proteins in Human Pathogenic Fungi
Source: J Fungi (Basel). 2017 Dec 29;4(1):6. doi: 10.3390/jof4010006 (PMC5872309; doi:10.3390/jof4010006)
Supplement: Supplementary file 1 [file jof-04-00006-s001.zip › jof-252817-final-supplementary materials/Dataset S3_trees_pdf/PIR.pdf]

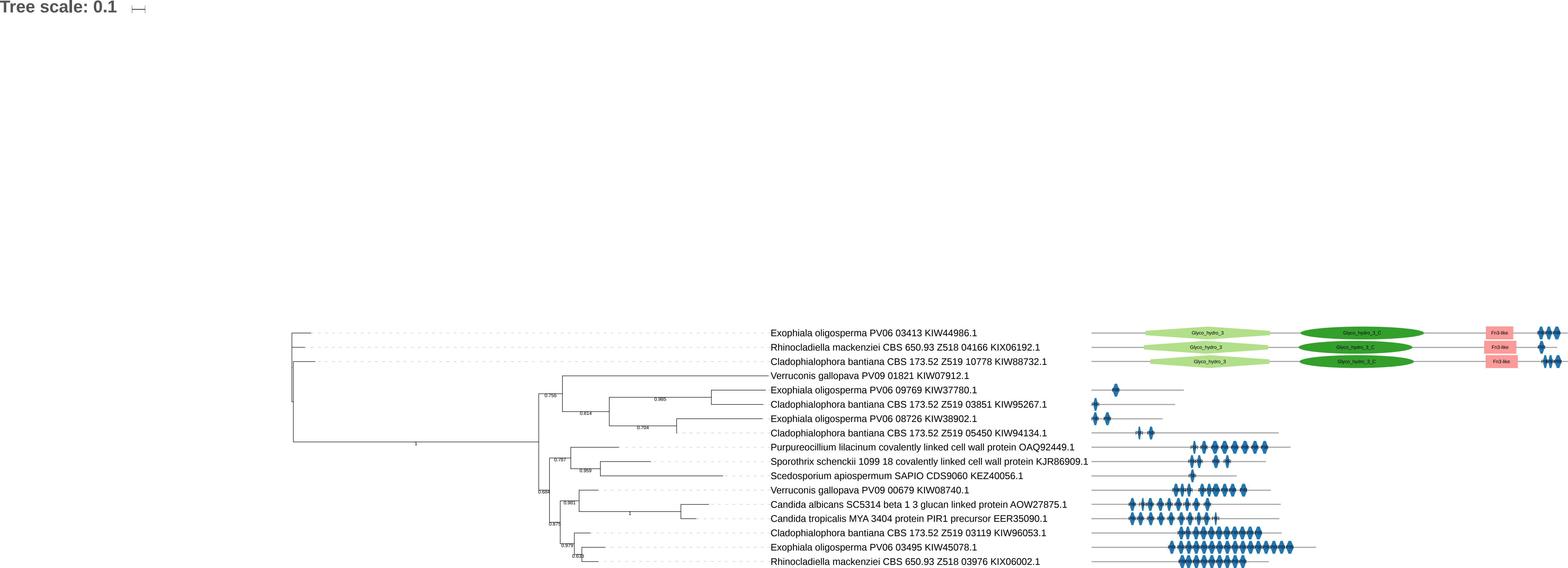
Tree scale: 0.1
Exophiala oligosperma PV06 03413 KIW44986.1
Rhinocladiella mackenziei CBS 650.93 Z518 04166 KIX06192.1
Cladophialophora bantiana CBS 173.52 Z519 10778 KIW88732.1
Verruconis gallopava PV09 01821 KIW07912.1
Exophiala oligosperma PV06 09769 KIW37780.1
Cladophialophora bantiana CBS 173.52 Z519 03851 KIW95267.1
Exophiala oligosperma PV06 08726 KIW38902.1
Cladophialophora bantiana CBS 173.52 Z519 05450 KIW94134.1
Purpureocillium lilacinum covalently linked cell wall protein OAQ92449.1
Sporothrix schenckii 1099 18 covalently linked cell wall protein KJR86909.1
Scedosporium apiospermum SAPIO CDS9060 KEZ40056.1
Verruconis gallopava PV09 00679 KIW08740.1
Candida albicans SC5314 beta 1 3 glucan linked protein AOW27875.1
Candida tropicalis MYA 3404 protein PIR1 precursor EER35090.1
Cladophialophora bantiana CBS 173.52 Z519 03119 KIW96053.1
Exophiala oligosperma PV06 03495 KIW45078.1
Rhinocladiella mackenziei CBS 650.93 Z518 03976 KIX06002.1
1
0.756
0.985
0.814
0.704
0.787
0.959
0.684
0.981
1
0.875
0.979
0.638
Glyco_hydro_3
Glyco_hydro_3_C
Fn3-like
PIR
