# Supplementary material for: Diversity of Cell Wall Related Proteins in Human Pathogenic Fungi
Source: J Fungi (Basel). 2017 Dec 29;4(1):6. doi: 10.3390/jof4010006 (PMC5872309; doi:10.3390/jof4010006)
Supplement: Supplementary file 1 [file jof-04-00006-s001.zip › jof-252817-final-supplementary materials/Dataset S3_trees_pdf/SodCu.pdf]

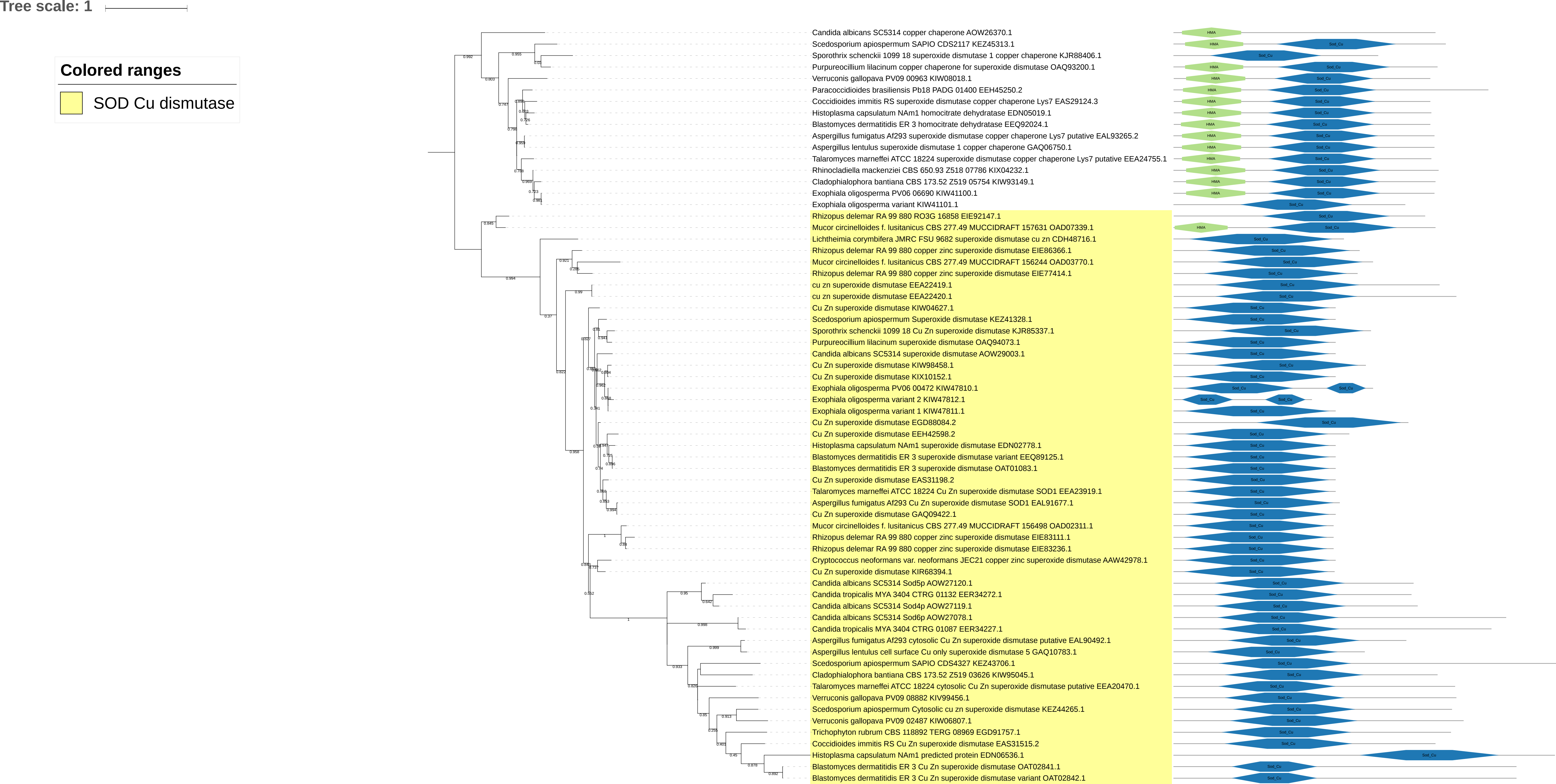
Tree scale: 1
Colored ranges
SOD Cu dismutase
Candida albicans SC5314 copper chaperone AOW26370.1
Scedosporium apiospermum SAPIO CDS2117 KEZ45313.1
Sporothrix schenckii 1099 18 superoxide dismutase 1 copper chaperone KJR88406.1
Purpureocillium lilacinum copper chaperone for superoxide dismutase OAQ93200.1
Verruconis gallopava PV09 00963 KIW08018.1
Paracoccidioides brasiliensis Pb18 PADG 01400 EEH45250.2
Coccidioides immitis RS superoxide dismutase copper chaperone Lys7 EAS29124.3
Histoplasma capsulatum NAm1 homocitrate dehydratase EDN05019.1
Blastomyces dermatitidis ER 3 homocitrate dehydratase EEQ92024.1
Aspergillus fumigatus Af293 superoxide dismutase copper chaperone Lys7 putative EAL93265.2
Aspergillus lentulus superoxide dismutase 1 copper chaperone GAQ06750.1
Talaromyces marneffei ATCC 18224 superoxide dismutase copper chaperone Lys7 putative EEA24755.1
Rhinocladiella mackenziei CBS 650.93 Z518 07786 KIX04232.1
Cladophialophora bantiana CBS 173.52 Z519 05754 KIW93149.1
Exophiala oligosperma PV06 06690 KIW41100.1
Exophiala oligosperma variant KIW41101.1
Rhizopus delemar RA 99 880 RO3G 16858 EIE92147.1
Mucor circinelloides f. lusitanicus CBS 277.49 MUCCIRDRAFT 157631 OAD07339.1
Lichtheimia corymbifera JMRC FSU 9682 superoxide dismutase cu zn CDH48716.1
Rhizopus delemar RA 99 880 copper zinc superoxide dismutase EIE86366.1
Mucor circinelloides f. lusitanicus CBS 277.49 MUCCIRDRAFT 156244 OAD03770.1
Rhizopus delemar RA 99 880 copper zinc superoxide dismutase EIE77414.1
cu zn superoxide dismutase EEA22419.1
cu zn superoxide dismutase EEA22420.1
Cu Zn superoxide dismutase KIW04627.1
Scedosporium apiospermum Superoxide dismutase KEZ41328.1
Sporothrix schenckii 1099 18 Cu Zn superoxide dismutase KJR85337.1
Purpureocillium lilacinum superoxide dismutase OAQ94073.1
Candida albicans SC5314 superoxide dismutase AOW29003.1
Cu Zn superoxide dismutase KIW98458.1
Cu Zn superoxide dismutase KIX10152.1
Exophiala oligosperma PV06 00472 KIW47810.1
Exophiala oligosperma variant 2 KIW47812.1
Exophiala oligosperma variant 1 KIW47811.1
Cu Zn superoxide dismutase EGD88084.2
Cu Zn superoxide dismutase EEH42598.2
Histoplasma capsulatum NAm1 superoxide dismutase EDN02778.1
Blastomyces dermatitidis ER 3 superoxide dismutase variant EEQ89125.1
Blastomyces dermatitidis ER 3 superoxide dismutase OAT01083.1
Cu Zn superoxide dismutase EAS31198.2
Talaromyces marneffei ATCC 18224 Cu Zn superoxide dismutase SOD1 EEA23919.1
Aspergillus fumigatus Af293 Cu Zn superoxide dismutase SOD1 EAL91677.1
Cu Zn superoxide dismutase GAQ09422.1
Mucor circinelloides f. lusitanicus CBS 277.49 MUCCIRDRAFT 156498 OAD02311.1
Rhizopus delemar RA 99 880 copper zinc superoxide dismutase EIE83111.1
Rhizopus delemar RA 99 880 copper zinc superoxide dismutase EIE83236.1
Cryptococcus neoformans var. neoformans JEC21 copper zinc superoxide dismutase AAW42978.1
Cu Zn superoxide dismutase KIR68394.1
Candida albicans SC5314 Sod5p AOW27120.1
Candida tropicalis MYA 3404 CTRG 01132 EER34272.1
Candida albicans SC5314 Sod4p AOW27119.1
Candida albicans SC5314 Sod6p AOW27078.1
Candida tropicalis MYA 3404 CTRG 01087 EER34227.1
Aspergillus fumigatus Af293 cytosolic Cu Zn superoxide dismutase putative EAL90492.1
Aspergillus lentulus cell surface Cu only superoxide dismutase 5 GAQ10783.1
Scedosporium apiospermum SAPIO CDS4327 KEZ43706.1
Cladophialophora bantiana CBS 173.52 Z519 03626 KIW95045.1
Talaromyces marneffei ATCC 18224 cytosolic Cu Zn superoxide dismutase putative EEA20470.1
Verruconis gallopava PV09 08882 KIV99456.1
Scedosporium apiospermum Cytosolic cu zn superoxide dismutase KEZ44265.1
Verruconis gallopava PV09 02487 KIW06807.1
Trichophyton rubrum CBS 118892 TERG 08969 EGD91757.1
Coccidioides immitis RS Cu Zn superoxide dismutase EAS31515.2
Histoplasma capsulatum NAm1 predicted protein EDN06536.1
Blastomyces dermatitidis ER 3 Cu Zn superoxide dismutase OAT02841.1
Blastomyces dermatitidis ER 3 Cu Zn superoxide dismutase variant OAT02842.1
