# Supplementary material for: Diversity of Cell Wall Related Proteins in Human Pathogenic Fungi
Source: J Fungi (Basel). 2017 Dec 29;4(1):6. doi: 10.3390/jof4010006 (PMC5872309; doi:10.3390/jof4010006)
Supplement: Supplementary file 1 [file jof-04-00006-s001.zip › jof-252817-final-supplementary materials/Dataset S3_trees_pdf/Tos1.pdf]

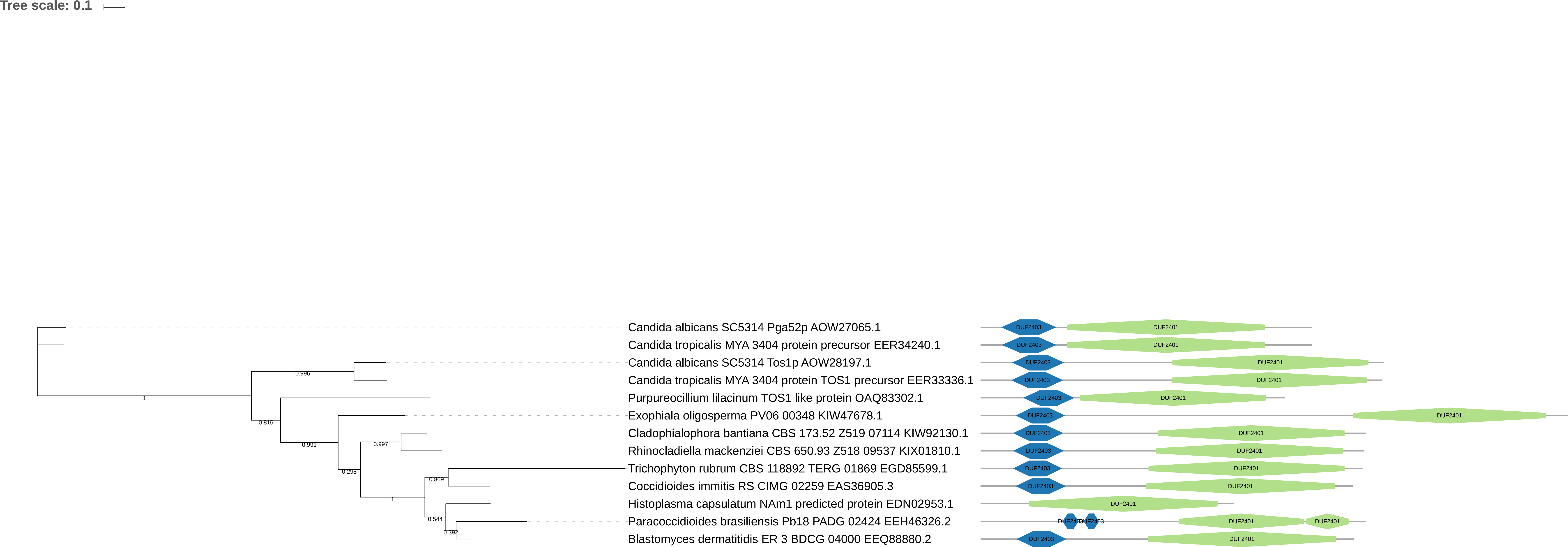
Tree scale: 0.1
Candida albicans SC5314 Pga52p AOW27065.1
Candida tropicalis MYA 3404 protein precursor EER34240.1
Candida albicans SC5314 Tos1p AOW28197.1
Candida tropicalis MYA 3404 protein TOS1 precursor EER33336.1
Purpureocillium lilacinum TOS1 like protein OAQ83302.1
Exophiala oligosperma PV06 00348 KIW47678.1
Cladophialophora bantiana CBS 173.52 Z519 07114 KIW92130.1
Rhinocladiella mackenziei CBS 650.93 Z518 09537 KIX01810.1
Trichophyton rubrum CBS 118892 TERG 01869 EGD85599.1
Coccidioides immitis RS CIMG 02259 EAS36905.3
Histoplasma capsulatum NAm1 predicted protein EDN02953.1
Paracoccidioides brasiliensis Pb18 PADG 02424 EEH46326.2
Blastomyces dermatitidis ER 3 BDCG 04000 EEQ88880.2
0.996
1
0.816
0.991
0.997
0.298
0.869
1
0.544
0.392
DUF2403
DUF2401
