# Supplementary material for: Diversity of Cell Wall Related Proteins in Human Pathogenic Fungi
Source: J Fungi (Basel). 2017 Dec 29;4(1):6. doi: 10.3390/jof4010006 (PMC5872309; doi:10.3390/jof4010006)
Supplement: Supplementary file 1 [file jof-04-00006-s001.zip › jof-252817-final-supplementary materials/Figure S1-4.docx]

Article

Diversity of Cell Wall Related Proteins in Human Pathogenic Fungi

Anna Muszewska *, Sebastian Piłsyk, Urszula Perlińska-Lenart and Joanna S. Kruszewska

Institute of Biochemistry and Biophysics, Polish Academy of Sciences, 02-792 Warsaw, Poland; seba@ibb.waw.pl (S.P.); lenart@ibb.waw.pl (U.P.-L.); jsk@ibb.waw.pl (J.S.K.)

*****	Correspondence: musze@ibb.waw.pl; Tel.: +48-592-57-59

**Figure S1.** Synthesis of GPI anchor in the ER.

**Figure S2.** *N*-glycosylation schema.

**Figure S3.** Further steps of *N*-glycosylation and mannan formation.

**Figure S4.** *O*-mannosylation schema.
